# Pseudo-Symmetric Assembly of Protodomains as a Common Denominator in the Evolution of Polytopic Helical Membrane Proteins

**DOI:** 10.1007/s00239-020-09934-4

**Published:** 2020-03-18

**Authors:** Philippe Youkharibache, Alexander Tran, Ravinder Abrol

**Affiliations:** 1 Cancer Data Science Lab, Center for Cancer Research, National Cancer Institute, National Institutes of Health, Bethesda, MD USA; 2 Department of Chemistry and Biochemistry, California State University, Northridge, CA USA

**Keywords:** Protein structure, 7-transmembrane, 7TMH, 3TMH, Triple helix bundle, Pseudo-symmetry, Membrane proteins, MFS, SWEET, PnuC, TRIC, FocA, Aquaporin, GPCR

## Abstract

The polytopic helical membrane proteome is dominated by proteins containing seven transmembrane helices (7TMHs). They cannot be grouped under a monolithic fold or superfold. However, a parallel structural analysis of folds around that magic number of seven in distinct protein superfamilies (SWEET, PnuC, TRIC, FocA, Aquaporin, GPCRs) reveals a common homology, not in their structural fold, but in their systematic pseudo-symmetric construction during their evolution. Our analysis leads to guiding principles of intragenic duplication and pseudo-symmetric assembly of ancestral transmembrane helical protodomains, consisting of 3 (or 4) helices. A parallel deconstruction and reconstruction of these domains provides a structural and mechanistic framework for their evolutionary paths. It highlights the conformational plasticity inherent to fold formation itself, the role of structural as well as functional constraints in shaping that fold, and the usefulness of protodomains as a tool to probe convergent vs divergent evolution. In the case of FocA vs. Aquaporin, this protodomain analysis sheds new light on their potential divergent evolution at the protodomain level followed by duplication and parallel evolution of the two folds. GPCR domains, whose function does not seem to require symmetry, nevertheless exhibit structural pseudo-symmetry. Their construction follows the same protodomain assembly as any other pseudo-symmetric protein suggesting their potential evolutionary origins. Interestingly, all the 6/7/8TMH pseudo-symmetric folds in this study also assemble as oligomeric forms in the membrane, emphasizing the role of symmetry in evolution, revealing self-assembly and co-evolution not only at the protodomain level but also at the domain level.

**Electronic supplementary material:**

The online version of this article (10.1007/s00239-020-09934-4) contains supplementary material, which is available to authorized users.

## Introduction

Structural pseudo-symmetry in protein domains has been observed since the early days of structural biology. Ferredoxin, Myohemerythrin, Serine and Aspartyl proteases, the TIM (Triose-phosphate-isomerase) barrels, Immunoglobulins, and the Rossmann fold were among the first crystal structures solved. They all exhibit internal pseudo-symmetry (Blundell et al. 1979; Hendrickson and Ward 1977; McLachlan 1972, 1987). As these structures appeared, they corroborated earlier sequence-based observations of possible ancestral gene duplications within today’s genes (Barker et al. 1978; Delhaise et al. 1980; Eck and Dayhoff 1966; Urbain 1969). This defined, without naming it, what we now call *protodomains*, issued from ancestral protogenes. *A protodomain (or protofold) is a supersecondary structure that by its duplication, symmetry operations (and linkers) can generate a structural domain (tertiary fold).*

It is interesting to note that some of these pseudo-symmetric structural domains, characterized early, turned out to be today’s superfolds, some of the most diversified and prototypical folds. In the SCOP classification (Chandonia et al. 2017; Lo Conte et al. 2000), they are denoted (see Table 1) **a.24** (the Myohemerythrin or 4-helix bundle fold) with 28 superfamilies (SFs); **b.1** (The Immunoglobulin fold) with 28 SFs; **c.1** (the TIM barrel) with 33 SFs; and **d.58** (the Ferredoxin fold) with 59 SFs. **The fact that the most diversified folds are pseudo-symmetric suggests a strong evolutionary link between pseudo-symmetry and functional diversification**. We had performed a census of pseudo-symmetry in the currently known universe of protein domains that shows this evolutionary link for ~ 20% of known structural domains (Myers-Turnbull et al. 2014) (Table 1).

**Table 1:** Pseudo-symmetry within Fold classes

Fold class	# Folds In class	# SFs In class	% SFs w/ symmetry	Most diversified Fold in class	# SFs In Fold
A—All Alpha	284	507	19%	a.24—4-Helix bundle	28
B—All Beta	174	354	25%	b.1—Ig fold	28
C—Alpha + Beta	147	244	17%	c.1—TIM barrel	33
D—Alpha/Beta mixed	376	551	14%	d.58—Ferredoxin	59
F—Membrane Proteins	57	109	24%	f.13—GPCRs	1

(According to SCOP 1.75). For each of the five classes of folds in SCOP (A, B, C, D, F), this table lists the number of folds, the number of Superfamilies (SFs), the percentage of SFs deemed symmetrical, the most diversified fold in each class, i.e., folds with the highest number of Superfamilies in each class, and the number of Superfamilies exhibiting pseudo-symmetry in the most diversified fold of each class [see Table S2 in Ref. (Myers-Turnbull et al. 2014) for details]. We have added GPCRs, classified as onefold one-family in SCOP. Technically, it could also be classified as an all-alpha fold (A). The latest SCOPe 2.07 numbers are marginally higher and count 60 membrane protein folds to date (Chandonia et al. 2017).

The pool of known membrane proteins is currently comprised of **71% of ɑ-helical structures**, 19% of β-sheet structures, and the remaining 10% being classified as monotypic. They are, however, classified as one distinct class (F) within SCOP (Chandonia et al. 2017; Lo Conte et al. 2000), regardless of their secondary structure makeup. Overall, they show a higher pseudo-symmetry rate (24%) than most classes (Table 1), but membrane proteins, as a “class,” pose a challenge for an accurate estimation of pseudo-symmetry. That pseudo-symmetry rate number is likely to be a minimum, as the criteria used in the original census underestimated the number of symmetric superfamilies in SCOP to avoid false positives (Myers-Turnbull et al. 2014). With less stringent criteria, **we could estimate that ~ 40% of membrane protein structures exhibit pseudo-symmetry, rather than a conservative 24%**, closer to other estimates (Choi et al. 2008; Forrest 2015; Hennerdal et al. 2010).

Symmetry in quaternary structures is pervasive and has been widely studied (Goodsell and Olson 2000; Levy et al. 2006; Rose et al. 2015), as compared to symmetry in tertiary structures. The latter could in fact be described as a pseudo-quaternary organization of protodomains (Myers-Turnbull et al. 2014; Youkharibache 2019). A recent biophysical study on the ClC chloride transporter found that the transporter is made up of two halves that fold independently as stable subunits, suggesting an evolutionary history of a stable protodomain that duplicated (Min et al. 2018).

While the pseudo-symmetric organization of domains points to a clear mechanism of duplication and self-assembly of protodomains, nothing at the moment can help point to an assembly mechanism of arbitrary pre-folded subdomains in the creation of domains/folds, as sequence-level signature of protodomain duplication is either very weak or non-existent. In fact, some authors have been trying to identify a set of “fragments” forming a structural “vocabulary” of ancient peptides at the origin of the formation of current domains (Alva et al. 2015), led by a belief that “the assembly from non-identical fragments may have been one of the primary forces in the evolution of domains” but, to their surprise, they “did not find even one domain that contained two or more different fragments from their set of base fragments.” They found “instead that fragments either form their folds by repetition or in single copy, decorated by heterologous structural elements, finding the reasons for the lack of fragment combinations unclear”, adding: “While we were unable to detect fragment combinations, repetition is wide-spread” (Alva et al. 2015). This is consistent with our findings in the current study on protodomains, which are repetitive supersecondary structures (seen as “fragments”) that self-assemble symmetrically to form tertiary domains. They are also highly idiosyncratic and can be considered a signature of pseudo-symmetric domains.

### Protodomain Hypothesis in TMH Proteins

**Fig. 1:**
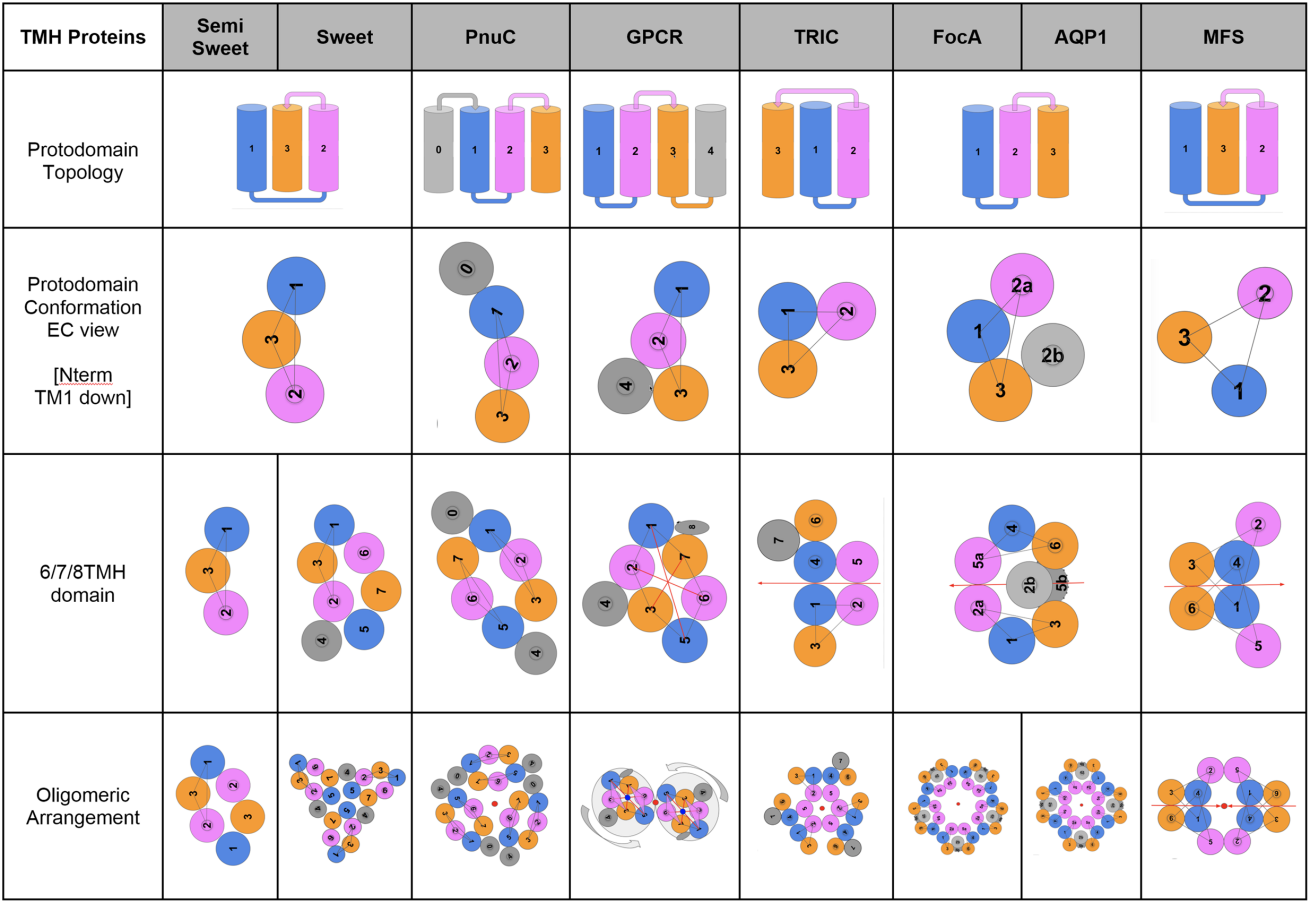

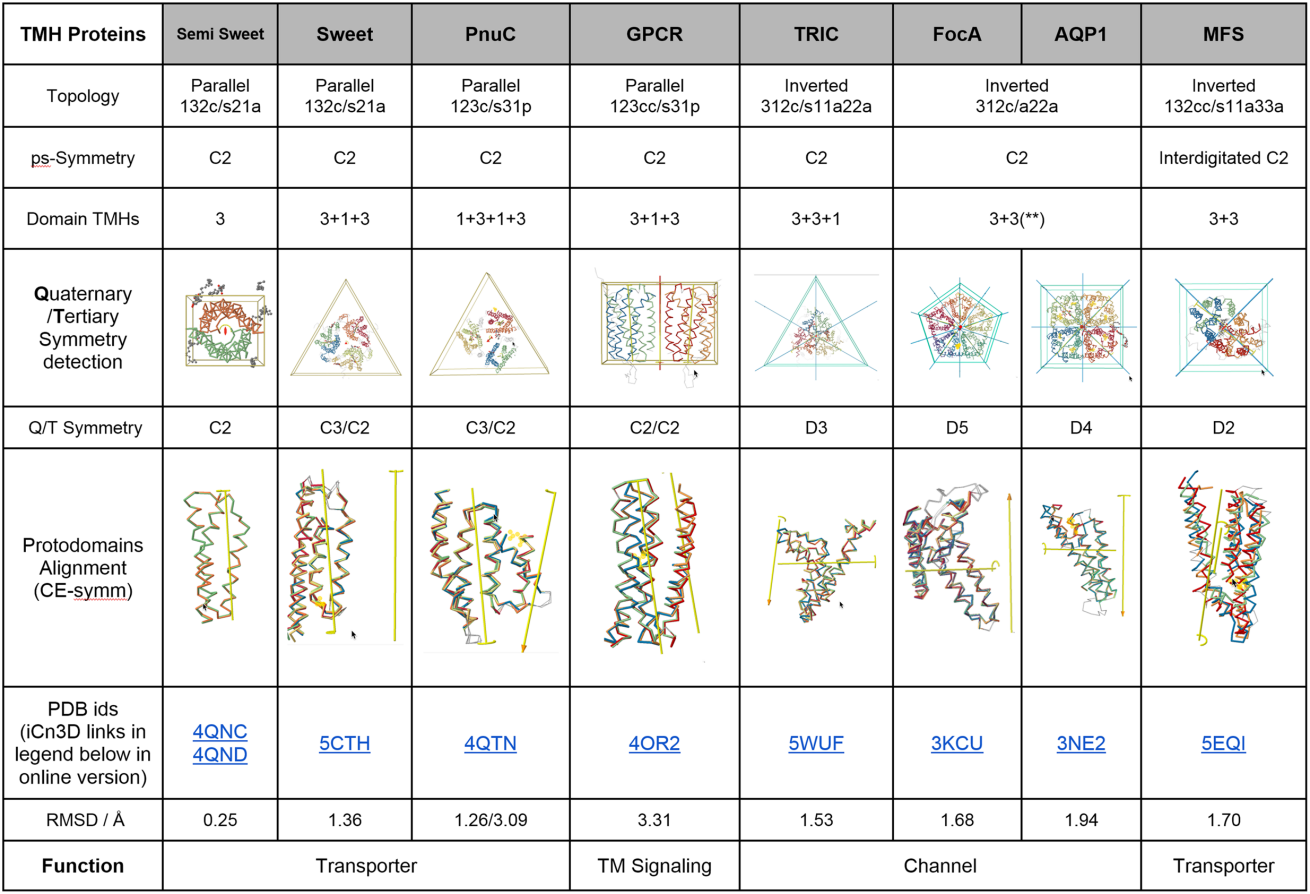
Pseudo-symmetric domains formed from 3/4TMH protodomains—see main text for explanations. Color coding of the TM helices in each 3TMH protodomain with BLUE, MAGENTA, and ORANGE, as mentioned in the methods section, enables a quick equivalence between the individual helices from each protodomain. For example, the first and second 3TMH protodomains of a GPCR (TM1–TM2–TM3 and TM5–TM6–TM7, respectively) are colored as TM1/TM5 in BLUE, TM2/TM6 in MAGENTA, and TM3/7 in ORANGE, showing the equivalence of the TM helix pairs TM1–TM5, TM2–TM6, and TM3–TM7. GRAY is used for an additional helix in 7TMH and for the 4th helix in 4TMH protodomains. RMSD reported for optimized structural alignment of protodomains (see Methods). In the case of PnuC, a 3TMH alignment leads to 1.26 Å, while for 4TMH it rises to 3.09 Å (including TM0/TM4—see text for more details). The iCn3D links for the PDB ids are available in the online version as: 4QNC/4QND, 5CTH, 4QTN, 4OR2, 5WUF, 3KCU, 3NE2, 5EQI

**Fig. 2:**
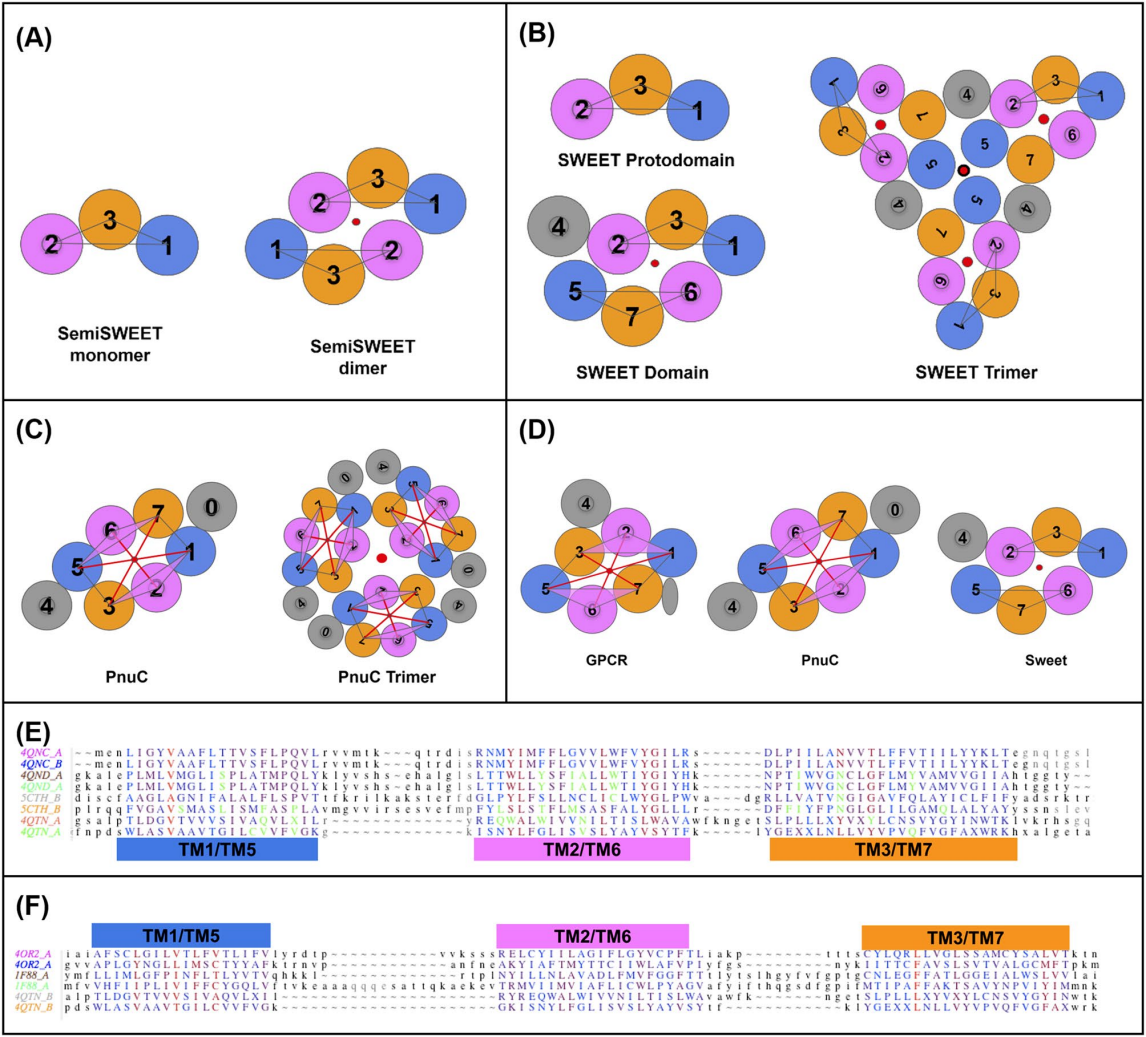
SemiSWEET, SWEET, PnuC and GPCRs. **a** SemiSWEET 3TMH monomer and dimer. **b** SWEET 3TMH protodomain and a 7TMH SWEET domain formed from 3TMH protodomains (left) and SWEET trimer (right). **c** PnuC 7/8TMH domain (left; see text) and trimer (right). **d** GPCR vs. PnuC vs. SWEET—comparison of topologies. **e** Structure-based sequence alignments. SemiSWEET/SWEET/PnuC—structure-based sequence alignment of 3TMH from SemiSWEET dimer (4QNC; 4QND), SWEET (5CTH), PnuC (4QTN). Ligand binding residues are in green, conserved/similar residues are in red. Structural alignment of TMHs of PnuC (pdbid 4QTN) to SemiSWEET (4QNC) and SWEET (5CTH) protodomains gives an RMSD of 6.5 Å. So, PnuC does not appear to be a structural homolog of SWEET at the protodomain level. However, from the very simple schematic representation, one can see that TMH167 and TM523 match the SWEET/SemiSWEET protodomains. If we ignore TM1 and TM5 in PnuC, the RMSD on other 2 helices goes down to 1.71 Å and 2.47 Å, respectively. The RMSD between PnuC's 3-helix combination 167 and SemiSWEET 3TMH is 1.87 Å, suggesting a structural homology and possible evolutionary link (see text). **f** GPCR vs. PnuC—structure-based sequence alignment of 3TMH from a class C GPCR (4OR2) vs. a class A GPCR (Rhodopsin) (1F88) vs. PnuC (4QTN). The RMSD values of multiple protodomains aligned vs. the first one (4OR2-1) are 3.63, 2.12, 3.29, 3.29, 3.34 Å for 4OR2-2, 1F88-1&2, 4QTN-1&2, respectively. However, if one excludes TM2 from the structural alignment the RMSD is now 2.09, 1.17, 2.77, 2.61, 2.79 Å, respectively

A recent analysis of known membrane proteins showed that polytopic (also called multi-pass) helical membrane proteins are dominated by 7TMH proteins [see Fig. 2b in (Bausch-Fluck et al. 2018)]. The 4TMH and 12TMH proteins are distant seconds. So, in this study, we have mainly focused on 6TMH, 7TMH, and 8TMH proteins (Fig. 1) as 6TMH/8TMH proteins share the same evolutionary path as 7TMH proteins.

Structurally, most individual TM helices look alike and are difficult to distinguish. Some helices may have breaks and tilts and may be perceived as unique, but unless they have a very high sequence homology, no conclusion can be drawn from a structural comparison of individual TM helices. More complex supersecondary constructs (made up of 2 or more TM helices) can, however, show structural similarities that may point to an evolutionary duplication.

### 2TMH Protodomains → 4TMH and 6TMH Proteins

The first step up in complexity is the smallest “supersecondary” helical structure we can envision: a Helix-Turn-Helix (2TMH) motif. In fact, at that level, we can already start seeing 2TMH elements combining symmetrically to provide domains/folds, such as a 4-transmembrane helix (4TMH) bundle through intragenic duplication. This is similar to globular proteins with a hemerythrin fold (Hendrickson and Ward 1977), the most functionally diversified helical fold (SCOP a.24—Table 1). In reality, almost any 4-helix bundle (4TMH) can be seen as a symmetrically organized duplicated Helix-Turn-Helix 2TMH “protodomain” (C2 symmetry). A purely geometrical analysis will, in many cases, show an even higher D2 symmetry for antiparallel (up/down) bundles. The Helix-Turn-Helix 2TMH motif can also lead to a 6TMH domain. A clear example of a C3 tertiary symmetry through a triplication of a 2TMH protodomain has been observed in the case of the proton-gated urea channel (PGUC) (Strugatsky et al. 2013), with a parallel membrane topology. These 4TMH and 6TMH proteins can also oligomerize symmetrically, as pentameric ligand-gated ion channels (pLGICs) with 4TMH domains that assemble as pentameric oligomers (C5 symmetry) and PGUCs with 6TMH domains that assemble as hexameric oligomers (C6 symmetry).

### 3TMH and 4TMH Protodomains → 6TMH, 7TMH, and 8TMH Proteins

The next step up in complexity for helical protodomains is a 3-helix motif (3TMH), or Triple helix bundle (THB), which upon intragenic duplication can lead to a 6 (or 7)-helix bundle (6/7TMH) or even bigger (Khan and Ghosh 2015). As an example, bacterial SemiSWEET is a 3-helix monomer that homodimerizes to form a 6-helix quaternary structure binding a sugar in the central cavity lying on the homodimer symmetry axis (Fig. 2b, also see Fig. 2e for corresponding structure-based sequence alignments). That arrangement is strictly conserved in the eukaryotic 7TMH SWEET domain, a pseudo-symmetric tertiary domain. This provides evidence for duplication and fusion of two 3TMH protodomains (Fig. 2b, e). In this case, a long linker between 3TMH protodomains, long enough to form a membrane-spanning helix, enables the formation of pseudo-symmetric 7TMH proteins with a “parallel topology,” i.e., with the symmetry axis orthogonal to the membrane planes. In the case of 7TMH protein domains that exhibit symmetry, one can, as for SWEET, envision a helical long linker (TM4) keeping a parallel topology between two 3TMH protodomains (TM123/TM567). One can also envision the formation of a 7TMH domain from the duplication and fusion of two 4TMH protodomains with the attrition of one helix at the N or C terminus or possibly in the middle. The duplication fusion event that may well have originally duplicated a 4TMH protodomain rather than 3TMH has been previously suggested for GPCRs (Saier 2016; Yee et al. 2013).

The study of the evolutionary history of TMH proteins in terms of potential protodomain duplications has been predominantly based on sequence homology between the two protodomains, which has had some success (Barker et al. 1978; Taylor and Agarwal 1993; Yee et al. 2013). Sequence-level signatures of protodomain duplication can be weak depending on the when the duplication occurred and how much a protein family may have evolved, making sequence-based methods miss many potential protodomains. In addition, these methods can be limited in providing mechanistic insight into intragenic duplication and the role of structural as well as functional constraints in protein evolution. The maturation of the structural biology methods for membrane proteins has led to an increase in the number of available structures for TMH proteins in recent years (Allen 2019; Zhang and Cherezov 2019). Even though a majority of these structures are static snapshots of protein structure that do not capture protein dynamics (Standfuss 2019), these structures can complement sequence-based methods to identify protodomains, and can begin to provide testable hypotheses on evolutionary pathways taken by TMH proteins during and after their formation from protodomains. In this study, we perform a parallel sequence–structure–function analysis of a set of diverse α-helical transmembrane protein families with 6/7/8TMH domains that shows structural evidence of symmetrically organized 3TMH or 4TMH protodomains at the tertiary level (domains) and at the quaternary level. From an evolutionary standpoint, the structural evidence of protodomain duplication is strong across functionally diverse TMH protein families. It provides a generalizable framework for potential mechanisms of protodomain arrangement during duplication and how different structural subdomains of these proteins evolved under specific conformational and functional constraints.

## Methods

### Pseudo-Symmetry Protodomain Analysis (PSPA) Method

We have reviewed this method in detail elsewhere (Youkharibache 2019). The method involves two initial steps: **Symmetry detection** and **Protodomain delineation**. For any input structure, tertiary structure symmetry detection gives a first delineation of protodomains and a symmetry point group. Quaternary structure symmetries can also be determined concurrently, hence the method enables a multi-level symmetry detection. A follow-up step is usually required to optimize structural alignment and protodomains delineation. This then opens the door to any desired analysis that a structural alignment of tertiary and/or quaternary structures may enable.

#### Symmetry Detection

A few computer programs can detect internal pseudo-symmetry at the tertiary structure level (Kim et al. 2010; Myers-Turnbull et al. 2014) (See Table 1). The program CE-symm, in its newest version, allows simultaneous quaternary and tertiary symmetry analysis of multidomain complexes (https://github.com/rcsb/symmetry). However, there are many cases where we have to adapt program parameters to detect symmetry and obtain structurally aligned protodomains, depending on departure from perfect symmetry and structure quality. In some cases, one has to use interactive alignment software to align a domain onto itself, which requires a visual inspection at each and every step. This is particularly true for GPCRs that present a wide range of structures, resolution, co-crystallization domains, and conformational states. In all cases we optimize the delineation of protodomains through interactive structural alignment for accuracy.

#### Delineation of Protodomains: Optimization Through Structural Alignment

The alignment of protodomains can identify important residues that may be internally conserved for either a structural (folding and assembly) or a functional reason, for example for ligand binding. The level of overall internal sequence conservation is usually low. It is a common pattern in most pseudo-symmetric domains, unlike most domain or family-level sequence and structure conservation. In the examples considered in this paper, TRICs show such a clear duplication pattern of protodomains with the highest percent identity (29%) and no insertion/deletion between the duplicated protodomains (Kasuya et al. 2016; Su et al. 2017) (see alignments in Fig. S1).

Matching sequence patterns between protodomains resulting from their structural alignment is similar, at first sight, with any domain-level analysis. However, one should note that an alignment of protodomains is different from a classical domain alignment leading to families and superfamilies. One should not expect “internal” sequence conservation in the same sense. A precise structural alignment of protodomains forming a domain can identify conserved residues, yet few invariant (“internally conserved”) residues at symmetrically equivalent positions may be invariant across domains in a family or superfamily, but if they are, they will no doubt bear a particular significance. These cases are rare, and it may be best to talk about coincidence rather than conservation until further evidence is gathered. Protodomains have not evolved separately and have not conserved residues for functional or folding reasons in the same way proteins in a family would. On the contrary, protodomains in a domain have co-evolved within a domain to reach an idiosyncratic function, most of the time at their interfaces, while maintaining a pseudo-symmetric fold. The low level of “internal sequence conservation” observed in most cases is likely due to the second (duplicated) protodomain evolving under different functional and structural constraints compared to the first (original) protodomain.

Pseudo-symmetry provides a framework for hierarchical structural analysis. It enables the reverse engineering of protein domains from well-defined parts in the context of an evolutionary and/or functional analysis. Hence, these parts are called protodomains. This should enable a better understanding of molecular self-assembly and a co-evolutionary analysis of protodomains as well their interfaces. This will catalyze new developments of the analysis methods in the future, and the rapidly growing number of available GPCR structures should provide a rich dataset to envision the use of machine learning to identify co-evolution patterns within that pseudo-symmetric framework.

The Root Mean Square Deviation (RMSD) has been used as the criterion of choice to optimize alignments. It represents a good quantitative measure of structural similarity. A small RMSD denotes a strong structural similarity (homology). In comparing helical structures, we consider a RMSD lower than 3.5 Å to describe a good overall structural match. The majority of our domain or protodomain alignments on GPCRs lie between 2.5 and 3.5 Å RMSD. A note of importance is that within this range, we can have alternative sequence alignments. It is common in helical alignments to see translations of helices along their helix axis, shifting residues in positions ± 4. In fact, most helical protein structural alignments match domains within ~ 3 to 4 Å in RMSD (for α-carbon traces) even in cases of high sequence homology, and a helical turn translation of a helix may not drastically change an overall RMSD.

#### Software Programs

Two programs allow the automatic detection of pseudo-symmetry in protein domains, SYMD (Kim et al. 2010) and CE-symm (Myers-Turnbull et al. 2014). We use the latter for automatic detection. The program does a good job at capturing both quaternary and tertiary levels of symmetry. Some results are summarized in the Fig. 1 for all the proteins studied in this paper. They all show two levels of symmetry, one tertiary and one quaternary that in the case of inverted membrane topologies do combine to give dihedral symmetries. However, GPCR pseudo-symmetry is not detected by the software in most cases, except for example for 4OR2 (see below). Interactive structure alignments (optimization) for all structures in this paper are performed with the Cn3D software (Madej et al. 2012; Wang et al. 2000). All GPCR protodomain pairwise and multiple sequence/structure alignments are optimized with Cn3D. Domain-level structural alignments can be readily available from NCBI VAST structural database (Madej et al. 2012), and from VAST + (Madej et al. 2014) for quaternary assemblies alignments. VAST + alignments capture conformational changes in tertiary domains and quaternary assemblies, in particular for GPCR conformational changes. All these structures can now be visualized with web-based visualization and analysis software iCn3D (Wang et al. 2020) developed at NCBI and available as open source (https://github.com/ncbi/icn3d). iCn3D allows the creation and exchange of annotated 3D structure visualizations in parallel with sequence (1D) in particular. The iCn3D visualization links are given in Fig. 1 legend and supplementary material for all the proteins considered in this study.

#### Notation, Coloring, and Visualization

Naming and numbering of secondary structure elements with repeats can be confusing, as elements are numbered in sequence, within a protodomain, within a domain, and within a multidomain protein (such as MFS). Hence that notation has to vary depending on context. We cannot avoid a certain imprecision due to the multiple numbering of a given element. Also numbering in sequence may not match what is used in naming different topologies adopted by 3TMH, such as 123, 231, 312.

Color is the most important element of distinction and recognition used in figures. In a 3TMH protodomain, we use sequential colors BLUE, MAGENTA, and ORANGE for transmembrane helices that we name TM1, TM2, TM3, respectively. Whatever number a protodomain’s TMH ends up having, such as TM5, TM6, and TM7 in 7TMH domains, they will be colored blue, magenta, and orange (in that order), which makes it easy to visually spot and appreciate protodomain duplications.

We use schematic 2D projections from the extracellular (EC) side for visualization of protein transmembrane domains, except where specified. These are idealized as if TMHs would be exactly perpendicular to the membrane, while in reality they are tilted with respect to the membrane normal; hence in some cases, we could have two neighboring helices that are orthogonal to each other in the membrane. This could result in principle in different views from the EC and the intracellular (IC) sides, yet it is not the case in the proteins examined in this study. Let us also note that a clockwise arrangement of helices seen from the EC side would appear as counterclockwise from the IC side, its mirror image. 3D visualization is available through iCn3D (Wang et al. 2020) web links in the Fig. 1.

#### Protein Structure Classification

Two major classifications SCOP (Chandonia et al. 2017; Lo Conte et al. 2000; Murzin et al. 1995) and CATH (Dawson et al. 2017; Orengo et al. 1997) have been used for a long time, and more recently ECOD (Cheng et al. 2014; Schaeffer et al. 2017). We chose SCOP for its fold classification, based on geometrical criteria and manual curation, as “The method used to construct the protein classification in SCOP is essentially the visual inspection and comparison of structures, though various automatic tools are used to make the task manageable” (Murzin et al. 1995). This, in essence, has been our simple but accurate approach to pseudo-symmetry analysis and protodomain delineation through self-protein alignment (see earlier), especially for GPCRs, for two reasons. First, at this stage, and even more when we started, no automatic tool will identify their symmetry and protodomains alignments accurately and systematically. Second, evolution of membrane proteins may have, precisely, a particular geometrical drive that transcends current views of sequence-based evolutionary paths. Hence the notion of fold and protofold in our context constitutes a central geometrical Element (Euclid) of major importance.

### Evolutionary Structure Analysis of Different Transmembrane Protein Families

Structures related to the specific protein family being analyzed are pulled from the PDB for GPCRs and for other TMH proteins from the NCBI’s Conserved Domains Database (CDD) (Marchler-Bauer et al. 2003, 2017, 2015). The list of proteins and PDB ids used for each family is provided in the Supplement File SF1. The corresponding fasta sequences are pulled from Uniprot (UniProt Consortium 2018), and input into our PredicTM program (Goddard et al. 2010) that implements the hydropathy analysis (von Heijne 1992) to generate membrane-fasta (mfta) files containing hydrophobic TM regions. Each protein’s TM regions are extended based on the protein data bank (PDB) (Burley et al. 2017) structures. The hydrophobic centers are determined by looking at the PDB structures aligned by the OPM database (Lomize et al. 2012) to an implicit membrane [middle of the membrane defined by the *x*–*y* plane (same as *z* = 0 plane)] and selecting one residue as a hydrophobic center in each TM domain with the Cα *z*-coordinate closest to zero (designated *h* in Supplementary File SF2). The TM regions and hydrophobic centers are recorded in the protein’s mfta file. Then, the CDD protein family’s sequence alignments are downloaded from NCBI’s CDD website. This sequence alignment consists of the ten most diverse proteins in the family. Using HMMER, the proteins with PDB structures are aligned to the existing CDD multi-sequence alignment. Based on the alignment, a consensus for the TM regions and hydrophobic centers is reached using the proteins with PDB structures. This consensus is used to assign the TM regions and hydrophobic centers in the respective mfta files for all proteins in the new CDD multi-sequence alignment. After using a custom script to cut up the sequences using the TM regions and hydrophobic centers, the resulting fasta files are consolidated into combined fasta files for each TM. Each TM’s intracellular-facing half and extracellular-facing half are determined from the hydrophobic centers described earlier. Finally, the consolidated multi-sequence fasta files are run through a custom alignment similarity scoring function defined below based on the blosum62 matrix and the scores are recorded for each TM and for each EC and IC half. The TM lengths of the corresponding TMs across the two protodomains were not matched to each other so that we can capture the true divergence of the corresponding TM regions across the two protodomains. A handful of TM regions were not present in the CDD alignments, in which case those were aligned manually.

#### Similarity Scoring of the Structure-Based Sequence Alignments of the Intracellular and Extracellular-Facing TM Halves

The similarity scoring program divides the sequences in an alignment into vertical columns. Each amino acid in a column is compared to others in the same column and given a score. The scores are derived from custom scores based on the blosum62 matrix. A blosum62 matrix score of 4 or greater is assigned a custom score of 1. The blosum62 matrix scores of 3, 2, 1, and 0 are assigned custom scores of 0.75, 0.50, 0.25, and 0.125, respectively. All negative blosum scores are assigned a custom score of 0. This scoring is repeated for every column in the alignment. The scores are then added together and divided by the number of comparisons. After multiplying the result by 100, we get the similarity percentage between all sequences in the alignment, which captures how much each respective domain has diverged, the smaller the number, the higher the divergence.

## Results and Discussion

### Protodomain Duplication Evidence in the Tertiary Fold of 6/7/8TMH Proteins

The transmembrane helical protein families analyzed in this study are composed of 6, 7, or 8 helices (6/7/8TMH) and are presented in Fig. 1. These families cover diverse functions such as transporters (SemiSWEET, SWEET, PnuC, MFS), channels (TRiC, FocA, Aquaporin), and signaling receptors (GPCRs). They represent a variety of folds that are associated with a wide range of functions and are formed by duplication of 3/4-helix protodomains. They present either an inverted membrane topology (TRIC, Aquaporin, FocA, MFS) or a parallel topology (SWEET, PnuC, GPCR). They possess an axis of symmetry at the domain level, either perpendicular or parallel to the membrane planes (bisecting the membrane), respectively, according to that topology. The supplementary Figures S1 through S3 show the corresponding protodomain sequence alignments and Figures S4 through S9 give detailed structural representations. Figure 1 legend and supplementary Table S1 provide web links to 3D structure representations.

Figure 1 shows schematic representations of protodomains of the TMH protein families. For each, it shows the tertiary domain (fold) formed from these protodomains (protofolds) by duplication and symmetric assembly, as well as the quaternary arrangement of tertiary domains through another level of symmetry. Symmetry groups for the tertiary/quaternary structures are indicated. While in all of the domains considered, protodomains assemble with a C2 pseudo-symmetry in forming domains, domains themselves assemble symmetrically in forming quaternary structures, ranging from C2 to C5 symmetry in our examples, with the axis of symmetry being perpendicular to the membrane. There are two levels of cyclic symmetry: tertiary and quaternary. For domains with inverted topologies (Duran and Meiler 2013), quaternary and tertiary (Q/T) symmetry axes can combine to lead to dihedral symmetry groups (D2 to D5 in our examples), while in parallel topologies, quaternary and tertiary levels of symmetry axes are parallel to each other (C3/C2 and C2/C2 for Q/T symmetry groups). MFS represents a special case: the domain has an inverted topology and two domains are fused, presenting D2 symmetry at the tertiary level.

The TM helices are color-coded to show the equivalence of protodomains and their individual helices (BLUE, MAGENTA, and ORANGE, as mentioned in the methods section). The topology is indicated considering both protodomains relative to each other, going beyond the simple parallel vs. inverted description used for symmetric membrane proteins, by considering protodomain conformations and specific helix pairing in the protodomain self-assembly of a domain. For example, a SWEET protein protodomain will have 3 helices (**1,2,3**) forming in the order **132 clockwise** (**c**). Here, protodomains combine through symmetric assembly of **helix 2** of protodomain 1 and **helix 1** of protodomain 2 and vice versa (**s21**) in an antiparallel (up/down) manner (**a**). Hence in the example of SWEET, we use the notation **132c/s21a** to describe the protodomain topology-conformation/assembly. For PnuC, it is **123c/s31p,** a clearly different protodomain conformation (**132c** vs **123c**), assembling also symmetrically but through a different helix pairing (**s21a** vs **s31p**), and relative orientation (antiparallel vs. parallel). In the case of GPCRs, the topology-conformation/assembly is **123cc/s31p,** with yet another variant of counterclockwise (**cc**) vs. clockwise (**c**) protodomain organization of helices, as seen from the extracellular side. This circularity will also be distinctive at the domain level: GPCR domain is counterclockwise, while PnuC is clockwise (Fig. 2d). Using this notation it is easy to find topological similarities across different proteins, but also to distinguish them, if there is any way to relate these domains, as has been done in the literature (Jaehme et al. 2015, 2016; Saier 2016; Yee et al. 2013), one may invoke a “conformational evolution” mechanism (see later).

Supplementary Table S2 reports the sequence identity in parallel with the RMSD of protodomain structures for structure-based sequence alignment of the protodomains in pseudo-symmetric proteins: SWEET, PnuC, TriC, Aquaporin, and FoCA. For these families, the structure and sequence matches are high enough to unequivocally call for a pseudo-symmetric assembly of original protodomains, with an average sequence identity across protodomains of ~ 20% (ranging between 10 and 35%) and average RMSD of ~ 1.8 Å (ranging between 1.2 Å and 3.0 Å). Table S3 reports the same numbers for a selected number of GPCRs, matching the proposed pseudo-symmetric TM123/TM567 protodomains. Here the average RMSD is 3.0 Å (ranging between 2.5 and 3.5 Å), and the average sequence identity is 14% (ranging between 10 and 20%). When considering a potential alternate 3TMH bundle of TM456 instead of a symmetry matching TM567 to compare to TM123 within a GPCR [similar to what was done before (Hennerdal et al. 2010)], the average sequence identity drops from ~ 14 to ~ 9% on average for the alternate 3TMH pair comparison, while the average RMSD jumps from ~ 3.0 to ~ 6.5 Å on average. These two measures combined, considering the symmetry relation of protodomains TM123/567 (especially for ligand binding residue position in TM3/TM7, covered later in the discussion), argue in favor of the proposed GPCR protodomains organization.

A note of caution is necessary when looking at similarity ranges in sequence space for unrelated TM helices. A range of 5–15% identity among TM helices could be called the “anti-twilight zone” (by opposition to the very low sequence identity in globular proteins), since many TM helices, even when unrelated by evolution, present fortuitous sequence identities. For our 3TMH GPCR protodomains (TM123/567) [Table S3] we can see a higher sequence identity match overall, but the numbers for many GPCR protodomain sequence matches are in this anti-twilight zone, and we clearly rely on the symmetric structural protodomains matches in the 2.5–3.5 Å range to call for a pseudo-symmetric (TM123/567) organization of GPCRs.

In summary, the structural homology of protodomains, measured by the RMSD of the Cα atoms between them, falls in the 1.26–3.80 Å range (smaller number means higher structural homology, see methods section), while the sequence identity ranges between 7 and 35% for the set of proteins considered in this study. In Fig. 1, **iCn3D weblinks** (Wang et al. 2020) for each protein family give access to **3D visualization** on a computer or a tablet.

Next, we describe the inverted topology and parallel topology cases separately to highlight topo-conformational/assembly similarities and differences of 3TMH protodomains.

### Pseudo-Symmetric Assembly of 3TMH Protodomains in an Inverted Topology

#### Multi-level Symmetries in TRIC, Aquaporin, and FocA

A duplicated 3-helix (3TMH) protodomain can form a 6-helical C2 pseudo-symmetric membrane protein (6TMH) domain with a symmetry axis parallel to the membrane planes going through its center for these proteins (TRIC, Aquaporin, and FocA in Fig. 1, see also Figures S2 for protodomain alignments and Figures S7 and S8 for a close-up view of the structures). This implies a very short linker (or, if long, an extra or intracellular loop according to where the N terminus of second protodomain fuses with the C terminus of the first protodomain). Two symmetrically related protodomains form, in that case (e.g., TM1–TM2–TM3 and TM4–TM5–TM6 for TRIC), which is called an “inverted topology” in membrane protein terminology (Rapp et al. 2007), where the directions of helices TM1 and TM4 are opposite to each other along the membrane normal. Additionally, even with a short linker between two inverted 3TMH protodomains in a symmetric arrangement as in the TRIC family architecture, the resulting 6TMH domain topology is, in fact, supplemented by a 7th TMH at the C terminus (Su et al. 2017) resulting in a 7TMH protein domain.

The structural homology between the two protodomains was measured through their optimized superimposition (Fig. S1). For SWEET, PnuC, TRIC, FocA, and Aquaporin, the protodomains match with an RMSD varying between 1.26 to 2.94 Å (average 1.81 Å), and their sequence identity varying between 11 and 35% (average 20%) (Table S2). TRIC’s two protodomains are the closest to each other in this group of protein families (RMSD = 1.53 Å; % Id = 35%).

The protein families of FocA and Aquaporin share the exact same fold, with the same pattern of internal duplication of a highly idiosyncratic protodomain, where (symmetrically related TM2 and TM5 helices in both protein families have a noticeable break in the middle leading to TM2a/b and TM5a/b reentrant helical segments, see Fig. S8), yet they seem to have lost a common sequence signature that can be detected (Theobald and Miller 2010; Wang et al. 2009), leaving the door open to hypothesize a convergent evolution scenario. We will review this scenario in the discussion below (see following section “Convergent vs Divergent Evolution”).

#### MFS: More Than Inverted … Interdigitated

MFS is a very interesting fold, as it is hierarchical: a 6TMH domain is formed by duplication of 3TMH protodomains with an inverted membrane topology, followed by a domain duplication tying together two domains that assemble through a pseudo-symmetric interface; hence, a well-integrated tertiary complex of dihedral symmetry emerges with 12TMH (see Fig. 1 and Fig. S9). The domain-level assembly is a good example of a pseudo-quaternary association of domains, tethered together by a (quite long) covalent linker. The domain-domain interface is what would be expected in a quaternary dimer interface.

To form a 6TMH domain, the two 3TMH protodomains in MFS are not just inverted, they are also interdigitated and form a C2 symmetric domain. This domain possesses two locally symmetric helix-helix interfaces: TM1–TM1 and TM3–TM3 (**132 cc/s11a/s33a** topology). The second protodomain has a high structural homology to the first protodomain [with RMSD of 1.70 Å (pdbid 5EQI)]. The 3 helices are not packed together; they exhibit a wide spacing between contiguous helices TM1 and TM2 (Forrest 2015) that precisely allows them to interdigitate with an inverted image protodomain, forming a packed domain (Fig. 1 and Fig. S9).

The 3TMH protodomains in TRIC, FocA/AQP1, and MFS display a distinct diversity of topologies (not afforded to 2TMH protodomains) and exhibit conformational flexibility. This flexibility in return also enables the interdigitation of the helices in domain folding of MFS. The SWEET protein, in contrast, presents a case with tightly packed protodomains that should form independent folding units, as will be presented next.

### Pseudo-Symmetric Assembly of 3TMH or 4TMH Protodomains in a Parallel Topology

#### Multi-level Symmetries in SWEET and PnuC

A long linker between 3TMH protodomains, long enough to form a membrane helix, enables the formation of pseudo-symmetric 7TMH proteins with the symmetry axis orthogonal to the membrane planes. The best example is the 7TMH SWEET protein. Its bacterial homolog, called appropriately SemiSWEET, is a 3-helix monomer that homodimerizes to form a 6-helix quaternary structure binding a sugar in its central cavity lying on the homodimer symmetry axis (Figs. 1, 2a, Figs. S4 and S5, also see Fig. 2e for corresponding structure-based sequence alignments). The arrangement is strictly conserved in the eukaryotic 7TMH SWEET domain, which is a pseudo-symmetric domain with its axis of symmetry, sugar binding, and local sequence patterns conserved (Fig. 2b).

The RMSD between two SemiSWEET monomers (pdbid 4QNC) in the dimer are 0.17 Å, to another dimer binding a ligand (pdbid 4QND) 1.29 Å, and to SWEET protodomains (pdbid 5CTH) 1.74 Å, respectively.

This provides evidence for duplication of two 3TMH protodomains, absolutely equivalent to the bacterial 3TMH SemiSWEET domain, to form the eukaryotic 7TMH SWEET membrane protein (Fig. 2b, e). In this case, 2*3TMH = 6TMH + 1TMH Linker.

The vitamin B3 transporter PnuC represents another very interesting case of a 7/8TMH protein that exhibits C2 pseudo-symmetry with evidence of a 4TMH protodomain (Fig. 2e) in a parallel topology. Domains also themselves assemble as trimeric quaternary structure (see Figs. 1, 2c). As a family, PnuC is described as a 7TMH (e.g., Uniprot B8F8B8). The particular PnuC domain structure (PDB: 4QTN—Uniprot D2ZZC1) has 8 TMHs, that we use as an example of a possible 4TMH protodomain duplication (Fig. 2c). It is clear, however, that the 4th helix does not match, structurally, with its proposed symmetric counterpart in a decisive manner. The RMSD of TM0123 vs. TM5678 is 3.09 Å, while if we reduce the protodomain to a 3TMH, then TM123 vs. TM567 RMSD is 1.26 Å (see Fig. 1). The possibility of 4TMH protodomain cannot be ruled out given the potential for conformational evolution of one of the TM helices after duplication. From the very simple schematic representations of SWEET and PnuC (Fig. 2b, c), one can align TM1–TM6–TM7 and TM5–TM2–TM3 to SWEET/SemiSWEET 3TMH with a high structural homology (low RMSD). This is equivalent to a symmetric structural swap of TM1/TM5 as previously proposed (Jaehme et al. 2015). The transporters PnuC and SWEET have effectively been proposed to be evolutionary related. PnuC is seen as a “full-length SWEET homolog” (Feng and Frommer 2016; Jaehme et al. 2014, 2015, 2016).

Divergent structural folds around a putative-related function are not uncommon; they have been observed and named topological isomers, or “topoisomers” (Murzin 1998), where the author notes: “… A simple way of altering a protein fold without a big destabilization is to change its topology while maintaining its architecture.[…] This can be done by the internal swapping of similar helices and strands or by reversing the direction of some of its secondary structures. …”. They further observe: “The close structure conversion of one protein topoisomer into another would require at least partial unfolding” but also that “the folding of different topoisomers of a protein chain is yet to be observed.”

PnuC and SWEET could effectively be considered topological isomers, having a similar structure despite a different topology, and having a similar transport function. One may envision a permutation at the gene duplication level between SWEET and PnuC involving a segment covering 4 helices TM2345. Permutations of secondary structure elements, commonly seen as circular permutations (CPs), conserve 3D structure, i.e., the order of secondary structure elements does not affect the folded structure (Viguera et al. 1995). To our knowledge, however, CPs have not yet been observed in membrane proteins; however, circular and non-circular permutations have been engineered to show remarkable functional resilience of alternate topologies in rhodopsin (Mackin et al. 2014). The mechanism by which such fold changes might occur is unknown. Beyond relating SWEET and PnuC sugar transporters, some authors have gone further in relating GPCRs as PnuC’s topological isomers (Saier 2016; Yee et al. 2013). Figure 2d shows their domain topologies and Fig. 2f shows the comparison of their protodomains. There is weak sequence homology but higher structural homology and no obvious functional relation. This suggests either a coincidental structural convergence or a common evolutionary ancestor 3TMH/4TMH protein. We will propose in the following a possible conformational evolution mechanism that may clarify some of these evolutionary relationships.

#### Structural Pseudo-Symmetry Evidence in GPCRs

**Fig. 3:**
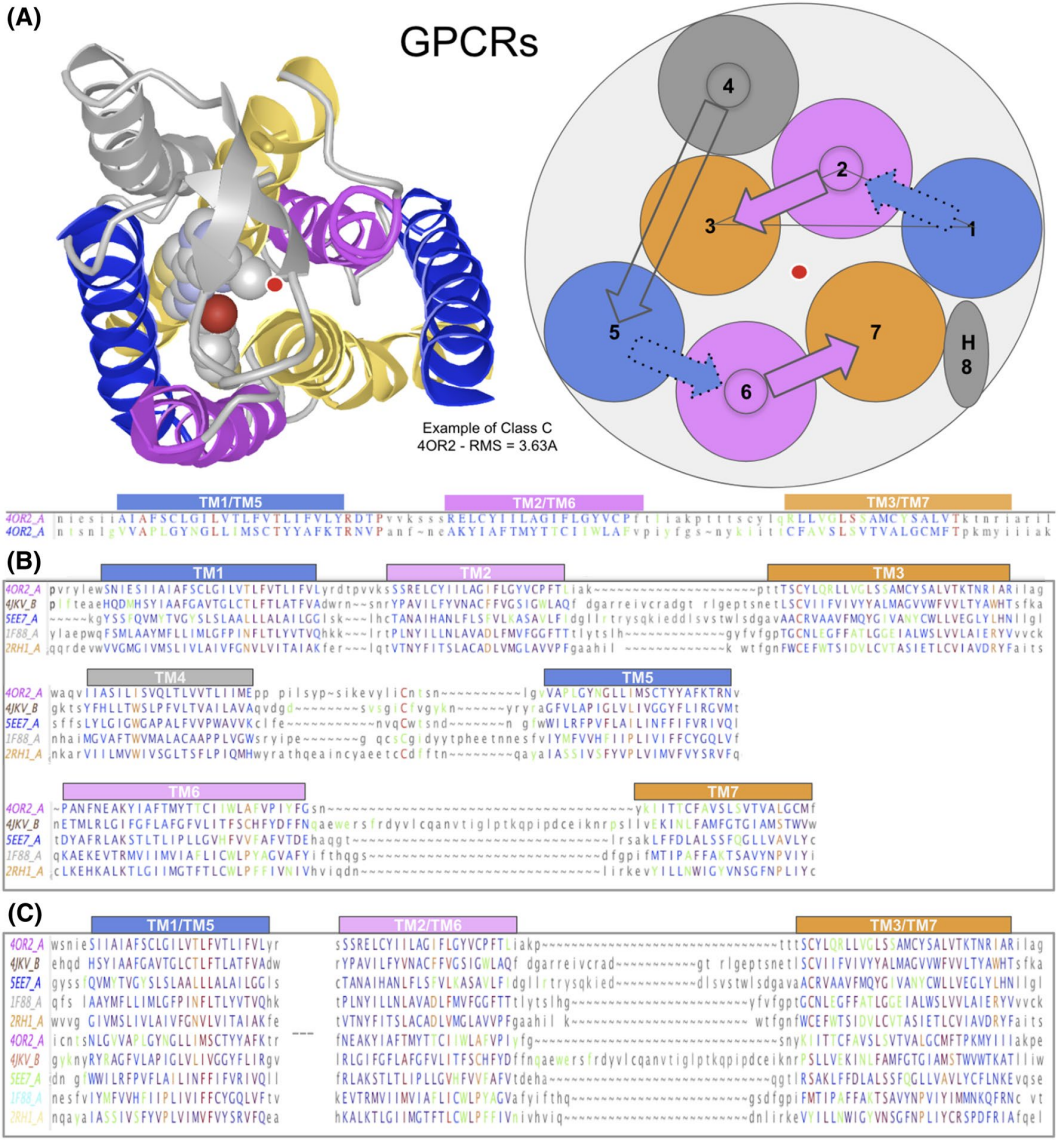
GPCR classes A, B, C, F structure-based domains and protodomains sequence alignments. **a** Structure of a GPCR with a 3D representation and a 2D representation, seen from the extracellular side. **b** Multiple domain alignment for classes C, A, C and F (RMSD relative to 4OR2.A: 4JKV.A: 3.06 Å, 5EE7.A: 2.91 Å, 1F88: 3.21 Å, 2RH1: 3.09 Å. **c** Multiple protodomains alignment: TMH-123 vs. TMH-567—RMSD relative to the first protodomain (4OR2.A-1) used as a reference structure in the alignment: 3.63 Å (4OR2.A-2), 2.12 Å (1F88.A-1), 3.29 Å (1F88.A-2), 1.73 Å (5EE7.A-1), 2.77 Å (5EE7.A-2), 1.93 Å (4JKV.A-1), 2.55 Å (4JKV.A-2), 1.79 Å (2RH1.A-1), 3.59 Å (2RH1.A-2), respectively, for class C (4OR2, human metabotropic glutamate receptor 1: GRM1), class A (1F88, bovine rhodopsin: OPSD and 2RH1, human β2 adrenergic receptor: ADRB2), class B (5EE7, human glucagon receptor: GCGR), class F (4JKV, human smoothened receptor: SMO). Similarity scale from blue least similar to red (most similar). In green: ligand proximal residues (at less than 4 Å distance) when a ligand is present in the crystal structure. In orange, the most conserved residue positions in class A (1.50 N, 2.50 D, 3.50 R, 4.50 W; not shown, 5.50 P, 6.50 P, and 7.50 P) and their counterparts in other classes

Although pseudo-symmetry had been noticed previously in Rhodopsin [(Choi et al. 2008); (Youkharibache—unpublished results)] no systematic study analyzing GPCRs’ pseudo-symmetry and corresponding protodomains alignments has been performed to date. This may well be because structural pseudo-symmetry is hard to detect computationally in GPCRs in a systematic manner with current symmetry detection programs (Kim et al. 2010; Myers-Turnbull et al. 2014). In our original census, pseudo-symmetry was detected computationally for only 18% of known GPCR domains, as GPCR protodomains are difficult to align within a very small RMSD. This is in fact a common problem in aligning helical structures, since helices tend to shift along their helix axis and move sideways (see Methods). In addition, the second protodomain (TM567) in GPCRs of class A contains proline residues in each TMH that cause kinks in those helices, increasing the RMSD in aligning to the first protodomain (TM123). This translates into difficulties to accurately delineate structural protodomains. In the case of Rhodopsin (pdbid: 1F88) (Li et al. 2004) and some other class A GPCR structures (18%), we get protodomains' alignments computationally. However, a careful interactive structural self-alignment of each GPCR domain individually, and in some cases between multiple GPCRs, although tedious, leads to a solid observation of pseudo-symmetry across all vertebrate GPCR classes (A, B, C, F), as summarized in Fig. 3 (also see Figure S6 for close-up view of GPCR protodomains).

**Fig. 4:**
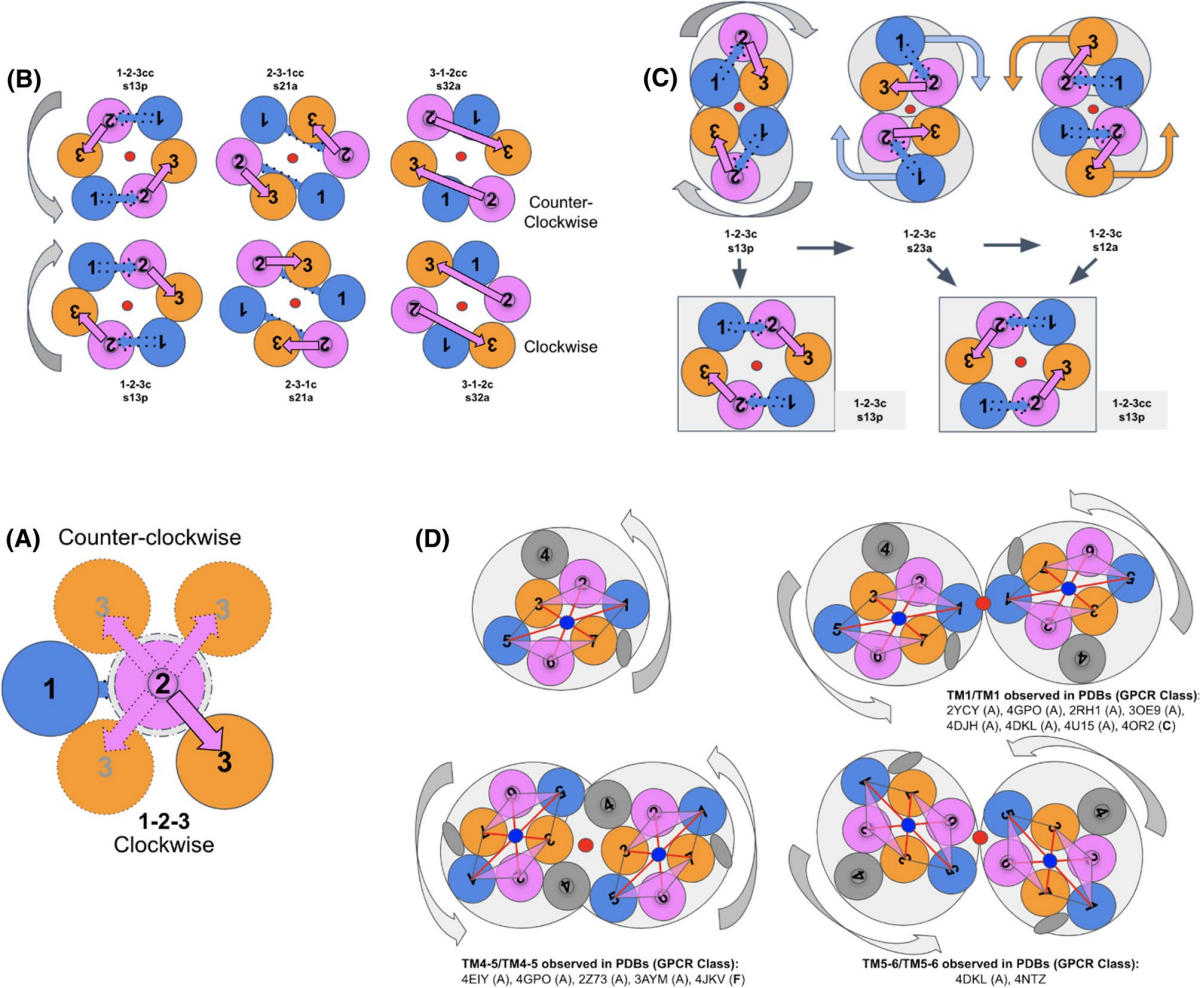
Self-Complementarity: **a** 3TMH sampling clockwise and counterclockwise topologies/conformations. **b** Duplication and symmetric assembly of self-complementary conformations [rigid or conformational selection model] of 3TMH protodomains with a 123 clockwise vs. a 123 counterclockwise topology, to form a 7TMH parallel membrane topology assuming a middle TMH linker (TM4) [not displayed for clarity) (see text). **c** Duplication and local symmetric assembly of 3TMH protodomains in the case of a 123 clockwise topology [concerted/induced conformational model] (see text). **d** Rotating 7TMH GPCR dimer sampling various symmetric homodimers observed in crystal structures (see paragraph on oligomerization of GPCRs)

GPCR structures exhibit both tertiary and quaternary levels of symmetry. This is the case, in particular, for metabotropic glutamate receptors 1 and 5 (pdbids 4OR2/5CGD). Class C GPCRs have been shown to be the most ancient GPCR class through phylogenetic analysis (Cvicek et al. 2016; Krishnan et al. 2012) and form obligatory homodimers to perform their function. Figures 1 and 4d show the two levels of symmetry that can be detected computationally. In the following discussion, we use metabotropic glutamate receptor 1 (pdbid: 4OR2) as a structural reference to analyze GPCR structures from all classes.

It is important to note upfront that structure-based protodomain alignment does not show a high sequence conservation to propose a conclusive duplication–fusion origin in the case of GPCRs of any class, as the sequence identity ranges between 10 and 19% (see Table S3 and Fig. S3). GPCRs pose a challenging problem from an evolutionary standpoint, but regardless of the evolutionary path leading to the 7TMH GPCR fold, their geometrical arrangement, i.e., the spatial arrangement of its 7 helices is exhibiting C2 symmetry, and can be considered to be formed by two 3TMH protodomains (TM123 and TM567), with a TM4 “linker” (Fig. 3a), as in SWEET proteins. RMSD-based structural homology between the two protodomains in GPCRs lies in ~ 2.4 to 3.4 Å range (Fig. 3 and Table S3), which is well within the range of clear structural homology in helical proteins. As new structures are coming out at an increasing pace (Ghosh et al. 2015; Thal et al. 2018), the protodomain idiosyncrasies in the various GPCR classes can be analyzed, in particular, GPCR class A and class C, and connected with those in bacterial 7TMH proteins, that may be related (see Table S3 and Fig. S3.C).

Figure 3 summarizes multiple structure-based sequence alignment at the domain level (Fig. 3b) and at the protodomain level (Fig. 3c), across known structure representatives of all classes of vertebrate GPCRs: A,B, C, and F (Fredriksson et al. 2003; Lagerstrom and Schioth 2008). Pairwise structure-based protodomain alignments, where sequence matching patterns are easier to see, are available as Fig. S3 for an extended set of structures.

In this section, we have shown evidence of the structural conservation of 3/4TMH protodomains within a wider set of 6/7/8TMH pseudo-symmetric protein families to identify some generalizable evolutionary patterns (Fig. 1). **This leads us to envision a possible role of conformational plasticity in fold formation, and a structural and mechanistic framework for the evolutionary deconstruction of current pseudo-symmetrical transmembrane helical (TMH) proteins as discussed next.**

### Evolution of Tertiary and Quaternary TMH Folds Through Symmetric Assembly of Protodomains

The following sections discuss the evolution of pseudo-symmetric transmembrane helical proteins through the lens of protodomains assembly. The use of protodomains can also facilitate the discussion on their evolution, whether convergent vs divergent. The possibility of a purely convergent evolution mechanism of some pseudo-symmetric domains from a structural viewpoint, introduces the concept of “conformational evolution” that can lead two different sequences to the same fold, or conversely an original sequence to different folds. The protodomain deconstruction of GPCRs leads to a hypothesis on their ancient evolutionary history. Interestingly, all the 6/7/8TMH pseudo-symmetric domains in this study also assemble as symmetric oligomers in the membrane, emphasizing the role of symmetry in evolution, revealing self-assembly and co-evolution at the domain level and at the protodomain level. In that respect, GPCRs represent an exquisite example where symmetric assembly of domains has recently been observed in dynamic rotating homo-oligomers, as will be described below.

#### Protodomains Idiosyncrasy and Symmetric Assembly

**The question on evolution and homology** of various domains is a long-standing one. For example, there is a debate over divergent *vs*. convergent evolution of Type I opsins (Bacteriorhodopsin or Sensory Rhodopsin II) *vs*. Type II opsins (Rhodopsin, a prototypical class A GPCR). There is a weak sequence homology that has been noticed for **opsins type I** between TM123 and TM567 (called ABC and EFG in that context) (Larusso et al. 2008; Taylor and Agarwal 1993). The prevalent opinion is leaning towards a convergent evolution hypothesis. Nonetheless, some authors are strong proponents of a divergent evolution scenario (Devine et al. 2013; Larusso et al. 2008; Mackin et al. 2014; Taylor and Agarwal 1993). Their question is rightly so: “*Given two transmembrane proteins with identical folds, yet no sequence similarity, how then could we distinguish convergence from homology?”.*

It is effectively an unanswered question in molecular evolution (Doolittle 1994; Murzin 1998), a particularly acute one in the cases of pseudo-symmetric domains formed through 3TMH protodomain duplication.

Protodomains (protofolds) tend to be idiosyncratic supersecondary structures. In other words, they adopt a specific topology and conformation that is duplicated and assembles in a complementary manner in forming a symmetric domain (fold). *Triple helix bundles (THB, i.e., 3TMH protodomains) have been observed in pseudo-symmetric 7TMH domains, and as we have seen, they are all effectively different, i.e., highly idiosyncratic. These repetitive supersecondary structures, or protofolds, are different for different tertiary folds* (as seen in Fig. 1). They represent a structural signature of a pseudo-symmetric domain/fold. They do not align with each other through a rigid alignment.

An open question remains about the possibility of common “***protosequences****”* among diverse folds that may be associated with a common function (Petrey et al. 2009), as in the case of SWEET vs. PnuC (Jaehme et al. 2014, 2015, 2016).

#### Convergent vs Divergent Evolution

There are a few controversial cases of convergent vs. divergent evolution discussed in the literature among the membrane proteins we considered:

The case of SWEET vs PnuC has been envisioned through a possible protodomain conformational change of a hypothetical 3TMH ancestor “semiPnu” of PnuC vs semiSWEET (Jaehme et al. 2015). The two folds are topologically different, yet one can superimpose helices by ignoring the topology. This, with the fact that both protein families have a transport function led the authors to search for an original 3TMH protodomain common to the two families. The aim in doing so was to possibly relate their sequences and make it a case of divergent evolution. This is in principle a possibility of “conformational evolution,” as we shall see later in detail, considering sequence divergence at the protodomain level, leading to different protodomain conformations, which would each assemble pseudo-symmetrically, to lead to two domain folds of different topologies, with a common function. In the case of PnuC vs. SWEET, the sequence record, however, does not allow to trace back to a common origin.

The case of Aquaporin vs. FocA is different, as in the two superfamilies share an exact same fold, named “Aquaporin-like” in structural classifications (SCOP/ECOD). They both have a transport function. Aquaporins are involved in the transport of water, but also in the transport of numerous small solutes such as glycerol, O_2_, CO_2_, sodium ion, urea, ammonia, boron, arsenic, silicon, and others, while FocA (FNT family) is involved in the transport of formate (and nitrite) ions. We clearly see, at the family level, idiosyncratic sequence/structure patterns in matching protodomains within a domain (see alignments in Fig. S2). In the literature, this is considered a case of convergent evolution (Theobald and Miller 2010). Our protodomain structural analysis can identify a symmetrically conserved pattern in TM3/TM6 as a central G/AxxxG for both FoCA and Aquaporin, a motif symmetrically conserved in FocA as GNxxG in both protodomains. This motif is in structural contact with the reentrant helix TM2b/TM5b, which in turn is characterized by a symmetrically conserved NPA motif in both protodomains of Aquaporin (see Figs. S1 and S2). In the FocA protodomains, these positions have different sequences in two protodomains (LFT/Hxx), but are preceded, however, by a highly conserved GxE/D motif, while Aquaporins have instead (if we superimpose them) an “insertion” (Figs. S2 and S10). These motifs seem to have co-evolved and differentiated concurrently within each family independently.

While the sequence/structure patterns leave no doubt of a protodomain duplication and pseudo-symmetric assembly in forming the same fold for each family, the question remains: can we find the trace of a (divergent) evolutionary relationship between the two at the sequence level? We can envision two scenarios:

**Scenario 1**—A **parallel** buildup of FoCA and Aquaporin domains through the exact same mechanism of protodomain duplication/fusion with pseudo-symmetric assembly. If so, protodomains of FoCA and Aquaporin are themselves structural homologs. The two sequences leading to one or the other may or may not come from the same protosequence originally common to both, but there is a possibility of a **diverging sequence at the protodomain level** maintaining an ancestral protodomain structure, as much as different sequences converging to the same protodomain structure. The TM3/TM6 motif (G/AxxxG) in all protodomains of FoCA and Aquaporin can sustain the possibility of a common origin, a protosequence.

**Scenario 2**—An original buildup of the Aquaporin domain through a protodomain duplication/fusion with pseudo-symmetric assembly followed by a sequence divergence **at the domain level** giving today’s FoCA and Aquaporin sequences conserving the “Aquaporin-like” fold as we know it. Since both families show a symmetric sequence pattern, but with no common pattern whatsoever, a common origin at the domain level would require a coupled evolution in both protodomains of a given family domain, simultaneously or in some concerted manner. This scenario is difficult to imagine.

The sequence similarity within each domain argues for the pseudo-symmetric assembly of both domains. The difference in the “linker” between TM2a/2b, mirrored symmetrically in TM5a/5b in both domains, but differently, argues against the second scenario. We can rather consider the first scenario to account for the observed similarity and difference: **a divergent evolution of an ancestral protosequence**, which could have led to a FoCA *vs.* an Aquaporin sequence with a conserved protodomain structure, each then duplicating and assembling independently into a domain **maintaining the same pseudo-symmetric fold**. This is consistent with what we observe systematically: a protodomain/protofold is a signature for a pseudo-symmetric fold (highly idiosyncratic). If the two sequences share a protodomain structure, whether or not evolutionary related, then that will lead to the same pseudo-symmetric fold for the domains themselves.

What makes the FocA vs Aquaporin case so remarkable is the surprising structural homology between the two (Theobald and Miller 2010), but there is also the surprising sequence similarity at the protodomain level. An element of sequence analysis comes from attempting to build an evolutionary tree (Fig. S10.B) from the structure-based protodomain alignment (Fig. S10.A). During a divergent evolution of the two protodomains within a pseudo-symmetric domain, we may expect them to show a similar phylogenetic tree from a similar ancestral sequence if effectively they would have evolved together in the same domain. We note in the tree (Fig. S10.B) that all second protodomains show the same relative phylogenetic pattern to each other as the first protodomains. On face value, that may mean that going from Aquaporins to FocA (5DYE to 4FC4 on our example extremes), the two protodomains evolved together within the same gene, for all genes concerned. In other words, the initial formation of a gene sequence containing an internal duplication would have duplicated overall and diverged as Aquaporins and FocA. However, we cannot reconcile this with a symmetric sequence motif conservation in each family, but different in the two families (see discussion above on our scenarios and Fig. S10.C). We therefore propose a possible divergent evolution of a protosequence/protofold followed by a parallel duplication/fusion with pseudo-symmetric assembly of protodomains for each family (Figure S10.C). The sequence similarity we can observe between today’s protodomains across superfamilies (in TM3/TM6), makes the common origin of a protosequence a real possibility (see Figs. S10.A and S10.D).

#### Plasticity of Protodomains and Fold Formation: Envisioning a “Conformational Evolution” Mechanism

Conformational plasticity of protodomains (protofolds) in the pseudo-symmetric assembly of domains (folds) may allow us to hypothesize a possible conformational evolution mechanism.

When we consider pseudo-symmetric folds, each one of them is formed by distinct, idiosyncratic protodomains that exhibit a particular topology and conformation. This is especially true of all the 3TMH protodomains in this analysis.

Considering discussions in the literature that relate SWEET, PnuC (Jaehme et al. 2015), FocA vs. Aquaporin (Theobald and Miller 2010), and even GPCR folds (see above) despite having significant differences in sequence, topology and conformation at the protodomain level, we feel compelled to reflect on a possible mechanism that may relate, at a minimum their topologies/conformations. We first ask the question:

1. Is there a mechanism by which various conformational changes within protodomains *(conformational divergence)* **enable the formation of structurally different folds, while being, potentially, sequence homologs**?

2. Conversely, can we envision a “homologous fold formation mechanism” that can transcend questions of fold convergence vs. sequence homology we alluded to earlier? So, is there a mechanism by which *conformational convergence* of unrelated sequences in protodomains (protofolds) followed by duplication–fusion **enable the formation of structurally similar folds**, **while not being sequence homologs originally**?

This leads us to address the structural relation between symmetric folds of different topologies, formed by different structural protodomains; folds that could eventually be considered topological isomers, for which we may have a conformational evolution mechanism. We therefore consider a conceptual mechanism that we will refer to as “**conformational evolution of 3TMH protodomains**” that would enable:

***- The creation of structurally different folds, even from related sequences****.* This would potentially address the case relating PnuC and SWEET examined earlier.

***- The formation of structurally similar pseudo-symmetric 7TMH folds from unrelated sequences.*** This would address the case of a possible convergent evolution scenario as proposed in the literature about Aquaporin vs FocA (Theobald and Miller 2010), where no similarity seems decisively showing a divergent evolution scenario. However, a divergent evolution of a protosequence is a real possibility, as reviewed in the preceding section. See Figures S2 and S10 for more details).

#### Combinatorial Sampling of 3TMH Conformations and Symmetric Self-Assembly

Protodomains composed of 3 secondary structure elements, in this case helices, bring a higher level of complexity. A 3TMH protodomain can adopt a variety of “topologies” due to the conformational plasticity of 3 connected helices (assuming helices as rigid bodies), as can be seen in Fig. 4a and the examples of Fig. 2 [see earlier discussion on topology-conformation]. Various 3TMH protodomain conformations can assemble pseudo-symmetrically as shown in Fig. 4b. One can also envision conformationally flexible 3TMH protodomains, as in Fig. 4c, that will enable pseudo-symmetric fold formation through dynamic conformational change under, for example, a structural constraint such as fusion linker (TM4) in a duplication–fusion scenario, and/or driven by binding affinities of individual helices.

As an example, for 3TMH protodomains with a **123** topology where TM2 is central (the apex of a triangle, as seen from the extracellular side), TM1 and TM3 from the two protodomains are in contact and assemble in a symmetric manner (denoted **s13p**), and so on: the **231** topology (**s21a**) and **312** topology (**s32a**). Naturally, depending on a protodomain conformation, a symmetric pairing will be parallel or antiparallel for inter-protodomains helices in contact (see Fig. 4b). We can enumerate three possible clockwise and three counterclockwise topologies, and ignoring loop connections between TMHs, any **123**, **231** or **312** order could be matched with a rotation around the symmetry axis, and similarly for the counterclockwise set. Therefore, structural similarity can be observed between folds when topology is ignored, as in the case of PnuC vs. SWEET (see earlier).

A rotating 3TMH protodomain symmetric assembly can also sample a number of conformations in an assembly process and preserve symmetry to form a 7TMH with a parallel membrane topology (assuming a middle TMH linker), in either a clockwise or counterclockwise 7TMH domain. Interestingly, two rotated 3TMH protodomains of clockwise circularity can lead to a counterclockwise domain-level topology (see Fig. 4c). Hence, either 3TMH protodomain assembly scenario, rigid or flexible, can produce the same six domain-level topologies. So, whether a protodomain symmetric assembly starts from a given stable self-complementary 3TMH conformation or involves a conformational change during an assembly process, it can reach a self-complementary symmetric assembly. Of course, this will depend on individual helices’ binding affinities, but if one helix of the first protodomain binds to a given helix of the second one, a symmetric match will occur.

#### Did GPCRs Emerge from 4TMH Proteins?

Pushing further the idea of protodomain conformational plasticity, we can envision a duplication–fusion–conformational evolution process from a 4TMH protodomain. Here we consider the formation of asymmetric assemblies of 4TMH protodomains, leading to a pseudo-symmetric 7TMH fold.

**Fig. 5:**
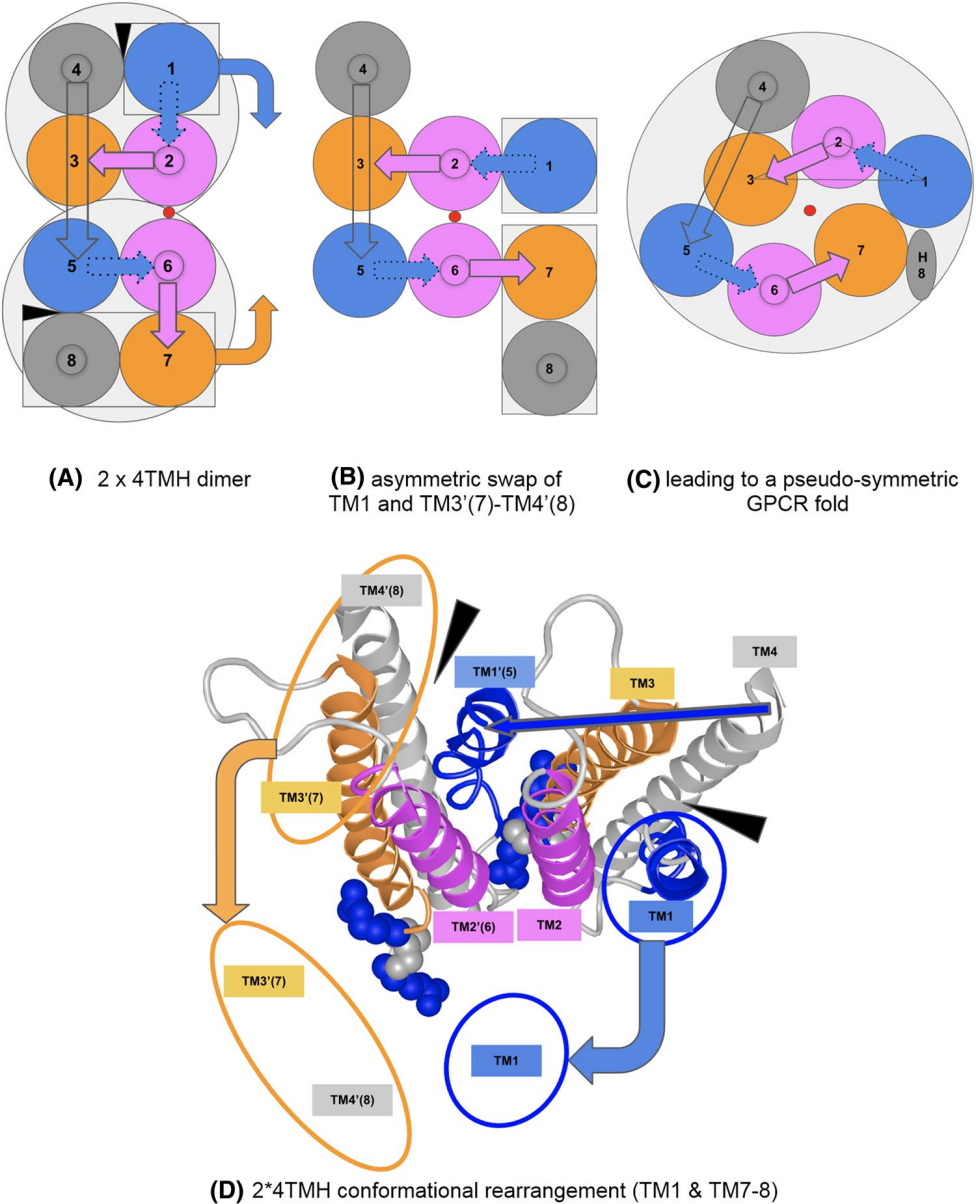
GPCR fold formation fusing two 4TMH protodomains with conformational change: Concerted asymmetric subdomain swap TM3–TM4 vs. TM1. A rearrangement scenario involving a rigid swap at the interface TM1–TM4 of both 4TMH domains forming a dimer (**a**) under a TM4(1)–TM1(2) linker constraint. This would involve a conformational change and transition from a dimer of 4TM-protein binding G-proteins to a GPCR configuration through an asymmetric swap (**b**) of one helix TM1 on one monomer vs. a two helices TM3′–TM4′ (TM7–TM8) on the second to obtain a symmetric GPCR arrangement (**c**) of TM1–2–3 vs TM5–6–7. **d** This scenario overlaid on a 3D structure of the nicotinic acetylcholine α4β2 Receptor [pdbid: 6CNK (Walsh et al. 2018)]

If we take two compact 4TMH protodomains and roll one against the other, as we did for 3TMH protodomains in Fig. 4c, keeping for example one fixed and rolling the second one against it, we can sample various dimer interfaces with symmetric or asymmetric arrangements (Fig. 5a).

In Fig. 5, we form an asymmetric dimeric interface between monomers (asymmetric contacts a23/12(= 56), like the interface between monomers in 4TMH pentamers such as pLGIC. Assuming a duplication establishing a covalent linkage between two monomers **TM4–TM5**, one can envision a conformational rearrangement starting from an asymmetric interface to lead to a symmetric protodomains arrangement (Fig. 5). This conformational change could happen through a concerted asymmetric swap of TM1 on one monomer and TM7–TM8 (TM3–TM4) on the second, involving the same 4TMH domain interface (Fig. 5b). This conceptual conformational change model is a variant to the 3TMH duplication–fusion process envisioned in the preceding paragraph for a symmetric monomer–monomer interface TM1–TM2 (s12) (see Fig. 4c). It gives, however, a rationale for the presence of the TM4 “linker.” It is interesting to note that in this case an asymmetric assembly could lead to a symmetric domain 8TMH fold, through conformational rearrangement, where further evolution can lead to the 7TMH pseudo-symmetric fold with attrition of the last helix or its transformation to the H8 helix as in GPCRs (Fig. 5c, d).

The pentameric ligand-gated ion channels (pLGICs) are composed of 4TMH proteins. These 4TMH monomers present the interfaces of our hypothetical duplication example. Experimental structures of LGICs reveal a cavity accessible to phospholipids from the lipid bilayer between TM1 and TM4 (shown with a black wedge in Fig. 5), which provides an allosteric binding site for a variety of general anesthetic ligands (Changeux 2018; Nury et al. 2011). This is the intra-subunit interface involved in the proposed asymmetric swap in 4TMH. So, their M1–M2–M3(–M4) regions will map, upon duplication to TM1–TM2–TM3(–TM4) and TM5–TM6–TM7(–H8) protodomains of GPCRs. Specifically, the nicotinic acetylcholine receptor Achɑ7, a pLGIC with a 4TMH domain, has recently been shown (Kabbani and Nichols 2018; Kabbani et al. 2013; King and Kabbani 2016; King et al. 2015) to couple to G-proteins through an RxxR motif in its M3–M4 loop. This region maps to the RxxR motif of class C GPCRs and the DRY motif of class A GPCRs at the end of TM3, which are in the G-protein coupling regions of GPCRs. It has not escaped our notice that Achɑ7 (and other pLGICs) could be one of the potential protodomain sources for GPCRs. However, the duplication mechanism itself needs to be investigated further before an evolutionary link can be established between pLGICs and GPCRs.

#### Oligomerization in GPCRs

At the domain level, symmetric dimerization/assembly is a common pattern of GPCRs (Fig. 4d), like the rotating protodomain symmetric assembly process used in Figs. 4c or 5a. As observed in all 6/7/8TMHs analyzed in this work, they all form higher order oligomers (see Fig. 1). GPCRs are a special case, as they sample multiple homodimer interfaces. Effectively, the same principle of symmetric dimerization used in sampling symmetric interfaces between protodomains (Fig. 4c) can be applied at the domain level to explain observed dimers in GPCRs. In fact, *dynamic* rotating symmetric dimers have recently been observed in a class C GPCR (Dijkman et al. 2018; Xue et al. 2015). Figure 4d shows two rotating dimers synchronized on rotation to maintain a symmetric organization sampling of homodimers, which has been observed in multiple GPCR crystal structures.

Symmetric dimers form the majority of experimentally determined protein quaternary structures. In the PDB, among ~ 150,000 structures of macromolecular complexes, ~ 53,000 exhibit quaternary symmetry, with ~ 42,000 (78%) presenting a cyclic symmetry (Korkmaz et al. 2018). Cyclic C2 symmetry is found in ~ 32,000 structures, with ~ 31,000 being homodimers, an overwhelming majority. What makes GPCR dimers so special is their ability to form dynamic symmetric dimers of variable geometry, where dimeric states *or conformations* have been shown to be sampled during the lifetime of the dimer (Dijkman et al. 2018; Xue et al. 2015) in the plasma membrane.

#### Oligomerization in Other TMH Proteins

As observed in the set of C2 pseudo-symmetric TMH protein domains used in this study (Fig. 1), they all oligomerize to form quaternary structures with various symmetries (C2 to C5). The tertiary and quaternary symmetry axes may combine to form structures of higher symmetry, belonging to C2 to D5 symmetry point groups in the selected examples. While we have not analyzed systematically tertiary/quaternary combined symmetries over all known structures, our experience shows that pseudo-symmetric domains tend to oligomerize to form symmetric quaternary assemblies, for membrane and globular proteins alike.

## Consequences of Pseudo-Symmetric Assembly in TMH Function and Evolutionary Role of the Lipid Bilayer

### Emergence of New Functions at the Protodomain Interface

#### Symmetrically Related TM3/TM7 Ligand Binding in GPCRs

**Fig. 6:**
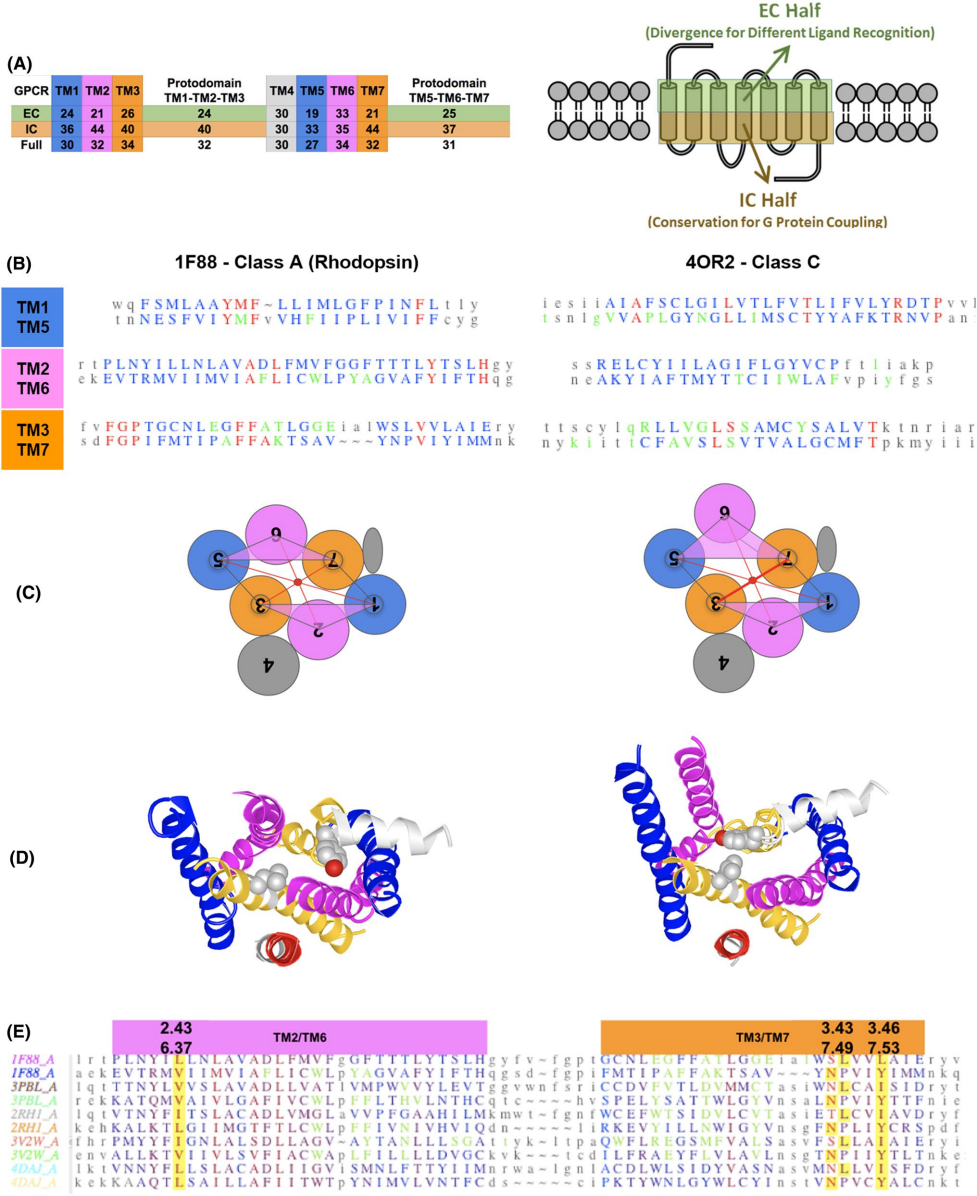
Deconstruction of GPCR Domains. **a** Right panel: Definition of TMH halves facing the Extracellular (EC) and Intracellular (IC) sides. Left Panel: Sequence similarity score (see Methods section for details) of the aligned EC half, IC half, and Full TM sequences for each of the 7 TMs for a diverse set of GPCRs spanning all classes and subclasses. Protodomain 1 and 2 scores are also given (averages over 3TMH). Colors correspond to TMHs vertically and EC and IC halves horizontally. EC halves show a lower score for each TMH compared to IC halves in GPCRs (see text). **b** Pairwise Protodomain alignment (RMSD: 1F88 = 3.24 Å; 4OR2 = 3.31 Å). The symmetrically conserved pattern, especially in TM3/TM7 surrounding the ligand, in each domain is idiosyncratically conserved (Red = conserved, Green indicates ligand binding/proximity residues (in less than 4 Å distance) (see text). **c**–**d** Rhodopsin inactive vs. active conformational change seen from the IC side (binding G-protein not shown for clarity). Left inactive (PDB: 1GZM), right active (pdbid 6CMO). The optimum alignment as shown is obtained from VAST + (Madej et al. 2014) [see Figure S12 for details). The iCn3D*visualization* (using the “alternate” command—keyboard shortcut “a”) gives a good grasp of the conformational change (see text). **e** Multiple alignment of protodomains of a number of Class A GPCRs showing symmetry-related residue pairs (highlighted in yellow) also involved in key contacts (in green) [See text for details]

Considering only the transmembrane helices, ligand binding residues can be distributed on all TMHs; however, most ligands bind effectively to the 3 helices (TM5, TM6, TM7) in protodomain 2 and TM3 in protodomain 1. This can be seen in multiple examples of class A GPCRs (green residues in Fig. 6b and Fig. S3). Hence in terms of pseudo-symmetry and ligand binding, it involves essentially the pseudo-symmetric TM3/TM7 pair, with anchor residues for ligand binding positioned symmetrically. Ligand binding residues’ positions are mostly **3.28, 3.32–3.33, 3.36–3.37 in TM3 vs. 7.35, 7.39, 7.42–7.43 in TM7** [using the Ballesteros-Weinstein numbering of TM residues for GPCRs (Visiers et al. 2002)]. TM3–TM7 appear to be the only obligatory transmembrane helix pair for ligand binding, and their two binding regions are symmetrically related.

Matching residues in TM3/TM7 are **FFA(T/K)** in the case of bovine rhodopsin, and **V(G/S)LS** in human metabotropic glutamate receptor (see Fig. 6b). In addition, in rhodopsin, TM1 shows a YMF pattern in TM1 and a A(D/F)L pattern in TM2 (see following discussion on the **D**^**2.50**^**/F**^**6.44**^ alignment). While YMF and FFA patterns of protodomain 1 are in direct contact and move in a concerted way during an inactive (1F88) to active (2X72) transition, one cannot point to a contact between these two motifs on protodomain 2. In the metabotropic glutamate receptor, the structural homology of TM1/TM5 extends beyond helices into the loop regions with an RxxP pattern (see Fig. 6b). In rhodopsin, an FGP motif can be seen before TM3/TM7. Rhodopsin is unique in that its ligand is covalently bound to the TM7 Lysine; hence this is the only case where we inserted a gap vs. TM3 to optimize the alignment.

In the second protodomain, for class A GPCRs, TM6 residue **W**^**6.48**^ is a highly noticeable ligand binding residue, along with residues **6.51–6.52** around the conserved residue **P**^**6.50**^. The residue **F6.44** (in protodomain 2) can also sometimes be involved in ligand binding. Its symmetry-related highly conserved residue **D**^**2.50**^ on TM2 (in protodomain 1) does not bind to the ligand. In fact, D2.50 binds to Na^+^ ion that has been shown to correlate with the functional state of the receptors (White et al. 2018). It can be symmetrically paired with either **F**^**6.44**^** or W**^**6.48**^, both highly conserved, and separated by a helix turn. This points to a **co-evolution ofD**^**2.50**^**with F**^**6.44**^** and/or W**^**6.48**^**.** In fact, in pairwise protodomain alignments (see **Figure S3.A**), we have alternative protodomains alignments where **D**^**2.50**^ can be equivalenced to either **W**^**6.48**^ or **F**^**6.44**^. It is a common feature in helical proteins alignments to see translations of helices along their helical axis, shifting residues in positions ± 4 (see note in section Methods—Protodomains delineation). This “conformational translation” of the TM6 helix may possibly have a functional significance in allowing an up–down movement along the helix axis.

#### G-Protein Binding and the TM3/TM7 Paired Interactions

Additional functional significance emerges around the TM3/TM7 paired interactions. Just below the ligand binding area, the highly conserved class A GPCR residues **S**^**3.39**^** and N/S**^**7.45**^** (or S**^**7.46**^**)** match symmetrically across the protodomains. They are Na^+^-binding residues (White et al. 2018). Below the Na^+^-binding area, when one looks at helix–helix contacts that change upon activation (Cvicek et al. 2016; Venkatakrishnan et al. 2016), TM3 and TM7 form contacts between residues at positions **3.43–7.49, 3.43–7.53, 3.46–7.53** in the active conformations but not in the inactive conformation.

The two regions in TM3 and TM7 match pseudo-symmetrically whether in the active or inactive state, yet they form direct contacts in the active state. In Fig. 6c, d, we show the case of rhodopsin, seen from the intracellular side where two Leucine residues in position **3.43 and 3.46** (and similar I/L/M residues in other class A GPCRs) form contacts with the highly conserved Tyrosine **7.53** (see protodomain alignments and highlights in Fig. 6e). Interestingly both protodomains, despite the conformational change in active and inactive states, align within a 3 Å RMSD (see Fig. 6c–e, and Fig. S3.A for detailed pairwise protodomain alignments). The conformational rearrangement is distributed on many residues and degrees of freedom. The TM3–TM7 helix pair plays a central role in coupling and maintaining symmetry, and bringing the regions 7.49–7.53 with 3.43–3.46 in contact, with a noticeable change in Y^7.53^ conformation and orientation.

These observations show that the extracellular-facing halves of TM3/TM7 provide endogenous ligand contacts along with Na^+^-mediated contacts, and their intracellular-facing halves provide direct contact with each other upon activation. **This places the TM3/TM7 interface at the heart of GPCR functional action**. Previous studies (Venkatakrishnan et al. 2013) have made the case for the central role of TM3 in GPCR function; however, our protodomain hypothesis suggests that TM3 and its pseudo-symmetric partner TM7, together, play a “pivotal” role in that function.

#### Each Pseudo-Symmetric Protein Shows a Unique Protodomain Co-Evolution Pattern

Each GPCR domain has its own evolutionary history; however, each maintains an internal homology and an identity pattern for some symmetrically equivalent residues. Such identity patterns are idiosyncratic, and they are different between various GPCRs. We observe such symmetric sequence pattern “coincidences,” or identities, in several GPCR protodomain pairwise alignments (Fig. S3).

The diversity and complexity of GPCRs is such that it is extremely difficult to infer co-evolution patterns between protodomains through a simple observation of a sequence alignment. While the **D**^**2.50**^**/F**^**6.44**^**xxxW**^**6.48**^ pattern is relatively easy to pick (see earlier discussion), there are certainly other co-evolved pairs (or larger sets of residues) in GPCRs to detect, as we usually find in pseudo-symmetric domains (Youkharibache 2019). Another pair of residues/motifs that are related in function are the symmetrically related pair in TM3 and TM7: **S**^**3.39**^**/N**^**7.45**^**S**^**7.46**^. For both of these pairs (TM2/6 pair and TM3/7 pair), we performed a statistical analysis of all available GPCR sequences using the GPCR-SAS server (Gomez Tamayo et al. 2018) as an **odds ratio** of having one residue/motif in one position and another residue/motif in the second position: an odds ratio of 2 means that if a specific residue/motif is present at the first position, and then it is twice as likely to find another specific residue/motif at the second position. The results are presented in Table S4. For the structurally and functionally linked positions across the TM3/TM7 interface of **conserved S**^**3.39**^** residue in TM3 *vs.* N**^**7.45**^**S**^**7.46**^** motif in TM7**, the odds ratio is 76.8 in humans (29.0 in mammals and 52.4 in vertebrates) for GPCR subclass Aα. For the GPCR subclass Aβ, this odds ratio is 12.3 in humans (21.3 in mammals and 16.9 in vertebrates). For an example of structurally and functionally linked motifs across TM2/TM6 interface of **D**^**2.50**^ on **TM2** and **F**^**6.44**^**xxxW**^**6.48**^ on **TM6**, the odds ratio is 24.6 in humans (9.6 in mammals and 3.1 in vertebrates) for the GPCR subclass Aα. The data show that these residue/motif positions are highly correlated evolutionarily in the class A GPCRs that dominates the GPCR superfamily. The positions of these motifs in the protodomain topology of GPCRs provides a structural context to their functional importance and co-evolution. Systematic studies would be needed to provide evidence of this context and can now be envisioned to identify co-evolution patterns, between protodomains, especially in the case of GPCRs with ligands. These relationships are certainly very complex, but a pseudo-symmetric decomposition of domains into protodomains can begin to provide testable hypotheses.

### Evolution of Ligand Binding (EC) Region vs G-Protein Binding (IC) Region in GPCRs

The TMH proteins cover a very wide range of functions due to their prime location at the cellular surface, which enables them to be utilized for jobs like transport of molecules in/out of the cell and sensing of extracellular signals to trigger intracellular responses. The cell membrane’s lipid bilayer environment is inherently asymmetric, where the outer lipid leaflet faces the extracellular (EC) side and the inner lipid leaflet faces the cytoplasm on the intracellular (IC) side. This asymmetry adds a natural directionality to their transporter and receptor functions. The TMH proteins embedded in this asymmetric environment can potentially feel different evolutionary constraints on their EC-facing and IC-facing TMH halves, which can leave a distinct function-based evolutionary signature in these protein halves.

To identify these potential evolutionary signatures in each of the TMH protein families being analyzed, a diverse set of proteins were identified in each studied TMH family along with one or more experimental structures available in each family oriented in the membrane by the OPM database (Lomize et al. 2012). The list of proteins and PDB ids used for each family is provided in the Supplement File SF1. The sequences of proteins in each family are aligned to each other using MAFFT (Katoh and Standley 2014) and the EC/IC-facing halves of TM regions identified for each protein in the set by utilizing the membrane orientation of the reference structure in each family (see Methods section for details). The corresponding alignments for all TM regions in each protein family are shown in Supplement File SF2 (*h* corresponds to the hydrophobic center for each TM in the OPM oriented configuration). The EC and IC loops as well as N and C termini of all proteins are ignored as they are usually of highly variable lengths are difficult to align correctly. Skipping these loop regions from the analysis is reasonable as we have shown previously (Cvicek et al. 2016) through a TM-region-only alignment of all human GPCRs that the TM regions contain enough evolutionary information to enable an accurate phylogenetic representation of different GPCR families. The sequence similarity of the EC-facing and IC-facing TM halves were calculated for each TMH protein family to look for differences in evolutionary divergence of these EC and IC-facing halves.

Structures of 35 diverse GPCRs were aligned to each other utilizing the TM regions as mentioned above, which provided a corresponding (potentially more accurate) sequence alignment. This sequence alignment was used to compare the IC-facing half (G-protein coupling side) vs EC-facing half (ligand binding side). Figure 6a shows the sequence similarity across a diverse set of 35 GPCRs to assess the extent of divergence in each TM, in each of the two protodomains, and in each half (EC-facing and IC-facing) of the TM domains.

The sequence similarities showed that EC-facing half of TM regions in GPCRs has evolved more than the IC-facing half of TM regions for all seven TMs, consistent with the fact that GPCRs sense a huge chemically diverse set of ligands using their EC-facing half, but they couple to only a small family of G-proteins using the IC-facing half. The sequence similarities also show that TMs 5, 6, and 7 (protodomain 2) have evolved to the same extent as the TMs 1, 2, and 3 (protodomain 1) (31% vs 32%, respectively, as seen in Fig. 6a).

These results show that functionally GPCRs live in a highly asymmetric environment due to G-protein coupling on one side and ligand binding on the other side, which is captured in higher sequence similarity in the IC region for both protodomains (40% and 37%, respectively, vs 24% and 25% in the EC region).

### Comparing EC vs IC Regions for Other TMH Proteins

Figure S11 shows the sequence similarity of the EC-facing and IC-facing TM halves of selected other TMH proteins: Aquaporins, Foca, PnuC, TRIC, and MFS. Some of the features that emerge are as follows. The two protodomains in each of these families have diverged to the same extent like GPCRs, except for TRIC and MFS where the second protodomain (TRIC) or the last two protodomains (MFS) have diverged more. The origin of this difference in protodomain divergence is not clear and requires detailed analysis of available structures and residue co-evolution patterns.

Aquaporins have the NPA motif in TMs 2B and 5B that impart high conservation to those segments. FocA transports formate molecule bidirectionally; however, it shows the GPCR-like pattern of its IC-facing half being more conserved than the EC-facing half. This is likely due to FocA interacting with its cytoplasmic partners like Pyruvate formate-lyase (Doberenz et al. 2014) and 2-ketobutyrate formate-lyase (TdcE) (Falke et al. 2016) for its function, putting evolutionary pressure on the IC half to be more conserved than the EC half. PnuC has a conserved WxxW in the IC half of TM6 that binds to its substrate; hence it is more conserved than other TMs. TRIC has a conserved GG motif in TMs 2/5, and since it is an ion conduction channel it contains conserved residues along the whole TM length, so no EC vs IC patterns emerge like in GPCRs.

In this section, we have seen the functional significance of protodomain assemblies of TMH proteins and the role played by the asymmetric membrane environment in their evolution.

## Conclusion

In this study, we have established a parallel between diverse 6/7/8TMH protein families that shows a similar evolutionary path of duplication–fusion and symmetric assembly of 3/4TMH protodomains. The “homology” we demonstrate does not reside in a common structural fold but rather in the common pseudo-symmetric assembly mechanism during evolution that leads to diverse structural folds. The parallel evolutionary path does not necessarily imply that these proteins have a common origin in sequence space. What stands out however among 6/7/8TMH proteins is the formation of a diverse set of folds from conformationally variable 3/4TMH protofolds. This should also be put in perspective with a significant overrepresentation of 7TMH proteins in the surfaceome (Bausch-Fluck et al. 2018).

A reason for the evolutionary success of 7TMH proteins may well be a structural one. The creation of an almost cylindrical unit provides a molecular device with a natural directionality to channel/transport molecules or ions across a membrane, or for transmembrane signaling. Duplication and symmetric assembly of a 3/4TMH around an axis normal to the cell membrane looks like a simple mechanism to get to the minimum size cylinder with a directional function. In addition, 3/4TMH protodomains provide cohesive energetically stable supersecondary structural units that can self-assemble. The biophysical evidence is only now beginning to emerge (Min et al. 2018).

In the examples selected, some folds may share a function, such as transport across the membrane, yet they may not have evolved from the same ancestor, as in the case of SWEET vs PnuC. Conversely, functional diversification may have been obtained from common protogene/protodomain ancestor, as in the case of FocA vs. Aquaporin, where one can explain the convergence of these two domains by a parallel duplication/fusion/symmetric assembly process of 3TMH protodomains homologs.

We have a general molecular self-assembly principle at work, in membrane and globular proteins alike, forming pseudo-symmetric tertiary structures (domains) that may assemble themselves to form symmetric oligomers. This is the case for all TMH proteins reviewed in this paper. The coincidence between symmetry axes in helical membrane proteins and a lipid membrane axis system, considering many of their functions, tends to imply that a large number of membrane proteins should be symmetric. GPCRs, whose function does not seem to require symmetry, nevertheless exhibit pseudo-symmetry, where the second protodomain (TM5-TM6-TM7) undergoes conformational change upon receptor activation to accommodate the G-protein.

We have reviewed the parallel evolution of a variety of 3/4TMH protodomains that lead to a number of 6/7/8TMH proteins and provide a framework to interrogate their evolutionary origins. We introduced a concept of *conformational evolution* that can, in principle, shed some light on convergent vs. divergent evolution of pseudo-symmetric domains. This work provides a protodomain assembly framework to deconstruct pseudo-symmetric proteins and to provide testable hypotheses for understanding the mechanism(s) of protodomain assembly and membrane protein folding. This study also highlights a need for a more systematic study of co-evolution of protodomains, especially in GPCRs. This should be possible as the number of membrane proteins structures are now growing at the same exponential pace as globular proteins (https://www.rcsb.org/stats/growth/overall), with GPCRs leading the charge.

## Electronic supplementary material

Below is the link to the electronic supplementary material.

Supplementary Information (PDF 5708 kb)

Supplementary file SF1 (PDF 2278 kb)

Supplementary file SF2 (PDF 99 kb)
