# Supplementary material for: Pseudo-Symmetric Assembly of Protodomains as a Common Denominator in the Evolution of Polytopic Helical Membrane Proteins
Source: J Mol Evol. 2020 Mar 18;88(4):319–44. doi: 10.1007/s00239-020-09934-4 (PMC7162841; doi:10.1007/s00239-020-09934-4)
Supplement: Supplementary file 1 — Supplementary Information (PDF 5708 kb) [file 239_2020_9934_MOESM1_ESM.pdf]

Pseudo-Symmetric Assembly of Protodomains as a Common Denominator in the Evolution of Polytopic Helical Membrane Proteins

Philippe Youkharibache[1,*], Alexander Tran[2], and Ravinder Abrol[2,*]

[1] Cancer Data Science Lab, Center for Cancer Research, National Cancer Institute, National Institutes of Health, Bethesda, MD, USA

[2] Department of Chemistry and Biochemistry, California State University, Northridge, CA, USA

SUPPLEMENTARY INFORMATION

MFS	5EQI	https://d55qc.app.goo.gl/wcE7nXES2yhE4CQr6
TRIC	5WUF<br>3KCU	https://d55qc.app.goo.gl/XUkPY7iPmR8LVJ8c9<br>https://d55qc.app.goo.gl/srgkH59q3ADhRSTS8
AQP1	3NE2	https://d55qc.app.goo.gl/pN9hZKMbiS4VML5H6
SemiSWEET Apo vs. Ligand	4QNC<br>4QND	https://d55qc.app.goo.gl/i9vWJfzXEcL8q2A69
SWEET vs. SemiSWEET	5CTH<br>4QND	https://d55qc.app.goo.gl/9NfEK6KmideLHYt1A<br>Use keyboard “a” letter or “Alternate” command to visualize alternatively the 2 aligned structures of SWEET (PDB:5CTH) vs. 3TMH monomer A of SemiSWEET (PDB:4QND) (aligned only on 3TMH protodomain 1)<br>Use “a” alternate command to alternate between structures
PnuC	4QTN	https://d55qc.app.goo.gl/S1atQgdGptdt7bCs6
GPCR C	4OR2	https://d55qc.app.goo.gl/ZFChMCsBRER3uSbD7
GPCR Aα	5G53	https://d55qc.app.goo.gl/igoFR7sh9hZdjK1L9
Rhodopsin	1GZM	https://d55qc.app.goo.gl/KtAe6nkkSJ7fm3aHA
Rhodopsin active-inactive	6CMO<br>1GZM	https://d55qc.app.goo.gl/KLJg7G6Zx3g9hju4A
pLGIC AcetylCholine Receptor	6CNK	https://icn3d.page.link/Knzy

Table S1 - 3D visualization links - using iCn3D

	TM1	TM2	2b	TM3	3TMH	RMS (A)
sequence length aligned (5CTH)	20	18		25	63	
**SWEET 123 567 (# res. identical)**	3	5		5	13	
**%Id**	**15%**	**28%**		**20%**	**21%**	**1.36**
**vs. SemiSWEET** (4QNC/4QND)	0/6(*)	3		4	7/13	
**%Id**	**0/30%**	**17%**		**16%**	**11/21%**	**1.98**
length aligned (4QTN)	13	42		23	78	
**PnuC 123 vs 567**	1	6		4	11	
**%Id**	**8%**	**14%**		**17%**	**14%**	**1.26**
length aligned (5WUF)	23	23		16	62	
**TriC 123 vs 456**	8	10		4	22	
**%Id**	**35%**	**43%**		**25%**	**35%**	**1.53**
length aligned (4FC4)	21	18	13	23	75	
**FocA 123 vs 456**	4	3	1	8	16	
**%Id**	**19%**	**17%**	**8%**	**35%**	**21%**	**1.37**
length aligned (5DYE)	24	25	10	19	78	
**Aquaporin 123 vs 456**	4	5	4	4	17	
**%Id**	**17%**	**20%**	**40%**	**21%**	**22%**	**1.92**
length aligned (3C02)	24	25	14	19	82	
**Aquaglyceroporin 123 vs 456**	5	5	3	4	17	
**%Id**	**21%**	**20%**	**21%**	**21%**	**21%**	**1.92**
length aligned (4FC4-1 / 5DYE-2)	21	17	12	23	73	
**FocA vs. Aquaporin**	3	4	0	8	15	
**%Id**	**14%**	**24%**	**0%**	**35%**	**21%**	**2.94**
length aligned (5EQI)	18	18		18	54	
**MFS 123 vs 456 (protos1-2)**	4	3		2	9	
**%Id**	**22%**	**17%**		**11%**	**17%**	**1.70**
**vs 789 (protos1-3)**	2/4(**)	1/6(**)		4		
**%Id**	**11/22%(**)**	**6/33%(**)**		**22%**	**13/26%**	**1.88/2.97(**)**
**vs 10-11-12 (protos1-4)**	3	1		2		
**%Id**	**17%**	**6%**		**11%**	**11%**	**2.32**
**Average**	**19%**	**21%**	**17%**	**22%**	**20%**	**1.81**
Min	8%	6%	0%	11%	11%	1.26
Max	35%	43%	40%	35%	35%	2.94

Table S2 - Protodomain alignment statistics for SWEET, PnuC, TriC, FoCA, Aquaporin, and MFS

(*) a shift of 3 in sequence will match SPLA sequence in SWEET proto2, part of the FxSP motif in TM1/5 conserved in SWEET protos

(**) A 4 protodomains optimized multiple structure alignment. A shift in sequence vs optimized structure alignment would for example increase the sequence match from 1 to 6 in TM2 for an overall RMS change from 1.88 Å to 2.97 Å

GPCRs	TM1/5	TM2/6		TM3/7	3TMH	RMS (A)	Symmetry	TM1/4	TM2/5		TM3/6	3TMH	RMS (A)	Symmetry
length aligned	21	29		27	77			21	29		27			
1F88 123 vs 567 (# res identical)	4	4		7	15		Rhodopsin	2	4		2			
%Id	**19%**	**14%**		**26%**	**19%**	**3.24**	SYM	**10%**	**14%**		**7%**	**10%**	**7.73**	NO SYM
length aligned	28	21		21	70			28	21		21			
4OR2 123 vs 567	5	0		4	9		Class C	3	1		0			
%Id	**18%**	**0%**		**19%**	**13%**	**3.36**	SYM	**11%**	**5%**		**0%**	**5%**	**4.89**	NO SYM
length aligned	22	21		24	67			22	21		24			
4MBS 123 vs 567	6	0		3	9		Class A	3	4		1			
%Id	**27%**	**0%**		**13%**	**13%**	**2.62**	SYM	**14%**	**19%**		**4%**	**12%**	**6.78**	NO SYM
length aligned	28	29		28	85									
4GRV 123 vs 567	6	2		7	15		Class A							
%Id	**21%**	**7%**		**25%**	**18%**	**3.31**	SYM							
length aligned	22	25		20	67									
3RZE 123 vs 567	4	0		4	8		Class A							
%Id	**18%**	**0%**		**20%**	**12%**	**3.38**	SYM							
length aligned	24	24		18	66									
3OE9 123 vs 567	5	3		1	9		Class A							
%Id	**21%**	**13%**		**6%**	**14%**	**3.38**	SYM							
length aligned	24	26		20	70			16	20		20	56		
4JKV 123 vs 567	6	2		2	10		Class F	2	3		3	8		
%Id	**25%**	**8%**		**10%**	**14%**	**2.39**	SYM	**13%**	**15%**		**15%**	**14%**	**5.23**	NO SYM
length aligned	25	22		22	69			16	28		21	65		
5EE7 123 vs 567	2	2		3	7		Class B	1	3		1	5		
%Id	**8%**	**9%**		**14%**	**10%**	**2.56**	SYM	**6%**	**11%**		**5%**	**7%**	**6.49**	NO SYM
length aligned	23	16		25	64									
4EIY 123 vs 567	1	2		4	7		Na+ binding							
%Id	**4%**	**13%**		**16%**	**11%**	**2.68**	SYM							
length aligned	17	24		17	58									
1UAZ ABC vs EFG	3	6		0	9		Bacteriorhodopsin							
%Id	**18%**	**25%**		**0%**	**16%**	**2.56**	SYM							
Average (GPCRs)	**18%**	**7%**		**16%**	**14%**	**2.99**		**11%**	**13%**		**4%**	**9%**	**6.47**	
Min	4%	0%		6%	10%	2.39	SYM	10%	5%	0%	0%	5%	4.89	NO SYM
Max	27%	14%		26%	19%	3.38		14%	19%	0%	7%	12%	7.73	

Table S3 - Protodomain alignment statistics for GPCRs (and Bacteriorhodopsin). **[Left]** Sequence Identity and RMSD of the two 3TMH **TM123/TM567** protodomains matching symmetrically and individual matching transmembrane helices. **[Right]** Protodomain 1 **TM123 vs 3TMH TM456** for comparison (not related symmetrically).

Odds Ratio	Motif1: S3.39<br>Motif2: NS7.46		Motif1: D2.50<br>Motif2: FxxxW6.48	
GPCR-Class	Aα	Aγ	Aα	Aγ
Humans	76.8	12.3	24.6	-
Mammals	29.0	21.3	9.6	2.6
Vertebrates	52.4	16.9	3.1	2.6

Table S4: Odds-Ratio of three pairs of co-evolutionary related residues/motifs, calculated using the GPCR-SAS server [http://lmc.uab.cat/gpcrsas/, Tamayo et al. (**2018**) *PLoS One*, **13**(7):e0199843] for available GPCRs from humans, mammals, and vertebrates. Numbers following the motifs are the BW numbers of the only residue or last residue in the motif.

Pairwise protodomain alignments

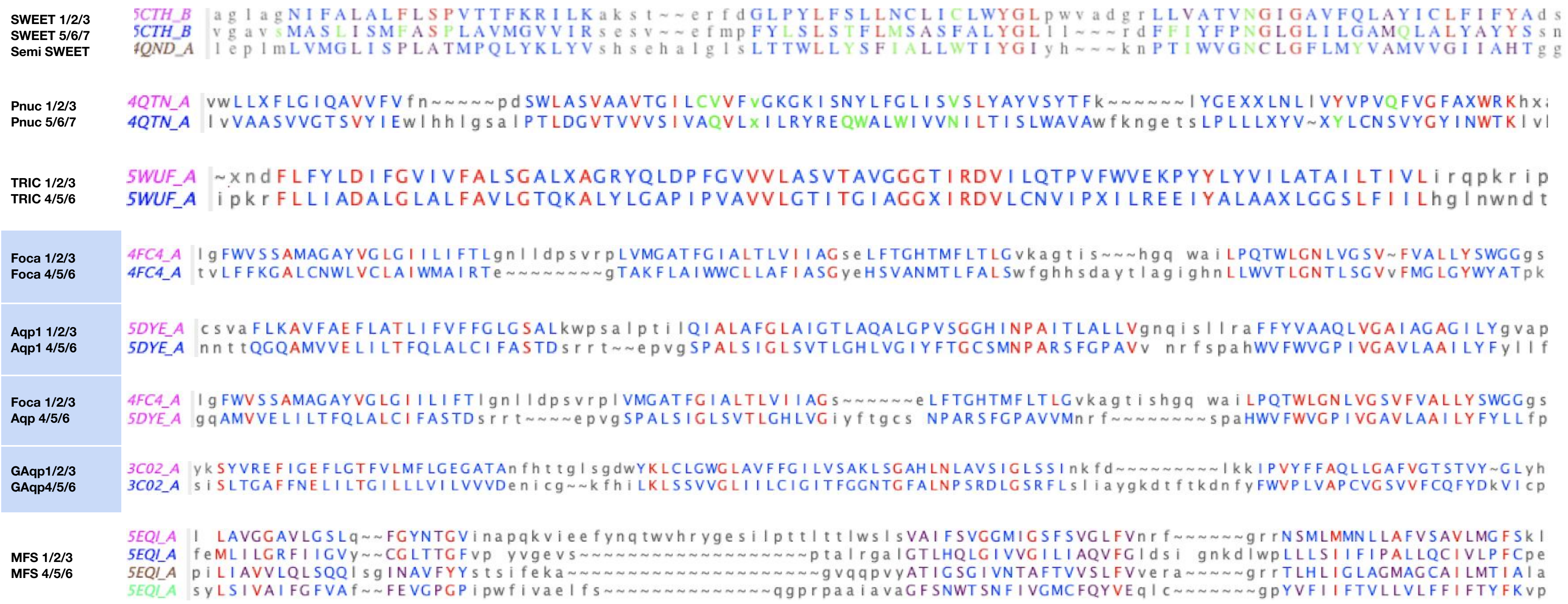

Figure S1 - Pairwise alignment of protodomains of: SWEET protodomains 1.36 Å RMS / 21 % Id (5CTH) vs. **SemiSWEET** 1.98 Å RMS / 16% Id (4QND), **PnuC**: 1.26 Å RMS / 13% Id (4QTN), **TriC**: 1.53 Å RMS / 34% Id (5WUF), **FocA**: 1.83 Å RMS / 20% Id (4FC4), **Aqp1**: 1.36 Å RMS / 24% Id (5DYE), An example of surprising sequence/structure homology between **FocA (4FC4) protodomain 1** vs. **Aqp (5DYE) protodomain 2**: 2.94 Å RMS / 34% Id and 18 %Identity . **MFS** 1.70 Å RMS / 17% Id on first 2 protodomains and 1.70 Å RMS / 17% Id on protodomains 3&4 (5EQI) - after optimisation of protodomain boundaries for best structural match.

Protodomain alignments (pairwise, familywise and across two families FocA and Aquaporin)

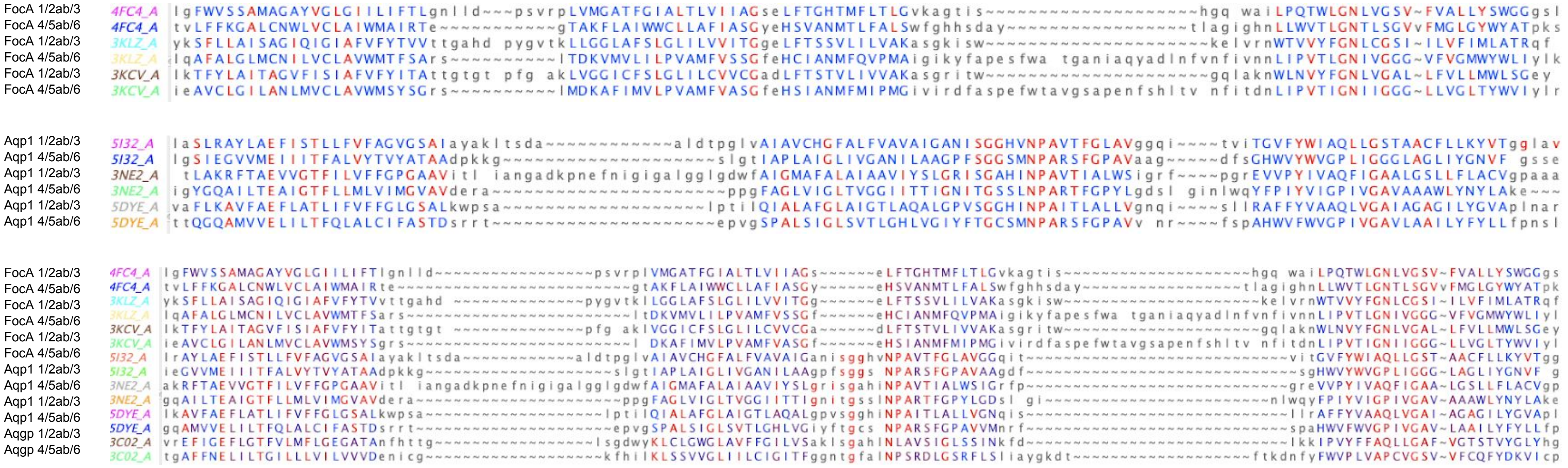

Figure S2 - Multiple alignment of FocA and Aquaporin protodomains. FocA (PDB: 4FC4, 3KLZ, 3KCV), **AQP** (PDB: 3NE2, 5I32, 5DYE, 3C02)

Protodomains RMSD optimized within the FocA family 1.88 Å (4FC4), 1.17 Å/2.11 Å (3KLZ), 1.55 Å/2.06 Å (3KCV), highlighting for each pair of protodomains in a structure the conserved residues in RED. Some residues are conserved at the domain level, and at the family level. Foca show a partial "internal" conservation between TM3 and TM6 at the family level, with especially a [G]**NxxG**[G] **motif**, while within each individual representative, internal" homology is significant. A larger sequence alignment of the Pfam01226 family confirms that motif.

Protodomains RMSD optimized within the Aquaporin family 1.37 Å (5I32), 0.80 Å/1.20 Å (3NE2), , 0.78 Å/1.58 Å (5DYE), highlighting for each pair of protodomains in a structure the conserved residues in RED. Some residues are conserved at the domain level, and at the family level. Aquaporins has retained a higher internal homology between protodomains than FoCA, especially with its **NPA motif** in TM2b and TM5b at the family level.

Protodomains RMSD across FoCA and Aquaporin families. The first protodomain of **FocA vs. other FocA protodomains** 1.88 Å (4FC4), 1.18 Å/2.05 Å (3KLZ), 1.54 Å/2.00 Å (3KCV) **vs. AQP protodomains** 2.17 Å/2.82 Å (5I32), 2.25 Å/2.43 Å (3NE2), 2.33 Å/2.94 Å (5DYE), 2.21 Å/2.71 Å (3C02). While the structure match is very good between any representative of these two families FocA and Aquaporin, the sequence match is rather poor overall, yet the FoCA TM3 motif region shows some overlapping homology with AQP [G/A]xxx[G/S][G/A/S] motif. See Figure S10 for sequence vs. structure similarities.

Pairwise GPCR protodomain alignments

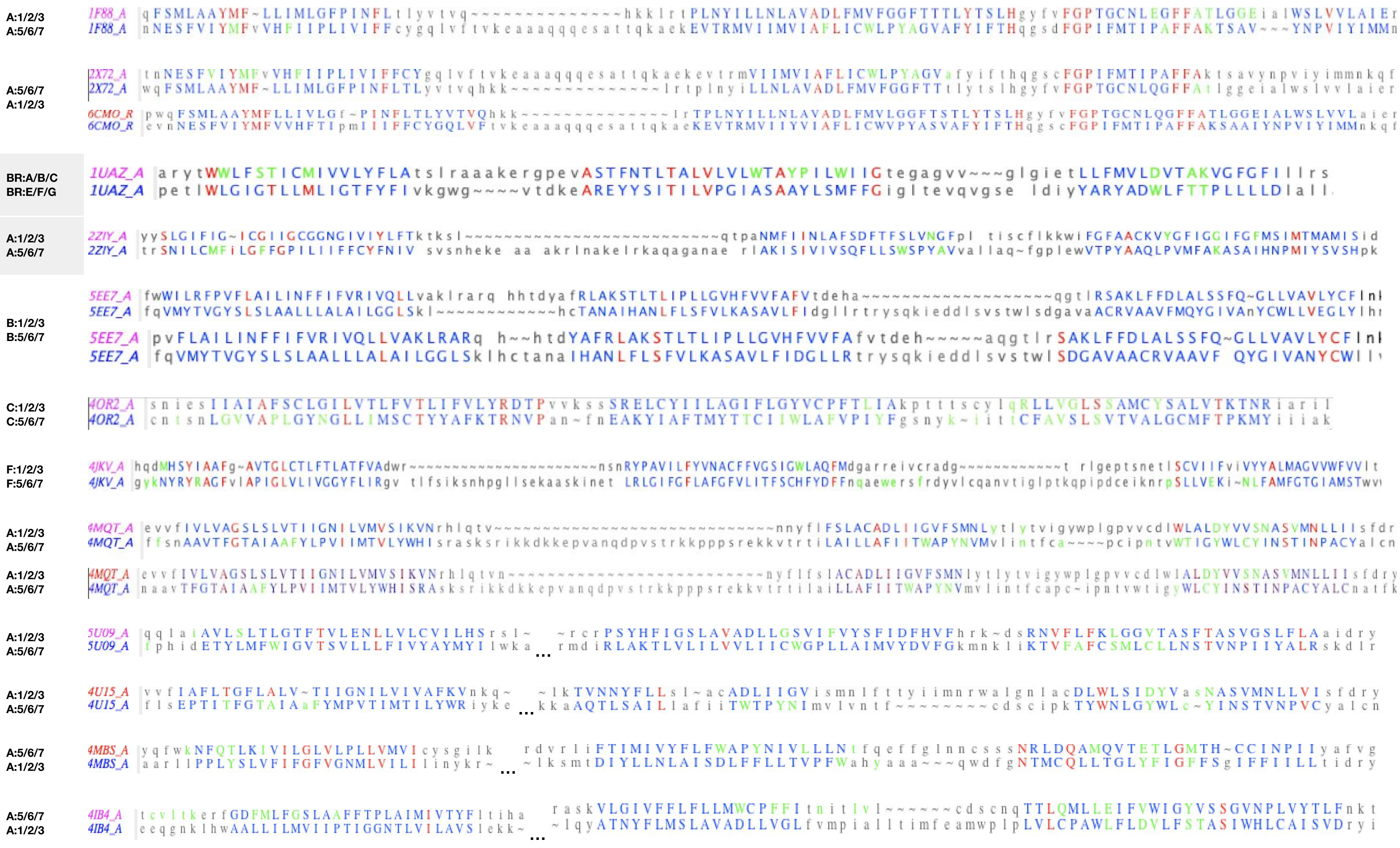

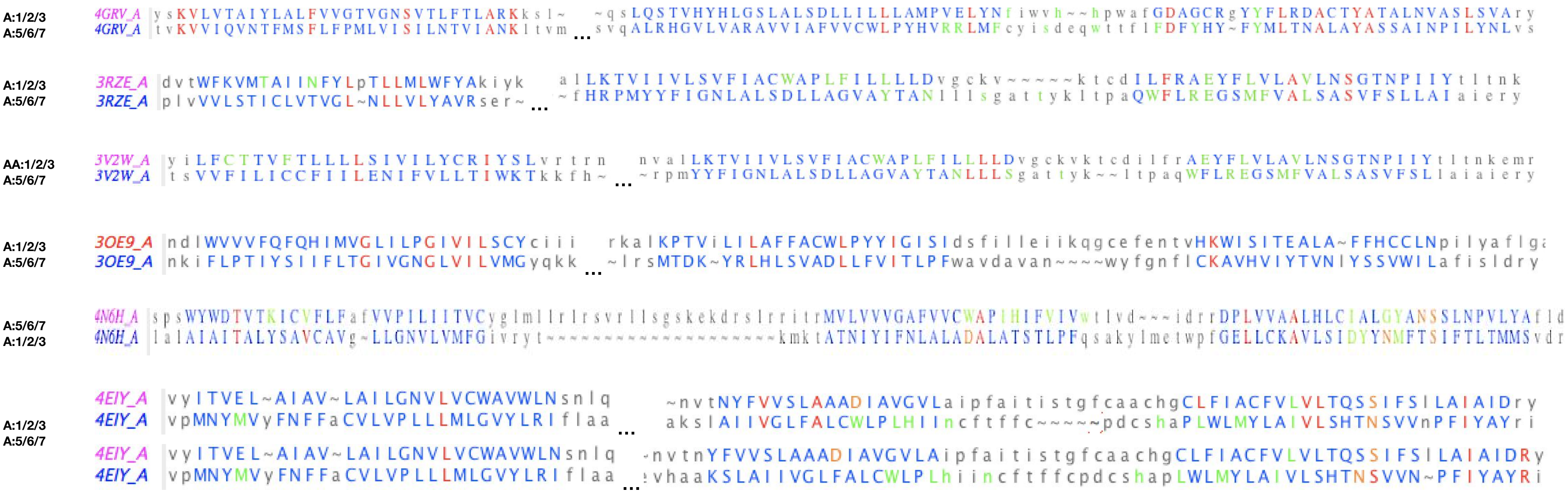

Figure S3.A - Protodomains pairwise alignment (TM123 vs. TM567) of GPCRs. (Class A) Rhodopsin - 1F88: 3.24 Å; (A) Rhodopsin active 2X72: 2.59 Å; Rhodopsin active 6CMO: 3.26 Å; (B) 5EE7: 2.56 Å, alternate 3.11 Å, (C) 4OR2: 3.36 Å, (F) 4JKV: 2.39 Å; (A) active 4MQT: 2.73 Å - human M2 muscarinic acetylcholine receptor; (A) 4U15: 2.6 Å M3 muscarinic receptor bound to tiotropium; (A) 4MBS: 2.62 Å CCR5 chemokine receptor-HIV entry inhibitor maraviroc complex; (A) 4IB4: 2.71 Å; (A) 4GRV: 3.31 Å; (A) 3RZE: 3.01 Å, (A) 3V2W: 2.73 Å (FD), alternate 2.68 Å (WD) ; (A) 3OE9: 3.38 Å; (A) 4N6H: 2.87 Å human delta-opioid receptor (delta-OR) bound to Sodium (orange) and Naltrindole (green); (A) 4EIY: 2.68 Å (WD), alternate 2.89 Å (FD). Conserved residues are colored in red. Ligand binding/proximity (less than 4 Å) colored in green; Sodium binding residues colored in orange. (FD) means the alignment matches F6.44 with D2.50. (WD) matches W6.48 with D2.50. In most cases both alignments can be performed, as a rigid body translation along the symmetry axis of one protodomain vs. the other, in some cases TMH2/6 translation alone. Examples 3V2W and 4EIY are two cases where RMSD is low and similar for both alignments. In the case of Active vs. Inactive Rhodopsin, and alignment of protodomains gets them all within the same RMSD range: 6CMO protodomain 1 vs. 1F88 protodomain 1 : RMSD=1.78 Å; protodomain 2 RMSD=2.88 Å. **The associated identity scores are reported in Table S3 and discussed in the text**.

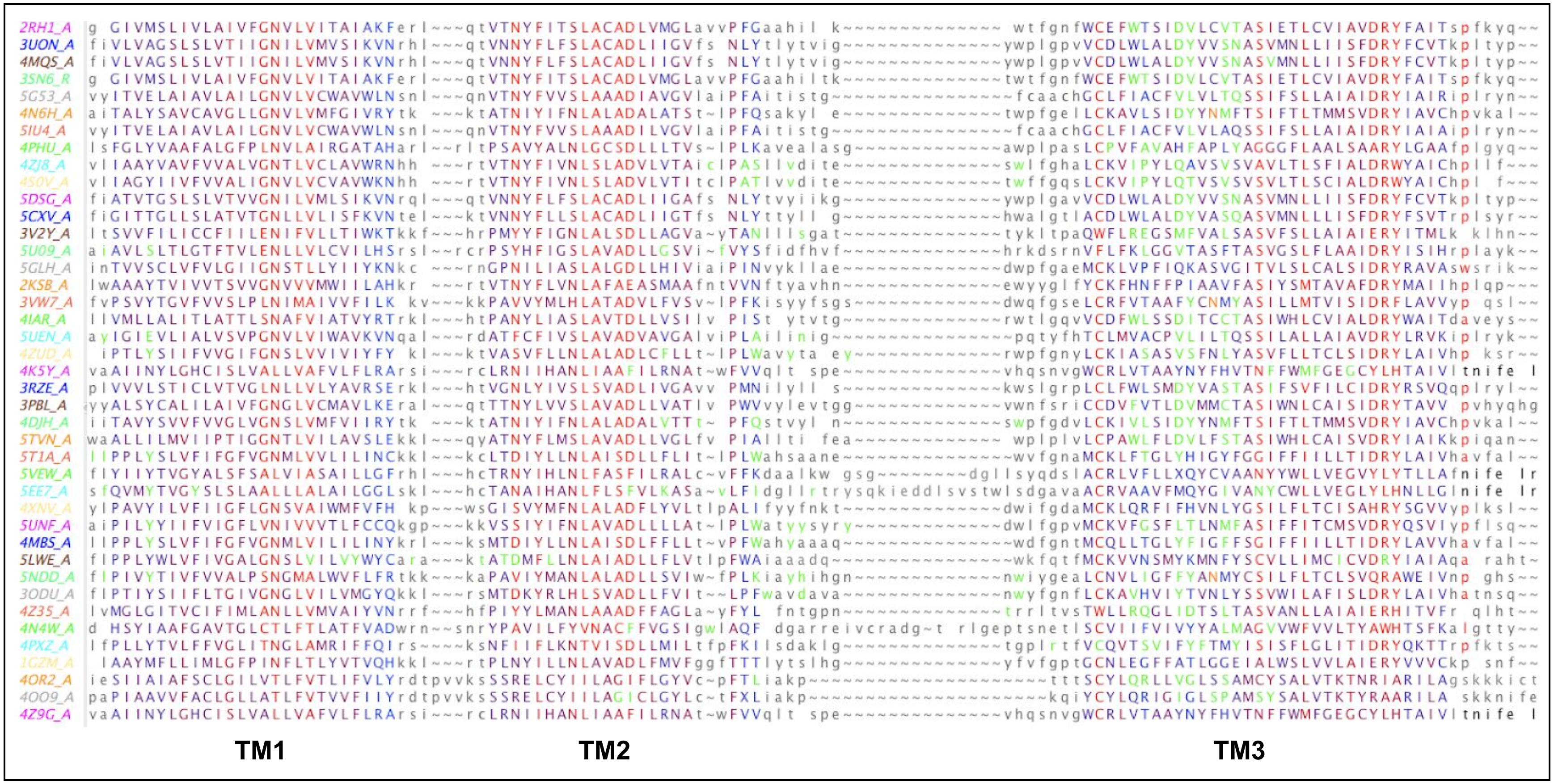
2RH1_A
3UON_A
4MQS_A
3SN6_R
5G53_A
4N6H_A
5IU4_A
4PHU_A
4ZJ8_A
4S0V_A
5DSG_A
5CXV_A
3V2Y_A
5U09_A
5GLH_A
2KSB_A
3VW7_A
4IAR_A
5UEN_A
4ZUD_A
4K5Y_A
3RZE_A
3PBL_A
4DJH_A
5TVN_A
5T1A_A
5VEW_A
5EE7_A
4XNV_A
5UNF_A
4MBS_A
5LWE_A
5NDD_A
3ODU_A
4Z35_A
4N4W_A
4PXZ_A
1GZM_A
4OR2_A
4OO9_A
4Z9G_A
TM1
TM2
TM3

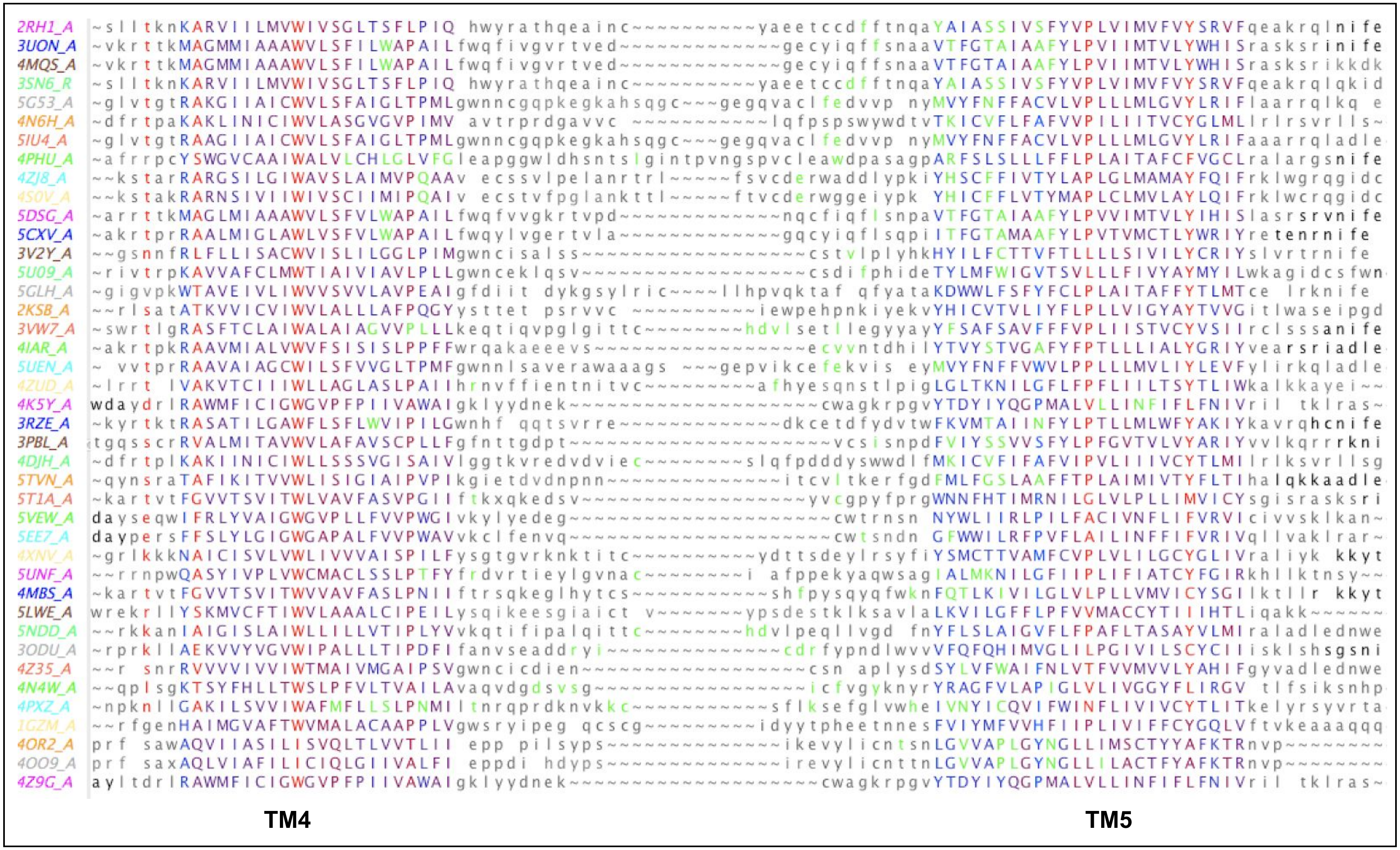
2RH1_A
3UON_A
4MQS_A
3SN6_R
5G53_A
4N6H_A
5IU4_A
4PHU_A
4ZJ8_A
4S0V_A
5DSG_A
5CXV_A
3V2Y_A
5U09_A
5GLH_A
2KSB_A
3VW7_A
4IAR_A
5UEN_A
4ZUD_A
4K5Y_A
3RZE_A
3PBL_A
4DJH_A
5TVN_A
5T1A_A
5VEW_A
5EE7_A
4XNV_A
5UNF_A
4MBS_A
5LWE_A
5NDD_A
3ODU_A
4Z35_A
4N4W_A
4PXZ_A
1GZM_A
4OR2_A
4OO9_A
4Z9G_A
TM4
TM5

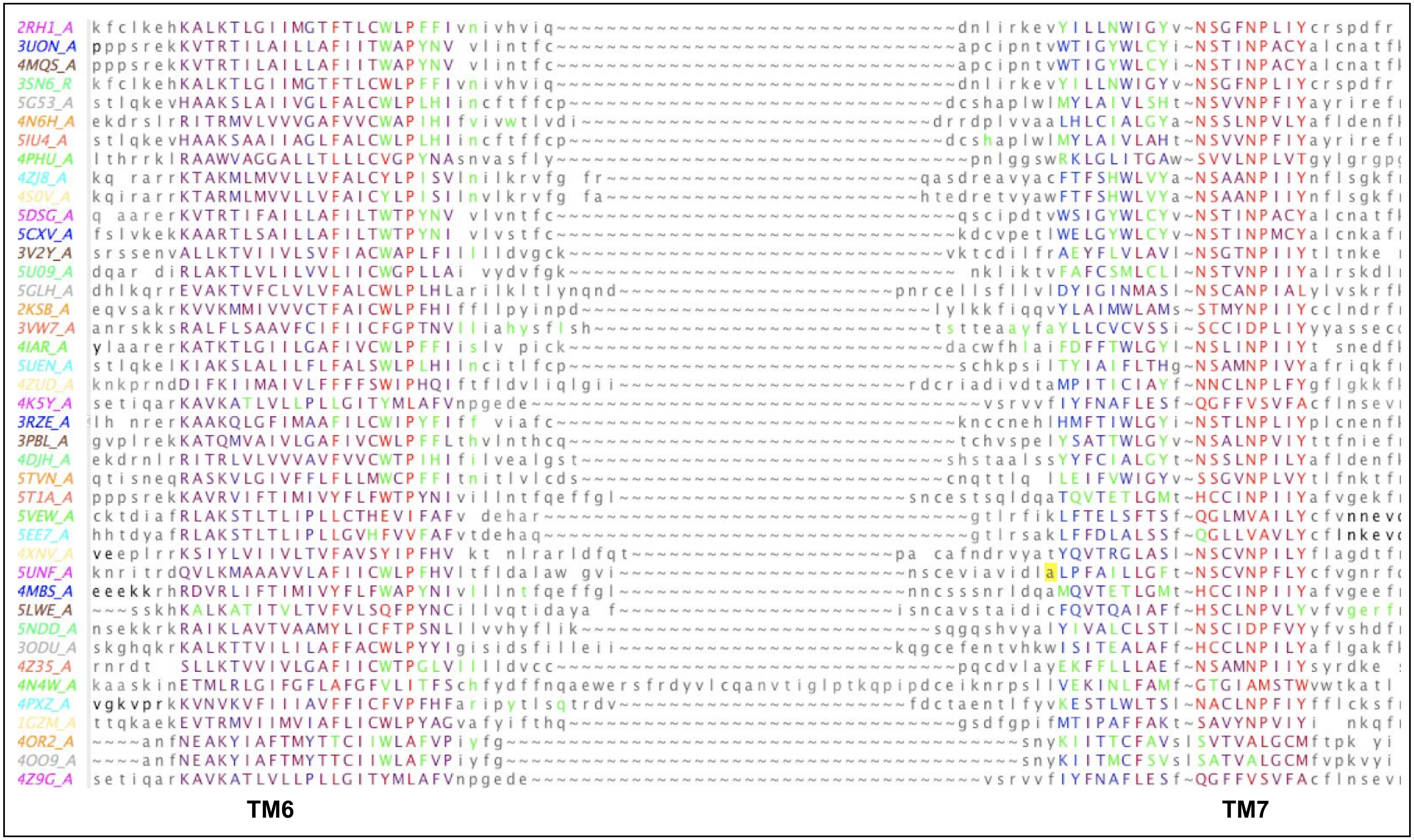

Figure S3.B - GPCR domains TM1-TM7 multiple alignment - (mostly Class A). Red = conserved, Green = ligand binding

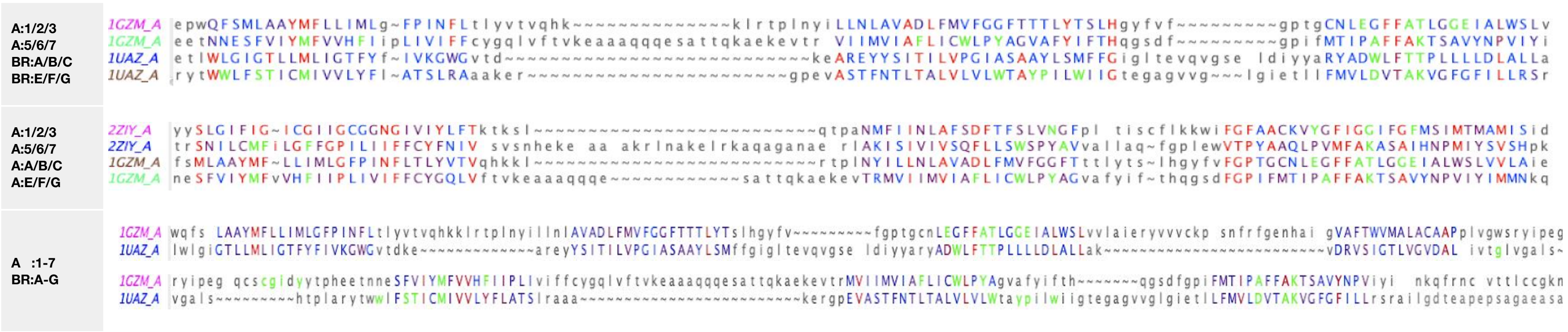

Figure S3.C - 1- Protodomains alignment of Rhodopsin (TM123 vs. TM567) vs. Bacteriorhodopsin (BR) (TM ABC vs. EFG). (Class A) Bovine Rhodopsin (1GZM) protodomain 1 vs. protodomain 2: 3.11 Å; vs. Halobacterium salinarum bacteriorhodopsin (1UAZ) protodomain 1 2.58 Å, protodomain 2 3.69 Å. The sequence symmetry pattern is unique to each, yet a Methionine Identity match in TM1 and TM5 with the TM5 Methionine in contact with retinal in addition to the Shiff base linkage in TM7 xxAK. Similarly to Rhodopsin, BR shows a T in TM3 residue in a symmetrically equivalent position to K in TM7, as if that pair had been inherited from an ancestor in a divergent scenario, or coevolved in a similar way in a pseudo-symmetric coevolution scenario. The TM3/TM7 pseudo symmetric match in structure and partially in sequence is common to both with some of the key binding residues,but most noticeable key binding positions. The orientation of the trans-retinal is different, due to a larger number of aromatic residues, especially W residues at various positions, shifted in position. Most noticeable the (Fxxx)WxxY motif Rhodopsin TM6, binding retinal in both is shifted along the TM6 helix with an . **2- Protodomains alignment (TM123 vs. TM567) of Bovine Rhodopsin vs. Squid Rhodopsin (BR) TM ABC vs. EFG).** (RMSD 3.22/1.28/3.22A resp.) Here the (FFA)K/T motif in TM7/TM3 has evolved dissymmetrically as (MFA)K vs. (FIG)G in the rhodopsin family itself, where trans-retinal ligand and the full rhodopsin structures are superimposable within 1.43A over 259 residues. Both have the protodomain "canonical" symmetry related pair match D in TM3 vs FxxxW in TM7, and most of the same retinal binding residues (highlighted in green) 3**- Domain alignment** for reference Bovine Rhodopsin (1GZM) vs. Halobacterium salinarum bacteriorhodopsin (1UAZ) RMSD = 3.13A while Bovine and Quid Rhodopsins will match with an overall RMSD = 1.43A, and possess all the canonical GPCR Class A conserved residue.

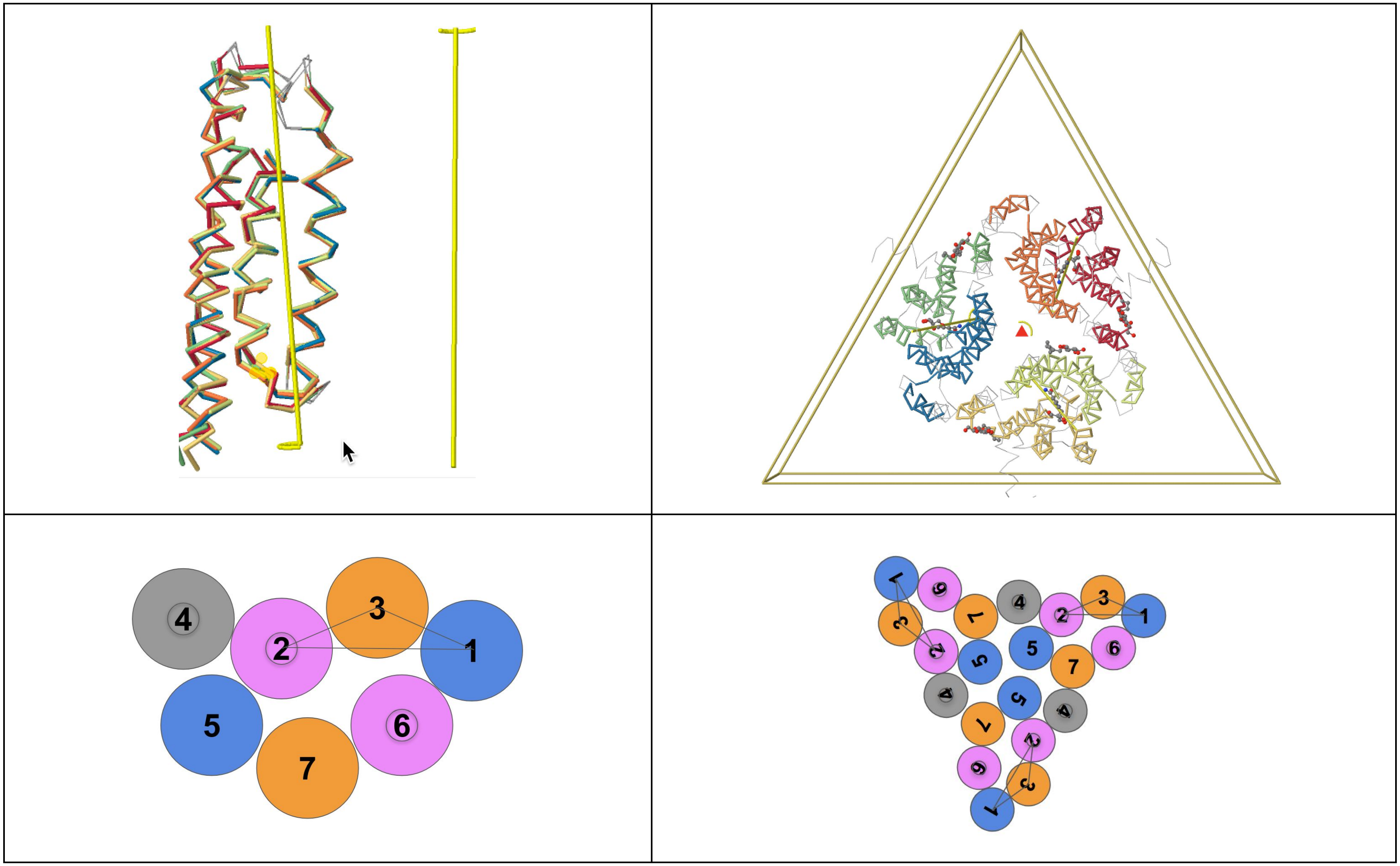

Figure S4 - Protodomains, Domain Pseudo-symmetry and Quaternary symmetry - SWEET Protodomain topology parallel C2 132c/s21a

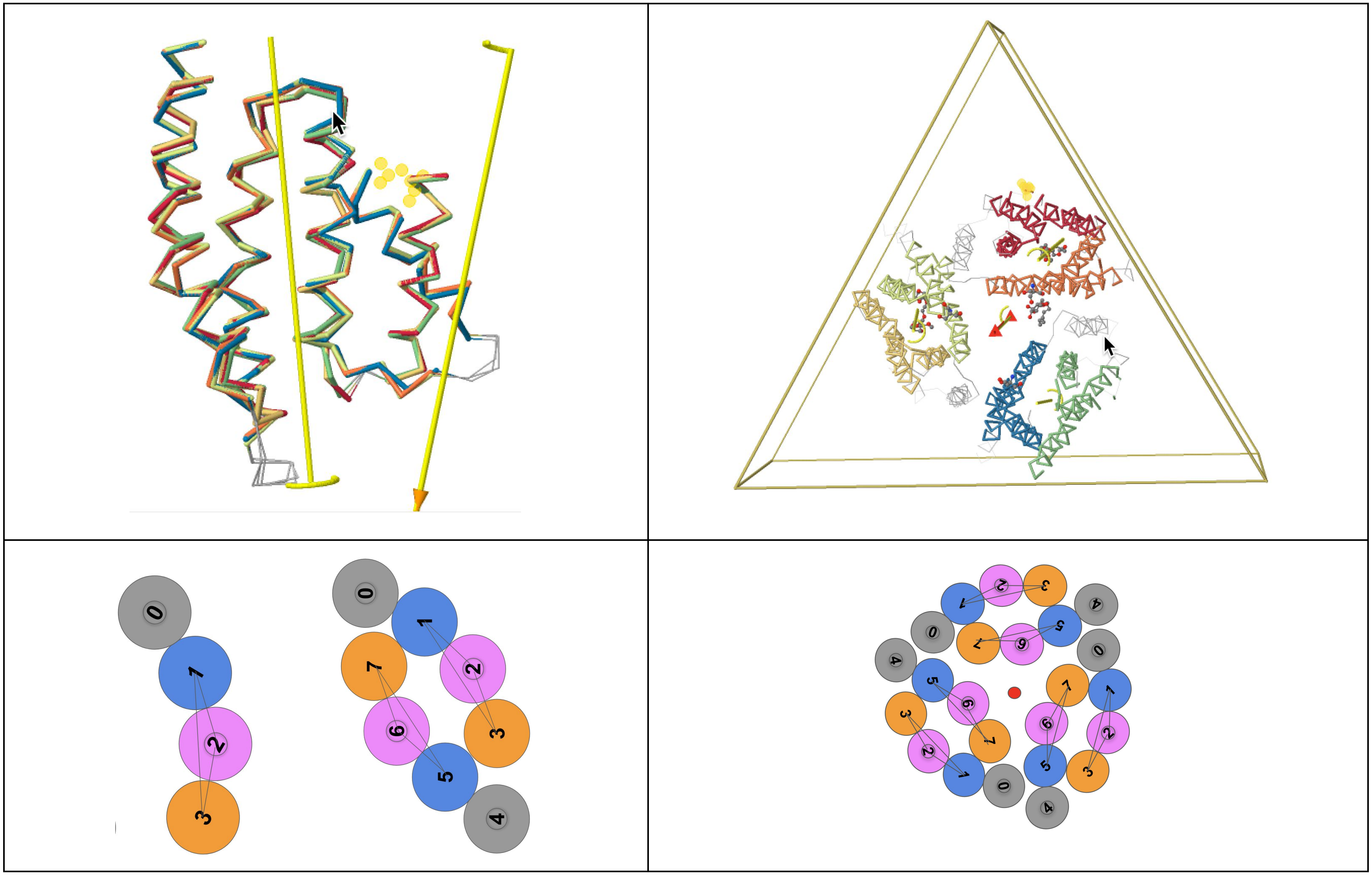

Figure S5 - Protodomains, Domain Pseudo-symmetry and Quaternary symmetry - PnuC Protodomain topology parallel C2 123c/s31p

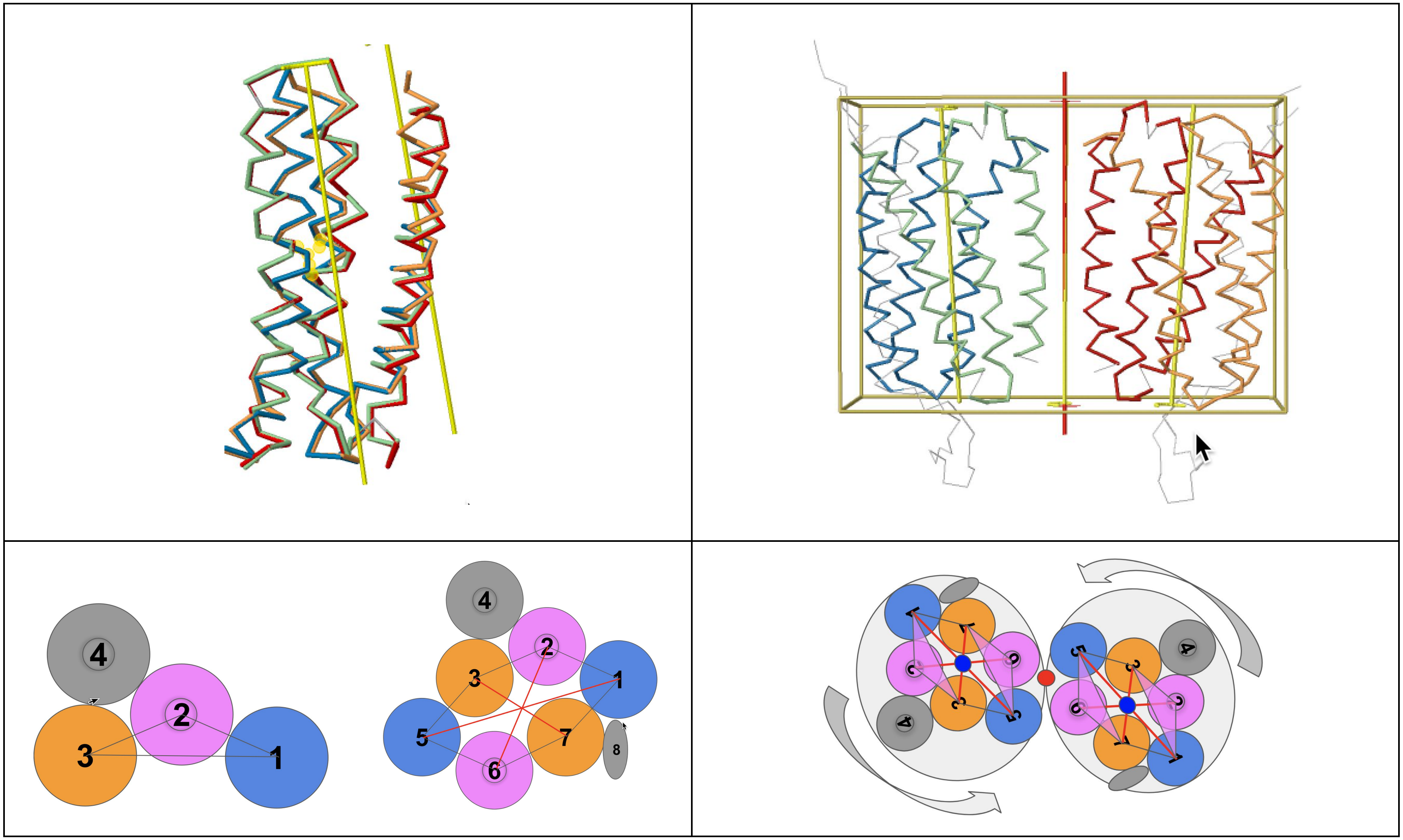

Figure S6 - Protodomains, Domain Pseudo-symmetry and Quaternary symmetry - GPCR Protodomain topology parallel C2 123cc/s31p.

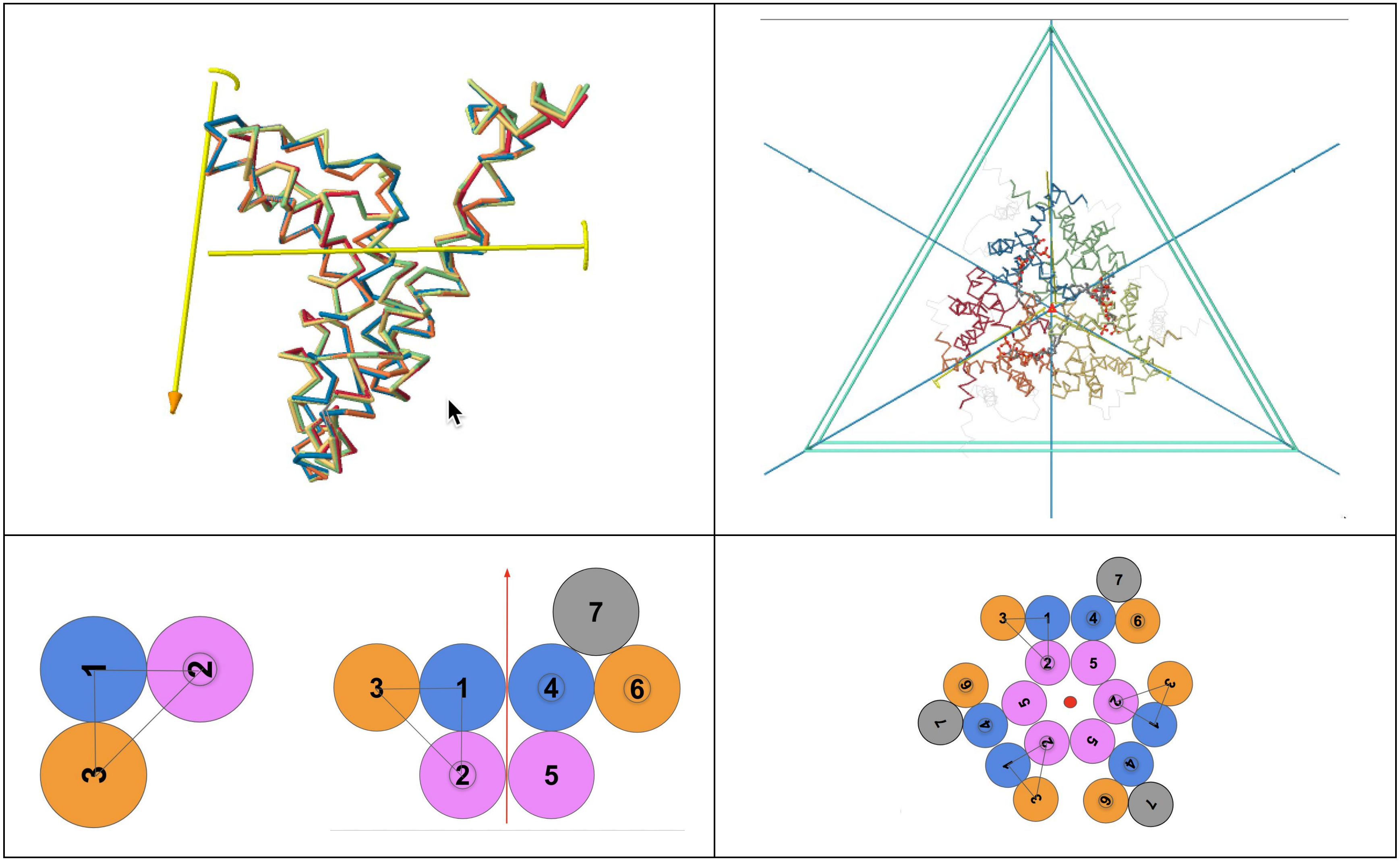

Figure S7 - Protodomains, Domain Pseudo-symmetry and Quaternary symmetry - TRIC Protodomain topology inverted 312c/s11a22a

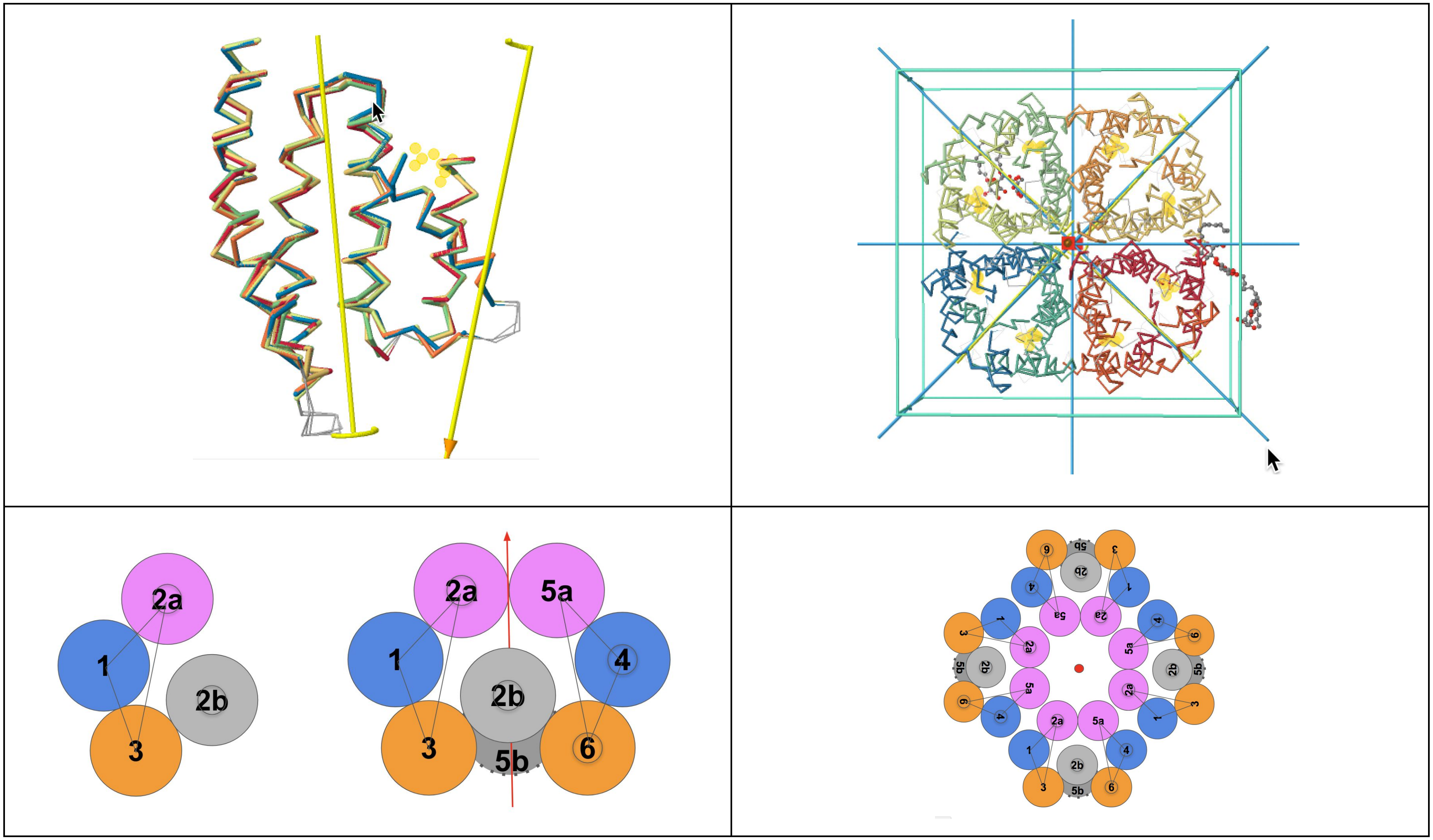

Figure S8 - Protodomains, Domain Pseudo-symmetry and Quaternary symmetry - Aquaporin Protodomain topology inverted C2 312cc/a22a [forms a tetramer quaternary structure, FocA uses the same protodomain and forms a pentamer]. This is the only example with an asymmetric (a) 22 interface, of a very peculiar and idiosyncratic nature: helix 2 splits in 2 forming 2a and 2b to with a helix 2a-2a antiparallel interface from each protodomain on one side and 2b-2b on the other where the latter stack on top of each other one going up the other down - **iCn3D 3D visualization**

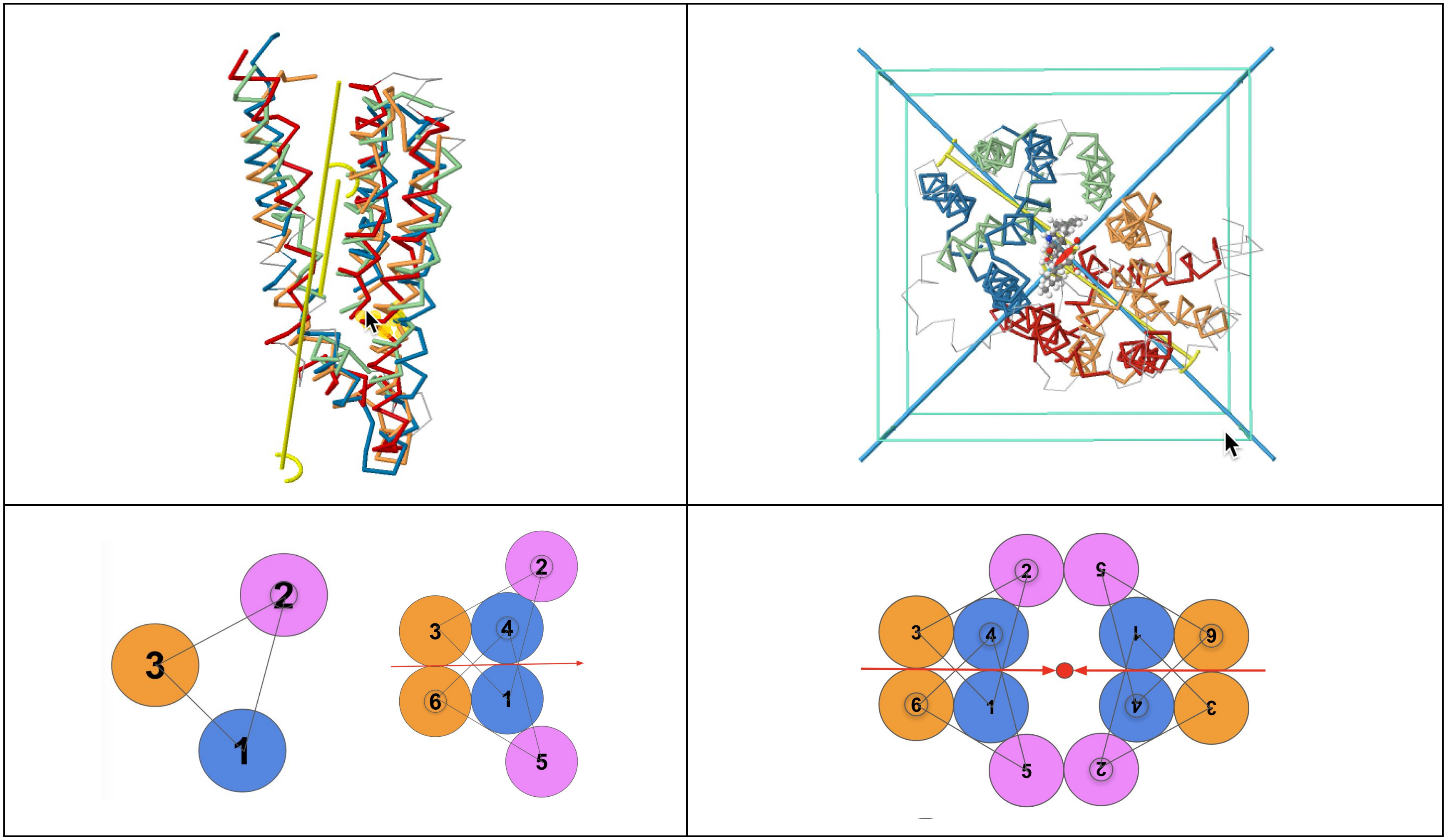

Figure S9 - Protodomains, Domain Pseudo-symmetry and Quaternary symmetry - MFS Protodomain topology inverted C2 interdigitated 132cc/s11a33a MFS - **Lower left)** Inverted Interdigitated Protodomains forming a C2 symmetric domain. It possesses two locally symmetric interfaces TM1-TM1 and TM3-TM3 (s11a33a) **Lower right)** C2 symmetric domain packing through a double (symmetry related) TM2-TM2 interface (2*s22a) formed by the second helix of each of the 4 protodomains. The 2-domains/4-protodomains MFS protein has an overall D2 symmetry. (PDB: 5EQI) since the internal symmetry axes of each domain colinearize and are orthogonal to the central axis (perpendicular to the membrane planes).

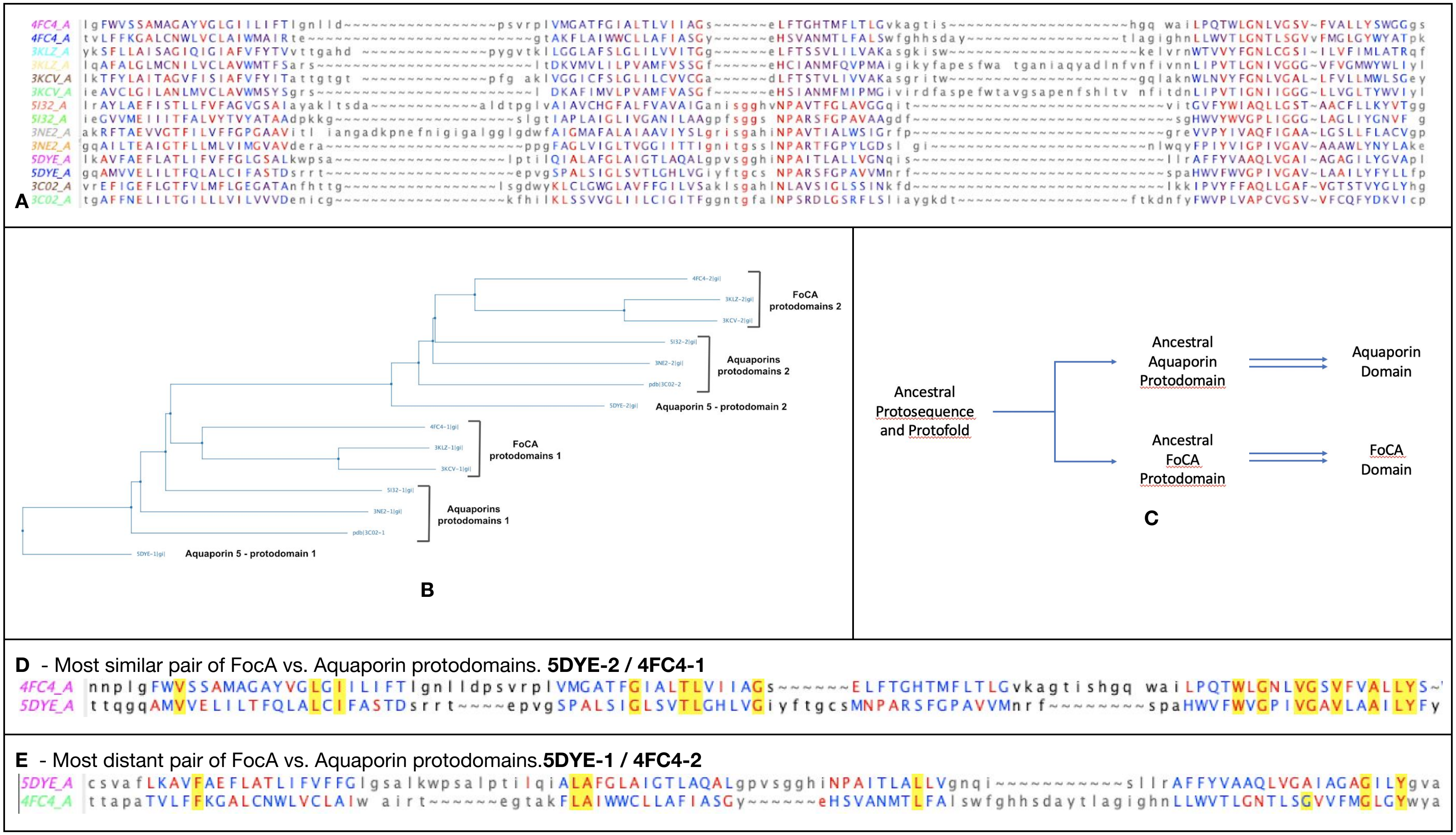

Figure S10: FocA vs Aquaporin A) Structure based multiple sequence alignment of protodomains of FoCA (4FC4, 3KLZ, 3KCV)) and Aquaporins (5DYE, 5I32, 3NE2, 3C02) - RMS relative to the 4FC4-1: 1.88 Å (4FC4-2), 1.18 Å/2.05 Å (3KLZ), 1.54 Å/2.00 Å (3KCV), 2.17 Å/2.82 Å (5I32), 2.25 Å/2.43 Å (3NE2), 2.33 Å/2.94 Å (5DYE), 2.21 Å/2.71 Å (3C02) with a common **G/AxxxG** motif in TM3 **B)** Computed evolutionary tree (using Jalview/Neighbor-Joining/BLOSOM62) from the alignment **C)** Proposed parallel evolutionary mechanism (see text) **D) 4FC4-1** (FocA's first protodomain) vs. **5DYE-2** (Aquaporin's second protodomain) optimized alignment, with surprising sequence similarity in the middle of a hypothetical common evolutionary tree TM1. **The sequence Identity is 21% between protodomains (35% in TM3)** (see Table S2 for details) **E) 4FC4-2** (FocA's second protodomain) vs. **5DYE-2** (Aquaporin's first protodomain) at the extremes of a hypothetical common evolutionary tree in C) -optimized pairwise structural alignment (RMS of 2.16 A) with a sequence Identity is 11% between protodomains (TM3 involving the **G/AxxxG/A motif**). The color red is used for residues conserved between protodomains within a domain, as well as conserved across domains. Note: The alignment of hundreds of sequences for Aquaporin and FocA families do not unveil more common or different patterns than in our set. We consider our dataset as representative]

Foca	TM1	TM2 a	TM2 b	TM3	Protodomain TM1-TM2-TM3	TM4	TM5 a	TM5 b	TM6	Protodomain TM4-TM5-TM6
EC	28	38	-	36	34	39	34	39	33	36
IC	40	52	41	43	44	44	42	-	52	46
Full	34	44	41	40	40	42	38	39	43	41

PnuC	TM0	TM1	TM2	TM3	Protodomain TM1-TM2-TM3	TM4	TM5	TM6	TM7	Protodomain TM5-TM6-TM7
EC	28	50	48	34	44	32	46	46	33	42
IC	28	43	58	46	49	32	57	61	31	50
Full	28	46	51	39	46	32	50	51	32	44

Tric	TM1	TM2	TM3	Protodomain TM1-TM2-TM3	TM4	TM5	TM6	TM7	Protodomain TM4-TM5-TM6
EC	38	57	40	45	41	47	36	28	41
IC	41	48	68	52	40	42	44	27	42
Full	40	53	54	49	41	44	39	28	41

MFS	TM1	TM2	TM3	TM4	TM5	TM6	TM7	TM8	TM9	TM10	TM11	TM12
EC	24	25	28	35	29	31	25	18	23	21	24	20
IC	22	41	35	34	28	30	22	26	21	16	21	23
Full	23	33	31	35	29	30	24	22	22	18	23	22
	Proto 1		29	Proto 2		31	Proto 3		23	Proto 4		21

Figure S11: Sequence Divergence of TMH Families - Sequence similarity score (see Methods section for details) of the aligned EC half, IC half, and Full TM sequences for each of the TMs **TMH families Aquaporin, Foca, PnuC, Tric, and MFS.** Protodomain 1 and 2 scores are given along with those for EC-facing and IC-facing halves. Higher numbers mean high sequence similarity (or higher conservation), where a maximum score of 100 would mean identical sequences or two sequences with similar residues at each position in the sequence alignment. Protodomains specific to each protein family are discussed in the main text and shown in **Figure 1** and Supplementary **Figures S1** through **S9**. The list of proteins and PDB ids used for each family is provided in the Supplement File SF1. We use here a common 3TMH protodomain decomposition, including for PnuC (in the main text we used 4TMH)

```
6CMO_R: chimera protein of Soluble cytochrome b562 and Rhodopsin
1GZM_A: RHODOPSIN

                     10        20        30        40        50        60
                 ....*....|....*....|....*....|....*....|....*....|....*....|
6CMO_R 142 CgtegpnfyvpFSNAtgvvrspfeypqyYLAEPWQFSmlaaymfllivlgfpinfltlyv 201
1GZM_A   3 NgtegpnfyvpFSNKtgvvrspfeapqyYLAEPWQFSmlaaymfllimlgfpinfltlyv 62

                     70        80        90       100       110       120
                 ....*....|....*....|....*....|....*....|....*....|....*....|
6CMO_R 202 tvqhkklrtplnyILLNLAVADLFMVLGGFTSTLYTSlhgyfvfgptgcnlqGFFATLGG 261
1GZM_A  63 tvqhkklrtplnyILLNLAVADLFMVFGGFTTTLYTSlhgyfvfgptgcnleGFFATLGG 122

                    130       140       150       160       170       180
                 ....*....|....*....|....*....|....*....|....*....|....*....|
6CMO_R 262 EIALWSLVVLAIERYVVvckpmsnfrfGENHAIMGVAFTWvmalacaapplagwsryipe 321
1GZM_A 123 EIALWSLVVLAIERYVVvckpmsnfrfGENHAIMGVAFTWvmalacaapplvgwsryipe 182

                    190       200       210       220       230       240
                 ....*....|....*....|....*....|....*....|....*....|....*....|
6CMO_R 322 glqcscgidyytlkpevnnesfviymfvvhftIPMIIIFFCYGQLVFTVKEaaaqqqesa 381
1GZM_A 183 gmqcscgidyytpheetnnesfviymfvvhfiIPLIVIFFCYGQLVFTVKEaaaqqqesa 242

                    250       260       270       280       290       300
                 ....*....|....*....|....*....|....*....|....*....|....*....|
6CMO_R 382 ttqkaekevtrmviiyviaflicwvpyasvafyifthqgsCFgpifmTIPAFFAKsaaiy 441
1GZM_A 243 ttqkaekevtrmviimviaflicwlpyagvafyifthqgsDFgpifmTIPAFFAKtsavy 302

                 ....*....
6CMO_R 442 npviyimmN 450
1GZM_A 303 npviyimmN 311
```

Figure S12 - VAST+ Invariant substructure alignment between the active (6CMO, human) vs, inactive (1GZM bovine) conformations of Rhodopsin - RMSD = 1.70 Å for 106 residues aligned (86% identity) . 3D visualization link: iCn3D
