# Supplementary material for: Pseudo-Symmetric Assembly of Protodomains as a Common Denominator in the Evolution of Polytopic Helical Membrane Proteins
Source: J Mol Evol. 2020 Mar 18;88(4):319–44. doi: 10.1007/s00239-020-09934-4 (PMC7162841; doi:10.1007/s00239-020-09934-4)
Supplement: Supplementary file 2 — Supplementary file SF1 (PDF 2278 kb) [file 239_2020_9934_MOESM2_ESM.pdf]

ACHA7 TM1

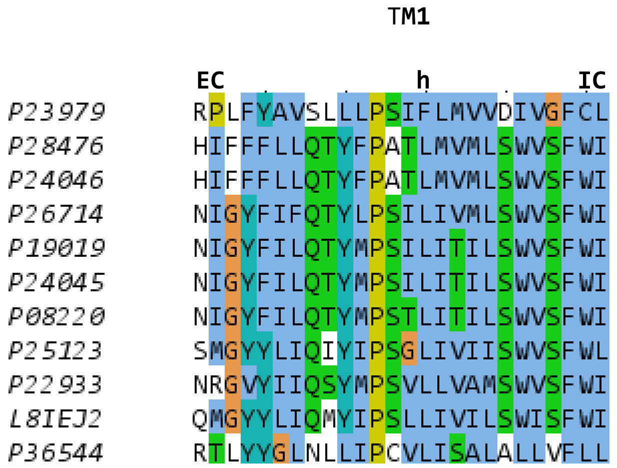

TM1
EC
h
IC
P23979 RPLFYAVSLLLPSIFLMVVDIVGFCL
P28476 HIFFFLLQTYFPATLMVMLSWVSFWI
P24046 HIFFFLLQTYFPATLMVMLSWVSFWI
P26714 NIGYFIFQTYLPSILIVMLSWVSFWI
P19019 NIGYFILQTYMPSILITILSWVSFWI
P24045 NIGYFILQTYMPSILITILSWVSFWI
P08220 NIGYFILQTYMPSTLITILSWVSFWI
P25123 SMGYYLIQIYIPSGLIVIISWVSFWL
P22933 NRGVYIIQSYMPSVLLVAMSWVSFWI
L8IEJ2 QMGYYLIQMYIPSLLIVILSWISFWI
P36544 RTLYYGLNLLIPCVLISALALLVFLL

ACHA7 TM2

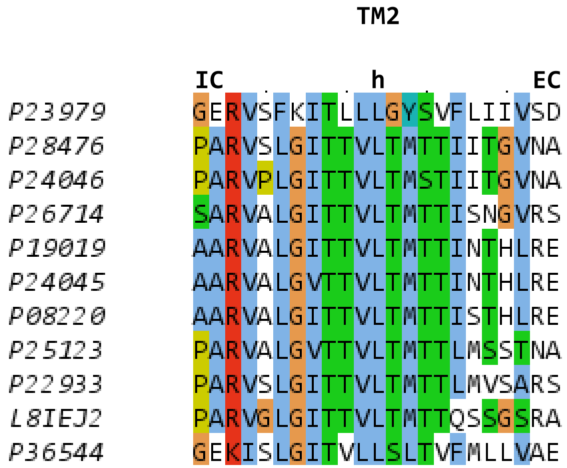
TM2
IC
h
EC
P23979 GERVSFKITLLLGYSVFLIIVSD
P28476 PARVSLGITTVLTMTTIITGVNA
P24046 PARVPLGITTVLTMSTIITGVNA
P26714 SARVALGITTVLTMTTISNGVRS
P19019 AARVALGITTVLTMTTINTHLRE
P24045 AARVALGVTTVLTMTTINTHLRE
P08220 AARVALGITTVLTMTTISTHLRE
P25123 PARVALGVTTVLTMTTLMSSTNA
P22933 PARVSLGITTVLTMTTLMVSARS
L8IEJ2 PARVGLGITTVLTMTTQSSGSRA
P36544 GEKISLGITVLLSLTVFMLLVAE

ACHA7 TM3

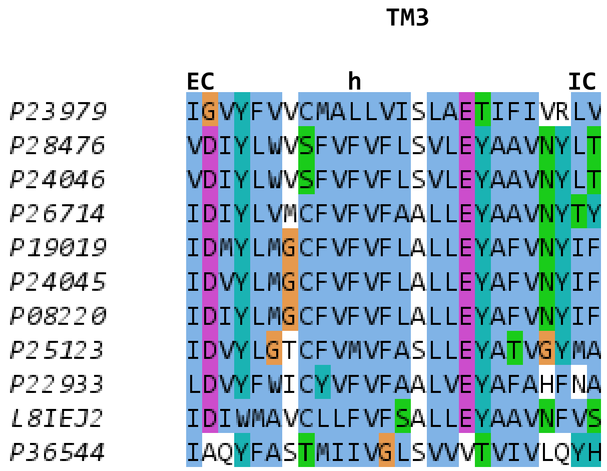

TM3
EC
h
IC
P23979 IGVYFVVCMALLVISLAETIFIVRLV
P28476 VDIYLWVSFVFVFLSVLEYAAVNYLT
P24046 VDIYLWVSFVFVFLSVLEYAAVNYLT
P26714 IDIYLVMCFVFVFAALLEYAAVNYTY
P19019 IDMYLMGCFVFVFLALLEYAFVNYIF
P24045 IDVYLMGCFVFVFLALLEYAFVNYIF
P08220 IDIYLMGCFVFVFLALLEYAFVNYIF
P25123 IDVYLGTCFVMVFASLLEYATVGYMA
P22933 LDVYFWICYVFVFAALVEYAFAHFNA
L8IEJ2 IDIWMAVCLLFVFSALLEYAAVNFVS
P36544 IAQYFASTMIIVGLSVVVTVIVLQYH

ACHA7 TM4

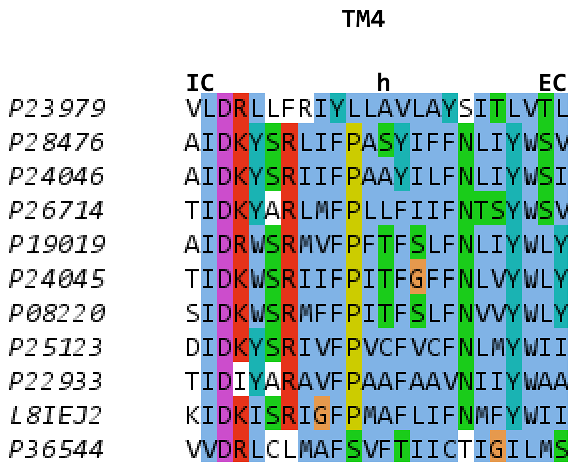
TM4
IC
h
EC
P23979 VLDRLLFRIYLLAVLAYSITLVTL
P28476 AIDKYSRLIFPASYIFFNLIYWSV
P24046 AIDKYSRIIFPAAYILFNLIYWSI
P26714 TIDKYARLMFPLLFIIFNTSYWSV
P19019 AIDRWSRMVFPFTFSLFNLIYWLY
P24045 TIDKWSRIIFPITFGFFNLVYWLY
P08220 SIDKWSRMFFPITFSLFNVVYWLY
P25123 DIDKYSRIVFPVCFVCFNLMYWII
P22933 TIDIYARAVFPAAFAAVNIIYWAA
L8IEJ2 KIDKISRIGFPMAFLIFNMFYWII
P36544 VVDRLCLMAFSVFTIICTIGILMS

Aquaporin TM1

```
                TM1

        IC          h              EC
P29972  KLFWRAVVAEFLATTLFVFISIGSALGFK
P0AER0  STLKGQCIAEFLGTGLLIFFGVGCVAALK
Q6J8I9  ASFWRAIFAEFFATLFYVFFGLGASLRWA
Q9C4Z5  VSLTKRCIAEFIGTFILVFFGAGSAAVTL
P60844  --MFRKLAAECFGTFWLVFGGCGSAVLAA
Q8WPZ6  KSYVREFIGEFLGTFVLMFLGEGATANFH
Q41372  WSFWRAAIAEFIATLLFLYITVATVIGHS
P55064  VAFLKAVFAEFLATLIFVFFGLGSALKWP
P55087  QAFWKAVTAEFLAMLIFVLLSLGSTINWG
Q8UJW4  --MGRKLLAEFFGTFWLVFGGCGSAVFAA
O28846  MTLAKRFTAEVVGTFILVFFGPGAAVITL
F2QVG4  RNHFIAMSGEFVGTFLFLWSAFVIAQIAN
P41181  IAFSRAVFAEFLATLLFVFFGLGSALNWP
Q41951  LASLRAYLAEFISTLLFVFAGVGSAIAYA
P23645  LEFWRSIISECLASFMYVFIVCGAAAGVG
P23900  NTYLKEFLAEFMGTMVMIIFGSAVVCQVN
P06624  ASFWRAICAEFFASLFYVFFGLGASLRWA
Q08451  WSFYRAGIAEFMATFLFLYITILTVMGLK
P43286  WSFYRAVIAEFVATLLFLYITVLTVIGYK
P25818  PDALKAALAEFISTLIFVVAGSGSGMAFN
P26587  PDSIRATLAEFLSTFVFVFAAEGSILSLD
P08995  VPFLQKLVAEAVGTYFLIFAGCASLVVNE
O24389  VGSLKAYLAEFIATLLFVFAGVGSAIAYN
```

Aquaporin TM2

```
                TM2

          EC         h          IC
P29972    DNVKVSLAFGLSIATLAQSVGHIS
P0AER0    GQWEISVIWGLGVAMAIYLTAGVS
Q6J8I9    HVLQVALAFGLALATLVQAVGHIS
Q9C4Z5    DWVAIGLAFGFAIAASIYALGNIS
P60844    GFAGVALAFGLTVLTMAFAVGHIS
Q8WPZ6    DWYKLCLGWGLAVFFGILVSAKLS
Q41372    GLLGIAWAFGGMIFVLVYCTAGIS
P55064    TILQIALAFGLAIGTLAQALGPVS
P55087    DMVLISLCFGLSIATMVQCFGHIS
Q8UJW4    GFTGVALAFGLTVLTMAYAVGGIS
O28846    DWFAIGMAFALAIAAVIYSLGRIS
F2QVG4    QLIMISFGFGFGVMVGVFITYRVS
P41181    SVLQIAMAFGLGIGTLVQALGHIS
Q41951    GLVAIAVCHGFALFVAVAIGANIS
P23645    VLLATALASGLAMATLTQCFLHIS
P23900    TFDDVALGWAAAVVMGYFCAGGSA
P06624    HVLQVALAFGLALATLVQAVGHIS
Q08451    GIQGVAWAFGGMIFALVYCTAGIS
P43286    GILGIAWAFGGMIFILVYCTAGIS
P25818    GLVAAAVAHAFGLFVAVSVGANIS
P26587    GLILVALAHAFALFAAVSAAINVS
P08995    TFPGIAIVWGLVLTVLVYTVGHIS
O24389    GLVAIAVAHAFALFVGVSMAANIS
```

Aquaporin TM2B

	**TM2B**
	**IC**
P29972	PAVTLGLLLSC
P0AER0	PAVTIALWLFA
Q6J8I9	PAVTFAFLVGS
Q9C4Z5	PAVTIGLWSVK
P60844	PAVTIGLWAGG
Q8WPZ6	LAVSIGLSSIN
Q41372	PAVTFGLFLAR
P55064	PAITLALLVGN
P55087	PAVTVAMVCTR
Q8UJW4	PAVSVGLTVAG
O28846	PAVTIALWSIG
F2QVG4	PAVTLALVLAR
P41181	PAVTVACLVGC
Q41951	PAVTFGLAVGG
P23645	PAVTLALCVVR
P23900	PSITLANLVYR
P06624	PAVTFAFLVGS
Q08451	PAVTFGLFLAR
P43286	PAVTFGLFLAR
P25818	PAVTFGAFIGG
P26587	PAVTFGALVGG
P08995	PAVTIAFASTR
O24389	PAVTLGLAVGG

Aquaporin TM3

```
                 TM3

        IC          h          EC
P29972  FRALMYIIAQCVGAIVATAILSGIT
P0AER0  RKVIPFIVSQVAGAFCAAALVYGLY
Q6J8I9  LRAICYVVAQLLGAVAGAAVLYSVT
Q9C4Z5  REVVPYIIAQLLGAAFGSFIFLQCA
P60844  KEVVGYVIAQVVGGIVAAALLYLIA
Q8WPZ6  KKIPVYFFAQLLGAFVGTSTVYGLY
Q41372  LRALVYMIAQCLGAICGVGLVKAFM
P55064  LRAFFYVAAQLVGAIAGAGILYGVA
P55087  AKSVFYIAAQCLGAIIGAGILYLVT
Q8UJW4  SSLVPYVIAQVAGAIVAAAALYVIA
O28846  REVVPYIVAQFIGAALGSLLFLACV
F2QVG4  PFRGILMAFTQIVAGMAAAGAASAM
P41181  LRAAFYVAAQLLGAVAGAALLHEIT
Q41951  ITGVFYWIAQLLGSTAACFLLKYVT
P23645  IRAAMYITAQCGGGIAGAALLYGVT
P23900  KKVPYYFAGQLIGAFTGALILFIWY
P06624  LRAICYMVAQLLGAVAGAAVLYSVT
Q08451  TRAVFYMVMQCLGAICGAGVVKGFM
P43286  PRALLYIIAQCLGAICGVGFVKAFQ
P25818  LRGILYWIAQLLGSVVACLILKFAT
P26587  IRAIYYWIAQLLGAILACLLLRLTT
P08995  IQVPAYVVAQLLGSILASGTLRLLF
O24389  LTGLFYWVAQLLGSTVACLLLKYVT
```

Aquaporin TM4

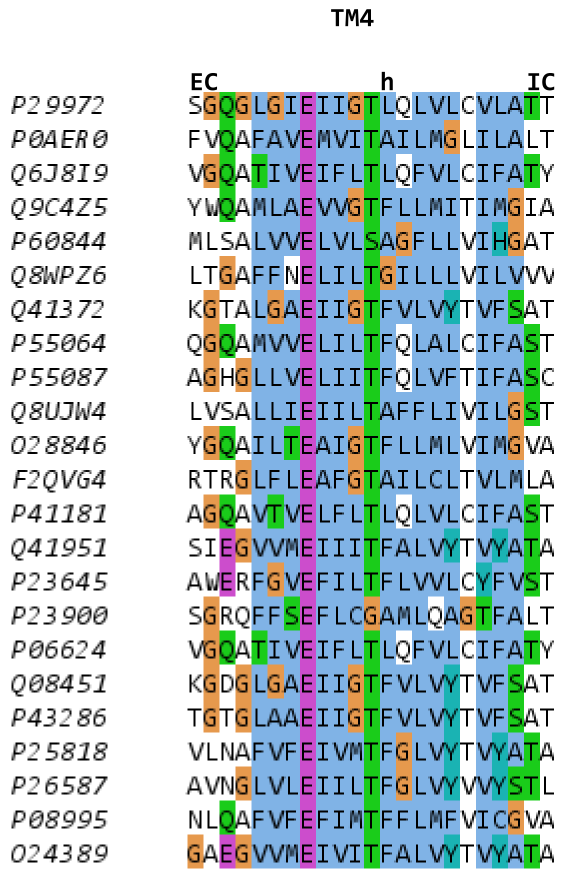

Aquaporin TM5

```
                TM5

        IC      h          EC
P29972  SAPLAIGLSVALGHLLAIDYT
P0AER0  LAPLLIGLLIAVIGASMGPLT
Q6J8I9  SVALAVGFSLTLGHLFGMYYT
Q9C4Z5  FAGIIIGLTVAGIITTLGNIS
P60844  FAPIAIGLALTLIHLISIPVT
Q8WPZ6  KLSSVVGLIILCIGITFGGNT
Q41372  LAPLPIGFAVFMVHLATIPIT
P55064  SPALSIGLSVTLGHLVGIYFT
P55087  SIALAIGFSVAIGHLFAINYT
Q8UJW4  FAPIAIGLALTLIHLISIPVT
O28846  FAGLVIGLTVGGIITTIGNIT
F2QVG4  FAPFVIGIALLIAHLICIYYT
P41181  TPALSIGFSVALGHLLGIHYT
Q41951  IAPLAIGLIVGANILAAGPFS
P23645  NSAASIGCAYSACCFVSMPYL
P23900  VFPLMMFILIFIINASMAYQT
P06624  SVALAVGFSLTLGHLFGMYYT
Q08451  LAPLPIGFAVFLVHLATIPIT
P43286  LAPLPIGFAVFMVHLATIPIT
P25818  IAPIAIGFIVGANILAGGAFS
P26587  IAPLAIGLIVGANILVGGPFS
P08995  FAGIAIGSTLLLNVIIGGPVT
O24389  IAPIAIGFIVGANILAAGPFS
```

Aquaporin TM5B

TM5B

	EC
P29972	PARSFGSAVITH
P0AER0	PARDFGPKVFAW
Q6J8I9	PARSFAPAILTR
Q9C4Z5	PARTFGPYLNDM
P60844	PARSTAVAIFQG
Q8WPZ6	PSRDLGSRFLSL
Q41372	PARSFGAAVIFN
P55064	PARSFGPAVVMN
P55087	PARSFGPAVIMG
Q8UJW4	PARSTGQALFVG
O28846	PARTFGPYLGDS
F2QVG4	PARSFGPAVAAR
P41181	PARSLAPAVVTG
Q41951	PARSFGPAVAAG
P23645	PARSLGPSFVLN
P23900	LARDLGPRLALY
P06624	PARSFAPAILTR
Q08451	PARSLGAAIIYN
P43286	PARSFGAAVIYN
P25818	PAVAFGPAVVSW
P26587	PARAFGPALVGW
P08995	PARSLGPAFVHG
O24389	PARSFGPAVVAG

Aquaporin TM6

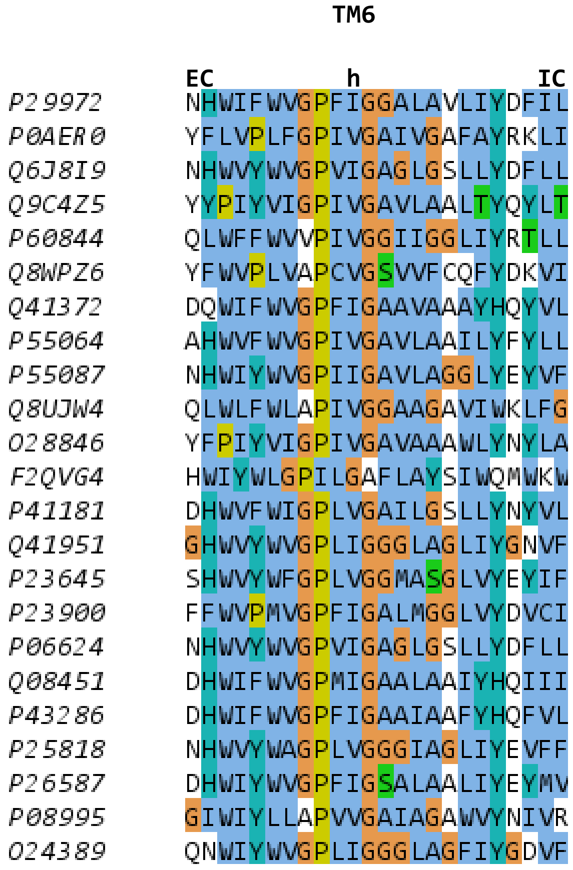

Foca TM1

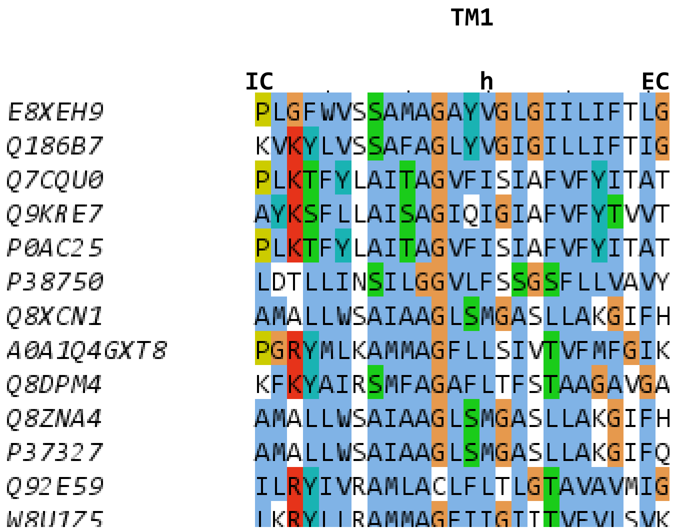

TM1
IC
h
EC
E8XEH9 PLGFWVSSAMAGAYVGLGIILIFTLG
Q186B7 KVKYLVSSAFAGLYVGIGILLIFTIG
Q7CQU0 PLKTFYLAITAGVFISIAFVFYITAT
Q9KRE7 AYKSFLLAISAGIQIGIAFVFYTVVT
P0AC25 PLKTFYLAITAGVFISIAFVFYITAT
P38750 LDTLLINSILGGVLFSSGSFLLVAVY
Q8XCN1 AMALLWSAIAAGLSMGASLLAKGIFH
A0A1Q4GXT8 PGRYMLKAMMAGFLLSIVTVFMFGIK
Q8DPM4 KFKYAIRSMFAGAFLTFSTAAGAVGA
Q8ZNA4 AMALLWSAIAAGLSMGASLLAKGIFH
P37327 AMALLWSAIAAGLSMGASLLAKGIFQ
Q92E59 ILRYIVRAMLACLFLTLGTAVAVMIG

Foca TM2

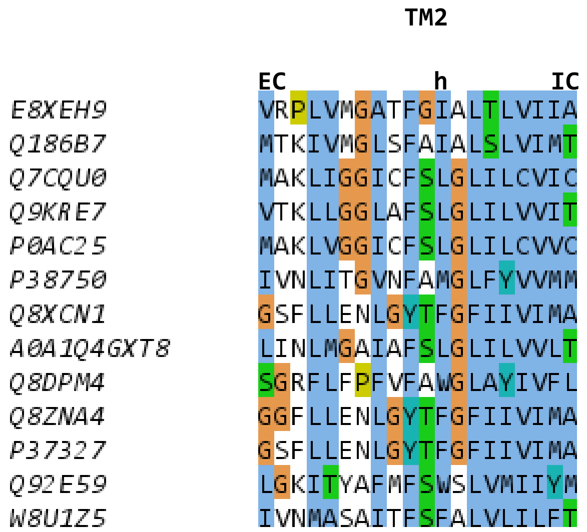

TM2
EC
h
IC
E8XEH9 VRPLVMGATFGIALTLVIIA
Q186B7 MTKIVMGLSFAIALSLVIMT
Q7CQU0 MAKLIGGICFSLGLILCVIC
Q9KRE7 VTKLLGGLAFSLGLILVVIT
P0AC25 MAKLVGGICFSLGLILCVVC
P38750 IVNLITGVNFAMGLFYVVMM
Q8XCN1 GSFLLENLGYTFGFIIVIMA
A0A1Q4GXT8 LINLMGAIAFSLGLILVVLT
Q8DPM4 SGRFLFPFVFAWGLAYIVFL
Q8ZNA4 GGFLLENLGYTFGFIIVIMA
P37327 GSFLLENLGYTFGFIIVIMA
Q92E59 LGKITYAFMFSWSLVMIIYM
W8U1Z5 IVNMASAITFSFALVLILFT

Foca TM2B

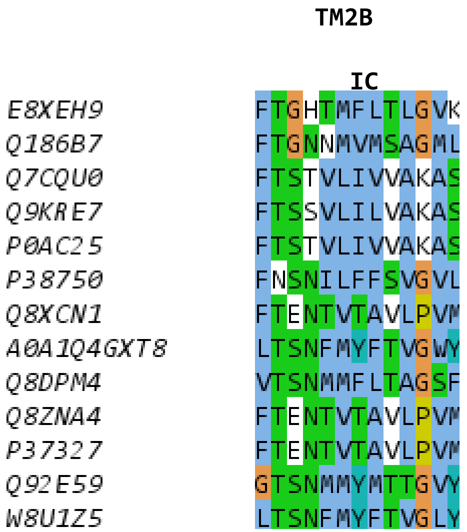
TM2B
IC
E8XEH9 FTGHTMFLTLGVK
Q186B7 FTGNNMVMSAGML
Q7CQU0 FTSTVLIVVAKAS
Q9KRE7 FTSSVLILVAKAS
P0AC25 FTSTVLIVVAKAS
P38750 FNSNILFFSVGVL
Q8XCN1 FTENTVTAVLPVM
A0A1Q4GXT8 LTSNFMYFTVGWY
Q8DPM4 VTSNMMFLTAGSF
Q8ZNA4 FTENTVTAVLPVM
P37327 FTENTVTAVLPVM
Q92E59 GTSNMMYMTTGVY
W8U1Z5 LTSNFMYFTVGLY

Foca TM3

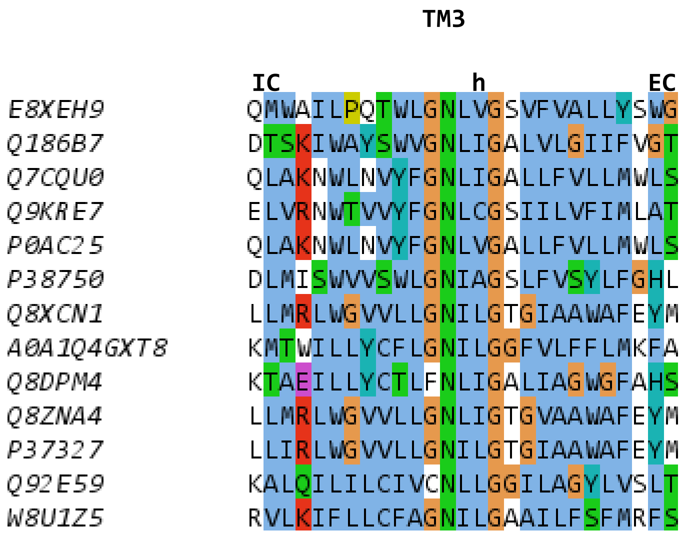
TM3
IC
h
EC
E8XEH9 QMWAILPQTWLGNLVGSVFVALLYSWG
Q186B7 DTSKIWAYSWVGNLIGALVLGIIFVGT
Q7CQU0 QLAKNWLNVYFGNLIGALLFVLLMWLS
Q9KRE7 ELVRNWTVVYFGNLCGSIILVFIMLAT
P0AC25 QLAKNWLNVYFGNLVGALLFVLLMWLS
P38750 DLMISWVVSWLGNIAGSLFVSYLFGHL
Q8XCN1 LLMRLWGVVLLGNILGTGIAAWAFEYM
A0A1Q4GXT8 KMTWILLYCFLGNILGGFVLFFLMKFA
Q8DPM4 KTAEILLYCTLFNLIGALIAGWGFAHS
Q8ZNA4 LLMRLWGVVLLGNLIGTGVAAWAFEYM
P37327 LLIRLWGVVLLGNILGTGIAAWAFEYM
Q92E59 KALQILILCIVCNLLGGILAGYLVSLT
W8U1Z5 RVLKIFLLCFAGNILGAAILFSFMRFS

Foca TM4

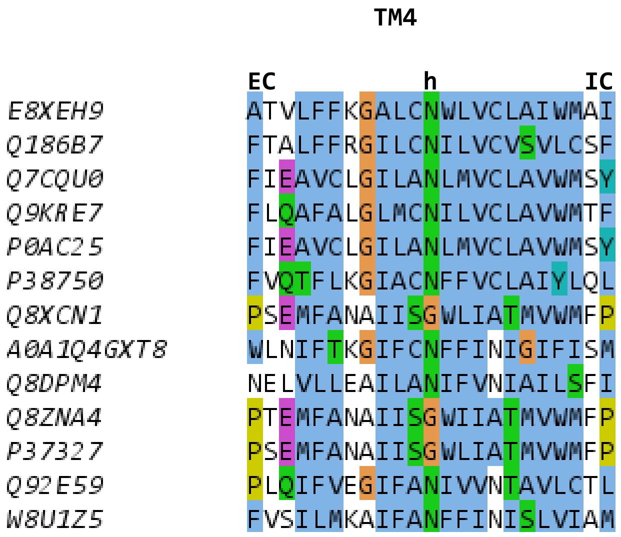
TM4
EC
h
IC
E8XEH9 ATVLFFKGALCNWLVCLAIWMAI
Q186B7 FTALFFRGILCNILVCVSVLCSF
Q7CQU0 FIEAVCLGILANLMVCLAVWMSY
Q9KRE7 FLQAFALGLMCNILVCLAVWMTF
P0AC25 FIEAVCLGILANLMVCLAVWMSY
P38750 FVQTFLKGIACNFFVCLAIYLQL
Q8XCN1 PSEMFANAIISGWLIATMVWMFP
A0A1Q4GXT8 WLNIFTKGIFCNFFINIGIFISM
Q8DPM4 NELVLLEAILANIFVNIAILSFI
Q8ZNA4 PTEMFANAIISGWIIATMVWMFP
P37327 PSEMFANAIISGWLIATMVWMFP
Q92E59 PLQIFVEGIFANIVVNTAVLCTL
W8U1Z5 FVSILMKAIFANFFINISLVIAM

Foca TM5

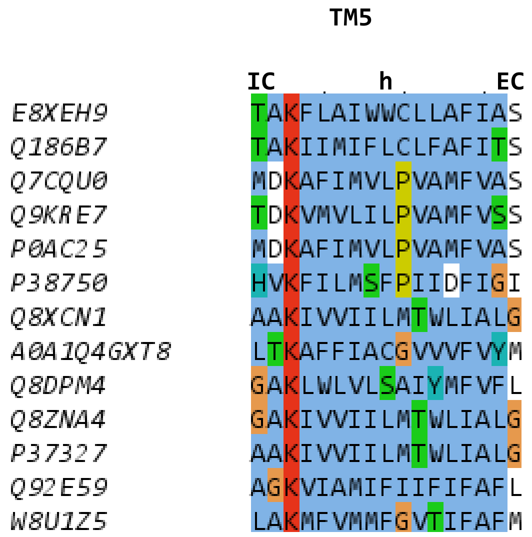

TM5
IC
h
EC
E8XEH9 TAKFLAIWWCLLAFIAS
Q186B7 TAKIIMIFLCLFAFITS
Q7CQU0 MDKAFIMVLPVAMFVAS
Q9KRE7 TDKVMVLILPVAMFVSS
P0AC25 MDKAFIMVLPVAMFVAS
P38750 HVKFILMSFPIIDFIGI
Q8XCN1 AAKIVVIILMTWLIALG
A0A1Q4GXT8 LTKAFFIACGVVVFVYM
Q8DPM4 GAKLWLVLSAIYMFVFL
Q8ZNA4 GAKIVVIILMTWLIALG
P37327 AAKIVVIILMTWLIALG
Q92E59 AGKVIAMIFIIFIFAFL
W8U1Z5 LAKMFVMMFGVTIFAFM

Foca TM5B

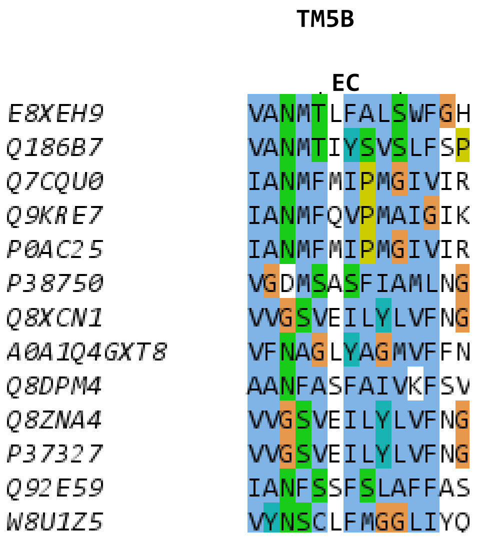
TM5B
EC
E8XEH9 VANMTLFALSWFGH
Q186B7 VANMTIYSVSLFSP
Q7CQU0 IANMFMIPMGIVIR
Q9KRE7 IANMFQVPMAIGIK
P0AC25 IANMFMIPMGIVIR
P38750 VGDMSASFIAMLNG
Q8XCN1 VVGSVEILYLVFNG
A0A1Q4GXT8 VFNAGLYAGMVFFN
Q8DPM4 AANFASFAIVKFSV
Q8ZNA4 VVGSVEILYLVFNG
P37327 VVGSVEILYLVFNG
Q92E59 IANFSSFSLAFFAS
W8U1Z5 VYNSCLFMGGLIYQ

Foca TM6

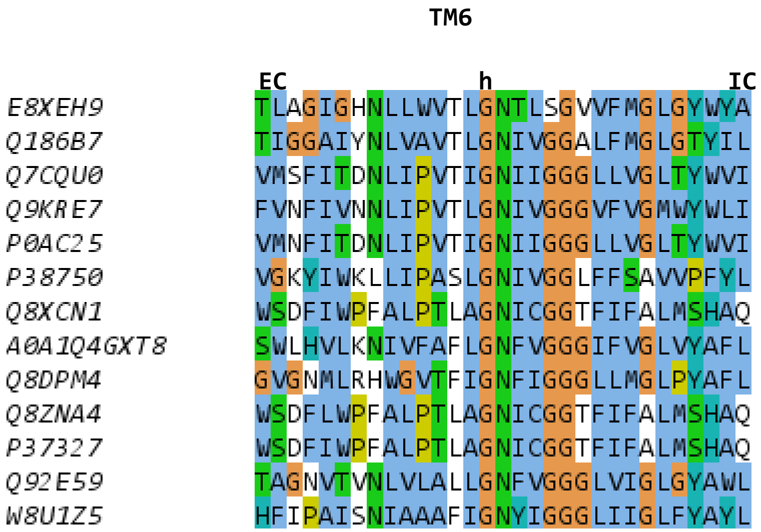
TM6
EC
h
IC
E8XEH9 TLAGIGHNLLWVTLGNTLSGVVFMGLGYWYA
Q186B7 TIGGAIYNLVAVTLGNIVGGALFMGLGTYIL
Q7CQU0 VMSFITDNLIPVTIGNIIGGGLLVGLTYWVI
Q9KRE7 FVNFIVNNLIPVTLGNIVGGGVFVGMWYWLI
P0AC25 VMNFITDNLIPVTIGNIIGGGLLVGLTYWVI
P38750 VGKYIWKLLIPASLGNIVGGLFFSAVVPFYL
Q8XCN1 WSDFIWPFALPTLAGNICGGTFIFALMSHAQ
A0A1Q4GXT8 SWLHVLKNIVFAFLGNFVGGGIFVGLVYAFL
Q8DPM4 GVGNMLRHWGVTFIGNFIGGGLLMGLPYAFL
Q8ZNA4 WSDFLWPFALPTLAGNICGGTFIFALMSHAQ
P37327 WSDFIWPFALPTLAGNICGGTFIFALMSHAQ
Q92E59 TAGNVTVNLVLALLGNFVGGGLVIGLGYAWL
W8U1Z5 HFIPAISNIAAAFIGNYIGGGLIIGLFYAYL

GPCR TM1

```
                TM1

        EC            h             IC
O14842  LPPQLSFGLYVAAFALGFPLNVLAIRGATA
O43613  QYEWVLIAAYVAVFVVALVGNTLVCLAVWR
P07550  VWVVGMGIVMSLIVLAIVFGNVLVITAIAK
P08100  WQFSMLAAYMFLLIVLGFPINFLTLYVTVQ
P08172  FEVVFIVLVAGSLSLVTIIGNILVMVSIKV
P08173  VEMVFIATVTGSLSLVTVVGNILVMLSIKV
P11229  WQVAFIGITTGLLSLATVTGNLLVLISFKV
P21453  NSIKLTSVVFILICCFIILENIFVLLTIWK
P21554  SQQLAIAVLSLTLGTFTVLENLLVLCVILH
P24530  TFKYINTVVSCLVFVLGIIGNSTLLRIIYK
P25024  LNKYVVIIAYALVFLLSLLGNSLVMLVILY
P25116  WLTLFVPSVYTGVFVVSLPLNIMAIVVFIL
P28222  PWKVLLVMLLALITLATTLSNAFVIATVYR
P29274  MGSSVYITVELAIAVLAILGNVLVCWAVWL
P30542  AFQAAYIGIEVLIALVSVPGNVLVIWAVKV
P35367  PQLMPLVVVLSTICLVTVGLNLLVLYAVRS
P35462  RPHAYYALSYCALILAIVFGNGLVCMAVLK
P41143  ALAIAITALYSAVCAVGLLGNVLVMFGIVR
P41145  AIPVIITAVYSVVFVVGLVGNSLVMFVIIR
P41146  GLKVTIVGLYLAVCVGGLLGNCLVMYVILR
P41597  IGAQLLPPLYSLVFIFGFVGNMLVVLILIN
P47900  FQFYYLPAVYILVFIIGFLGNSVAIWMFVF
P50052  KHLDAIPILYYIIFVIGFLVNIVVVTLFCC
P51681  IAARLLPPLYSLVFIFGFVGNMLVILILIN
P51686  FASHFLPPLYWLVFIVGALGNSLVILVYWY
P55085  LTTVFLPIVYTIVFVVGLPSNGMALWVFLF
P61073  FNKIFLPTIYSIIFLTGIVGNGLVILVMGY
Q92633  TVSKLVMGLGITVCIFIMLANLLVMVAIYV
Q9H244  ITQVLFPLLYTVLFFVGLITNGLAMRIFFQ
P20789  YSKVLVTAIYLALFVVGTVGNSVTAFTLAR
P34998  VHYHVAVIINYLGHCISLVALLVAFVLFLR
P43220  EQLLFLYIIYTVGYALSFSALVIASAILLG
P47871  KMYSSFQVMYTVGYSLSLGALLLALAILGG
P41594  RWGDPEPIAAVVFACLGLLATLFVTVVFII
Q13255  EWSNIESIIAIAFSCLGILVTLFVTLIFVL
Q99835  AEHQDMHSYIAAFGAVTGLCTLFTLATFVA
```

GPCR TM2

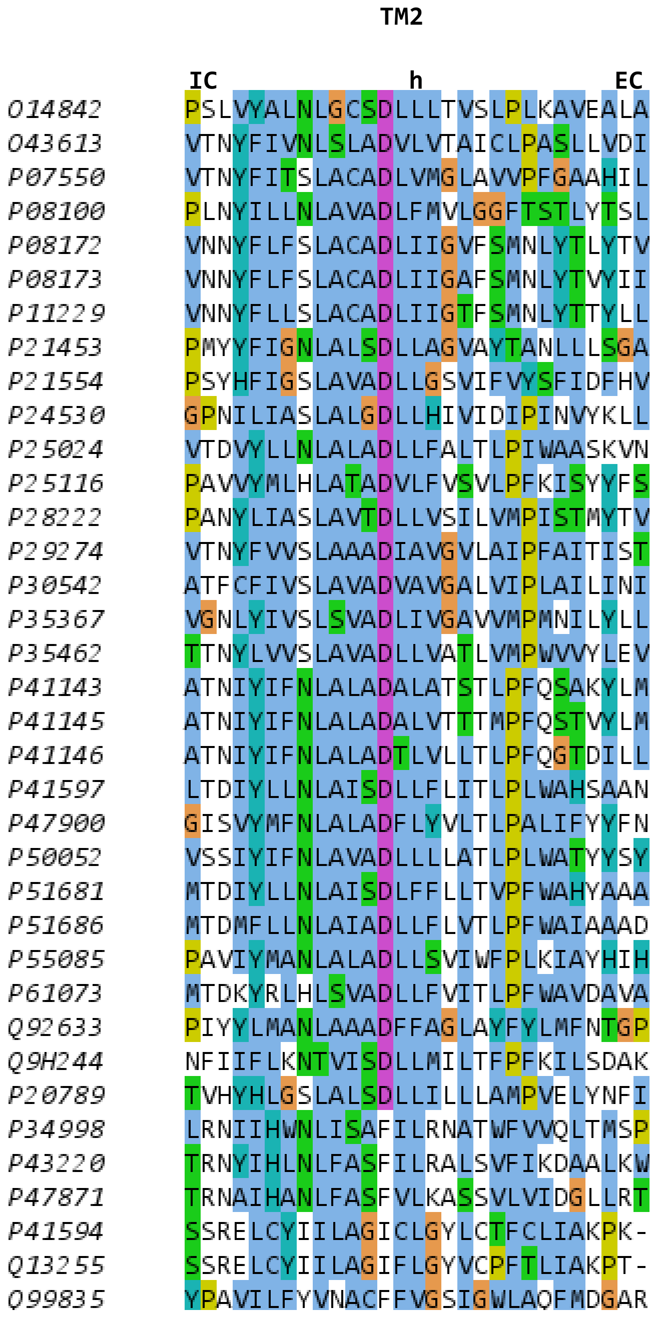
TM2
IC h EC
O14842 PSLVYALNLGCSDLLLTVSLPLKAVEALA
O43613 VTNYFIVNLSLADVLVTAICLPASLLVDI
P07550 VTNYFITSLACADLVMGLAVVPFGAAHIL
P08100 PLNYILLNLAVADLFMVLGGFTSTLYTSL
P08172 VNNYFLFSLACADLIIGVFSMNLYTLYTV
P08173 VNNYFLFSLACADLIIGAFSMNLYTVYII
P11229 VNNYFLLSLACADLIIGTFSMNLYTTYLL
P21453 PMYYFIGNLALSDLLAGVAYTANLLLSGA
P21554 PSYHFIGSLAVADLLGSVIFVYSFIDFHV
P24530 GPNILIASLALGDLLHIVIDIPINVYKLL
P25024 VTDVYLLNLALADLLFALTLPIWAASKVN
P25116 PAVVYMLHLATADVLFVSVLPFKISYYFS
P28222 PANYLIASLAVTDLLVSILVMPISTMYTV
P29274 VTNYFVVSLAAADIAVGVLAIPFAITIST
P30542 ATFCFIVSLAVADVAVGALVIPLAILINI
P35367 VGNLYIVSLSVADLIVGAVVMPMNILYLL
P35462 TTNYLVVSLAVADLLVATLVMPWVVYLEV
P41143 ATNIYIFNLALADALATSTLPFQSAKYLM
P41145 ATNIYIFNLALADALVTTTMPFQSTVYLM
P41146 ATNIYIFNLALADTLVLLTLPFQGTDILL
P41597 LTDIYLLNLAISDLLFLITLPLWAHSAAN
P47900 GISVYMFNLALADFLYVLTLPALIFYYFN
P50052 VSSIYIFNLAVADLLLLATLPLWATYYSY
P51681 MTDIYLLNLAISDLFFLLTVPFWAHYAAA
P51686 MTDMFLLNLAIADLLFLVTLPFWAIAAAD
P55085 PAVIYMANLALADLLSVIWFPLKIAYHIH
P61073 MTDKYRLHLSVADLLFVITLPFWAVDAVA
Q92633 PIYYLMANLAAADFFAGLAYFYLMFNTGP
Q9H244 NFIIFLKNTVISDLLMILTFPFKILSDAK
P20789 TVHYHLGSLALSDLLILLLAMPVELYNFI
P34998 LRNIIHWNLISAFILRNATWFVVQLTMSP
P43220 TRNYIHLNLFASFILRALSVFIKDAALKW
P47871 TRNAIHANLFASFVLKASSVLVIDGLLRT
P41594 SSRELCYIILAGICLGYLCTFCLIAKPK-
Q13255 SSRELCYIILAGIFLGYVCPFTLIAKPT-
Q99835 YPAVILFYVNACFFVGSIGWLAQFMDGAR

GPCR TM3

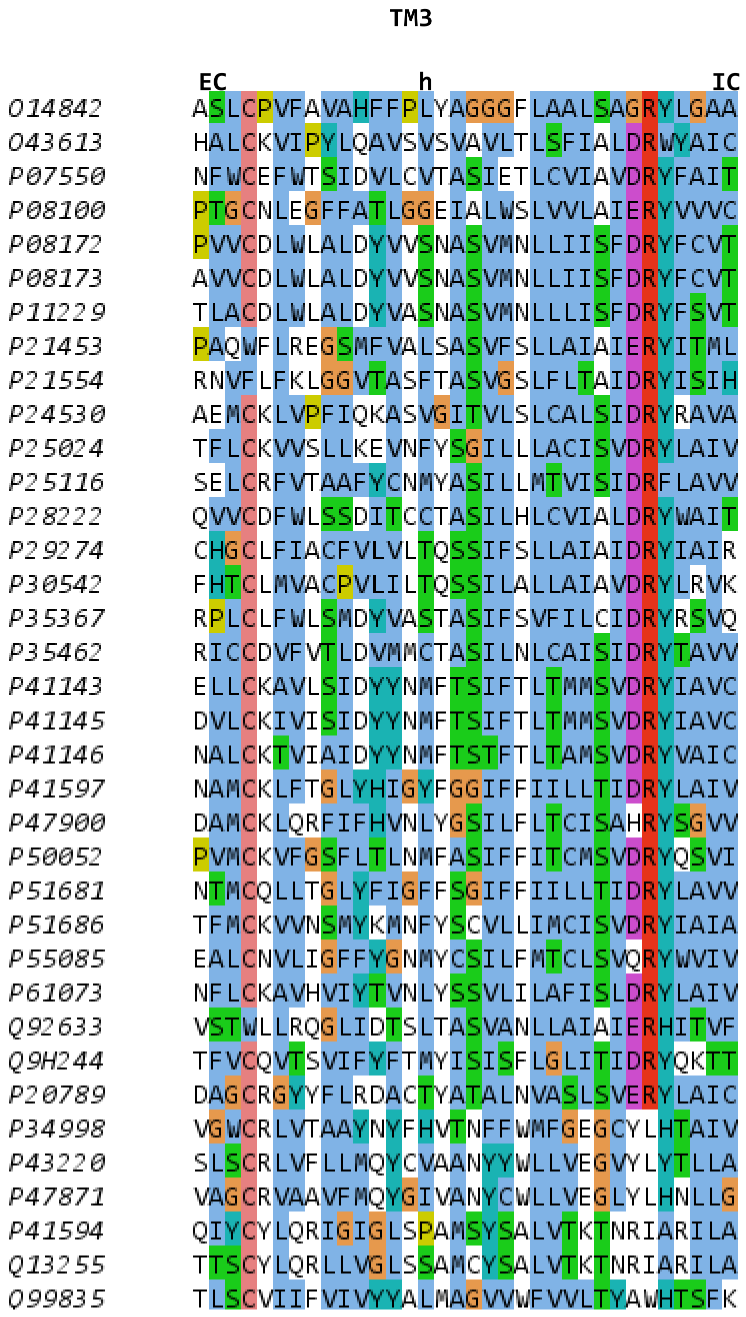
TM3
EC
h
IC
O14842 ASLCPVFAVAHFFPLYAGGGFLAALSAGRYLGAA
O43613 HALCKVIPYLQAVSVSVAVLTLSFIALDRWYAIC
P07550 NFWCEFWTSIDVLCVTASIETLCVIAVDRYFAIT
P08100 PTGCNLEGFFATLGGEIALWSLVVLAIERYVVVC
P08172 PVVCDLWLALDYVVSNASVMNLLIISFDRYFCVT
P08173 AVVCDLWLALDYVVSNASVMNLLIISFDRYFCVT
P11229 TLACDLWLALDYVASNASVMNLLLISFDRYFSVT
P21453 PAQWFLREGSMFVALSASVFSLLAIAIERYITML
P21554 RNVFLFKLGGVTASFTASVGSLFLTAIDRYISIH
P24530 AEMCKLVPFIQKASVGITVLSLCALSIDRYRAVA
P25024 TFLCKVVSLLKEVNFYSGILLLACISVDRYLAIV
P25116 SELCRFVTAAFYCNMYASILLMTVISIDRFLAVV
P28222 QVVCDFWLSSDITCCTASILHLCVIALDRYWAIT
P29274 CHGCLFIACFVLVLTQSSIFSLLAIAIDRYIAIR
P30542 FHTCLMVACPVLILTQSSILALLAIAVDRYLRVK
P35367 RPLCLFWLSMDYVASTASIFSVFILCIDRYRSVQ
P35462 RICCDVFVTLDVMMCTASILNLCAISIDRYTAVV
P41143 ELLCKAVLSIDYYNMFTSIFTLTMMSVDRYIAVC
P41145 DVLCKIVISIDYYNMFTSIFTLTMMSVDRYIAVC
P41146 NALCKTVIAIDYYNMFTSTFTLTAMSVDRYVAIC
P41597 NAMCKLFTGLYHIGYFGGIFFIILLTIDRYLAIV
P47900 DAMCKLQRFIFHVNLYGSILFLTCISAHRYSGVV
P50052 PVMCKVFGSFLTLNMFASIFFITCMSVDRYQSVI
P51681 NTMCQLLTGLYFIGFFSGIFFIILLTIDRYLAVV
P51686 TFMCKVVNSMYKMNFYSCVLLIMCISVDRYIAIA
P55085 EALCNVLIGFFYGNMYCSILFMTCLSVQRYWVIV
P61073 NFLCKAVHVIYTVNLYSSVLILAFISLDRYLAIV
Q92633 VSTWLLRQGLIDTSLTASVANLLAIAIERHITVF
Q9H244 TFVCQVTSVIFYFTMYISISFLGLITIDRYQKTT
P20789 DAGCRGYYFLRDACTYATALNVASLSVERYLAIC
P34998 VGWCRLVTAAYNYFHVTNFFWMFGEGCYLHTAIV
P43220 SLSCRLVFLLMQYCVAANYYWLLVEGVYLYTLLA
P47871 VAGCRVAAVFMQYGIVANYCWLLVEGLYLHNLLG
P41594 QIYCYLQRIGIGLSPAMSYSALVTKTNRIARILA
Q13255 TTSCYLQRLLVGLSSAMCYSALVTKTNRIARILA
Q99835 TLSCVIIFVIVYYALMAGVVWFVVLTYAWHTSFK

GPCR TM4

TM4

IC h EC

O14842	PCYSWGVCAAIWALVLCHLGLVFGL
O43613	ARRARGSILGIWAVSLAIMVPQAAV
P07550	KNKARVIILMVWIVSGLTSFLPIQM
P08100	ENHAIMGVAFTWVMALACAAPPLAG
P08172	TKMAGMMIAAAWVLSFILWAPAILF
P08173	TKMAGLMIAAAWVLSFVLWAPAILF
P11229	PRRAALMIGLAWLVSFVLWAPAILF
P21453	NFRLFLLISACWVISLILGGLPIMG
P21554	RPKAVVAFCLMWTIAIVIAVLPLLG
P24530	PKWTAVEIVLIWVVSVVLAVPEAIG
P25024	RHLVKFVCLGCWGLSMNLSLPFFLF
P25116	LGRASFTCLAIWALAIAGVVPLLLK
P28222	PKRAAVMIALVWVFSISISLPPFFW
P29274	GTRAKGIIAICWVLSFAIGLTPMLG
P30542	PRRAAVAIAGCWILSFVVGLTPMFG
P35367	KTRASATILGAWFLSFLWVIPILGW
P35462	CRRVALMITAVWVLAFAVSCPLLFG
P41143	PAKAKLINICIWVLASGVGVPIMVM
P41145	PLKAKIINICIWLLSSSVGISAIVL
P41146	SSKAQAVNVAIWALASVVGVPVAIM
P41597	VTFGVVTSVITWLVAVFASVPGIIF
P47900	KKNAICISVLVWLIVVVAISPILFY
P50052	PWQASYIVPLVWCMACLSSLPTFYF
P51681	VTFGVVTSVITWVVAVFASLPGIIF
P51686	LLYSKMVCFTIWVLAAALCIPEILY
P55085	ANIAIGISLAIWLLILLVTIPLYVV
P61073	LLAEKVVYVGVWIPALLLTIPDFIF
Q92633	NRRVVVVIVVIWTMAIVMGAIPSVG
Q9H244	LLGAKILSVVIWAFMFLLSLPNMIL
P20789	RSRTKKFISAIWLASALLAIPMLFT
P34998	RLRKWMFICIGWGVPFPIIVAWAIG
P43220	QWIFRLYVSIGWGVPLLFVVPWGIV
P47871	RSFFSLYLGIGWGAPMLFVVPWAVV
P41594	ACAQLVIAFILICIQLGIIVALFIM
Q13255	AWAQVIIASILISVQLTLVVTLIIM
Q99835	SGKTSYFHLLTWSLPFVLTVAILAV

GPCR TM5

TM5

```
        EC                   h             IC
O14842  SAGPARFSLSLLLFFLPLAITAFCYVGCLRALAR
O43613  YPKIYHSCFFIVTYLAPLGLMAMAYFQIFRKLWG
P07550  TNQAYAIASSIVSFYVPLVIMVFVYSRVFQEAKR
P08100  NNESFVIYMFVVHFTIPMIIIFFCYGQLVFTVKE
P08172  SNAAVTFGTAIAAFYLPVIIMTVLYWHISRASKS
P08173  SNPAVTFGTAIAAFYLPVVIMTVLYIHISLASRS
P11229  SQPIITFGTAMAAFYLPVTVMCTLYWRIYRETEN
P21453  LYHKHYILFCTTVFTLLLLSIVILYCRIYSLVRT
P21554  HIDETYLMFWIGVTSVLLLFIVYAYMYILWKAHS
P24530  YKTAKDWWLFSFYFCLPLAITAFFYTLMTCEMLR
P25024  WRMVLRILPHTFGFIVPLFVMLFCYGFTLRTLFK
P25116  YYAYYFSAFSAVFFFVPLIISTVCYVSIIRCLSS
P28222  DHILYTVYSTVGAFYFPTLLLIALYGRIYVEARS
P29274  PMNYMVYFNFFACVLVPLLLMLGVYLRIFLAARR
P30542  SMEYMVYFNFFVWVLPPLLLMVLIYLEVFYLIRK
P35367  DVTWFKVMTAIINFYLPTLLMLWFYAKIYKAVRQ
P35462  SNPDFVIYSSVVSFYLPFGVTVLVYARIYVVLKQ
P41143  WDTVTKICVFLFAFVVPILIITVCYGLMLLRLRS
P41145  WDLFMKICVFIFAFVIPVLIIIVCYTLMILRLKS
P41146  WGPVFAICIFLFSFIVPVLVISVCYSLMIRRLRG
P41597  WNNFHTIMRNILGLVLPLLIMVICYSGILKTLLR
P47900  SYFIYSMCTTVAMFCVPLVLILGCYGLIVRALIY
P50052  WSAGIALMKNILGFIIPLIFIATCYFGIRKHLLK
P51681  WKNFQTLKIVILGLVLPLLVMVICYSGILKTLLR
P51686  LKSAVLTLKVILGFFLPFVVMACCYTIIIHTLIQ
P55085  DMFNYFLSLAIGVFLFPAFLTASAYVLMIRMLRS
P61073  WVVVFQFQHIMVGLILPGIVILSCYCIIISKLSH
Q92633  LYSDSYLVFWAIFNLVTFVVMVVLYAHIFGYVRQ
Q9H244  VWHEIVNYICQVIFWINFLIVIVCYTLITKELYR
P20789  TVKVVIQVNTFMSFLFPMLVISILNTVIANKLTV
P34998  RPGVYTDYIYQGPMILVLLINFIFLFNIVRILMT
P43220  NSNMNYWLIIRLPILFAIGVNFLIFVRVICIVVS
P47871  NDNMGFWWILRFPVFLAILINFFIFVRIVQLLVA
P41594  NTTNLGVVTPLGYNGLLILSCTFYAFKTRNVPA-
Q13255  NTSNLGVVAPLGYNGLLIMSCTYYAFKTRNVPA-
Q99835  KNYRYRAGFVLAPIGLVLIVGGYFLIRGVMTLFS
```

GPCR TM6

```
                 TM6

          IC                  h         EC
O14842    RRKLRAAWVAGGALLTLLLCVGPYNASNVASFL
O43613    RARRKTAKMLMVVLLVFALCYLPISVLNVLKRV
P07550    LKEHKALKTLGIIMGTFTLCWLPFFIVNIVHVI
P08100    KAEKEVTRMVIIMVIAFLICWVPYASVAFYIFT
P08172    SREKKVTRTILAILLAFIITWAPYNVMVLINTF
P08173    ARERKVTRTIFAILLAFILTWTPYNVMVLVNTF
P11229    VKEKKAARTLSAILLAFILTWTPYNIMVLVSTF
P21453    EKSLALLKTVIIVLSVFIACWAPLFILLLLDVG
P21554    RMDIRLAKTLVLILVVLIICWGPLLAIMVYDVF
P24530    KQRREVAKTVFCLVLVFALCWLPLHLSRILKLT
P25024    GQKHRAMRVIFAVVLIFLLCWLPYNLVLLADTL
P25116    SKKSRALFLSAAVFCIFIICFGPTNVLLIAHYS
P28222    ARERKATKTLGIILGAFIVCWLPFFIISLVMPI
P29274    QKEVHAAKSLAIIVGLFALCWLPLHIINCFTFF
P30542    GKELKIAKSLALILFLFALSWLPLHILNCITLF
P35367    NRERKAAKQLGFIMAAFILCWIPYFIFFMVIAF
P35462    LREKKATQMVAIVLGAFIVCWLPFFLTHVLNTH
P41143    RSLRRITRMVLVVVGAFVVCWAPIHIFVIVWTL
P41145    RNLRRITRLVLVVVAVFVVCWTPIHIFILVEAL
P41146    RNLRRITRLVLVVVAVFVGCWTPVQVFVLAQGL
P41597    KKRHRAVRVIFTIMIVYFLFWTPYNIVILLNTF
P47900    PLRRKSIYLVIIVLTVFAVSYIPFHVMKTMNLR
P50052    ITRDQVLKMAAAVVLAFIICWLPFHVLTFLDAL
P51681    KKRHRAVRLIFTIMIVYFLFWAPYNIVLLLNTF
P51686    SSKHKALKVTITVLTVFVLSQFPYNCILLVQTI
P55085    KKRKRAIKLIVTVLAMYLICFTPSNLLLVVHYF
P61073    HQKRKALKTTVILILAFFACWLPYYIGISIDSF
Q92633    DTMMSLLKTVVIVLGAFIICWTPGLVLLLLDVC
Q9H244    VPRKKVNVKVFIIIAVFFICFVPFHFARIPYTL
P20789    QALRHGVLVLRAVVIAFVVCWLPYHVRRLMFCY
P34998    IQYRKAVKATLVLLPLLGITYMLFFVNPGEDEV
P43220    DIKCRLAKSTLTLIPLLGTHEVIFAFVMDEHAR
P47871    DYKFRLAKSTLTLIPLLGVHEVVFAFVTDEHAQ
P41594    --NFNEAKYIAFTMYTTCIIWLAFVPIYFGSNY
Q13255    --NFNEAKYIAFTMYTTCIIWLAFVPIYFGSNY
Q99835    SKINETMLRLGIFGFLAFGFVLITFSCHFYDFF
```

GPCR TM7

TM7

	EC h IC
O14842	LGGSWRKLGLITGAWSVVLNPLVTGYL
O43613	AVYACFTFSHWLVYANSAANPIIYNFL
P07550	IRKEVYILLNWIGYVNSGFNPLIYCRS
P08100	FGPIFMTIPAFFAKSAAIYNPVIYIMM
P08172	IPNTVWTIGYWLCYINSTINPACYALC
P08173	IPDTVWSIGYWLCYVNSTINPACYALC
P11229	VPETLWELGYWLCYVNSTINPMCYALC
P21453	DILFRAEYFLVLAVLNSGTNPIIYTLT
P21554	LIKTVFAFCSMLCLLNSTVNPIIYALR
P24530	FLLVLDYIGINMASLNSCINPIALYLV
P25024	NIGRALDATEILGFLHSCLNPIIYAFI
P25116	AAYFAYLLCVCVSSISCCIDPLIYYYA
P28222	FHLAIFDFFTWLGYLNSLINPIIYTMS
P29274	APLWLMYLAIVLSHTNSVVNPFIYAYR
P30542	KPSILTYIAIFLTHGNSAMNPIVYAFR
P35367	CNEHLHMFTIWLGYINSTLNPLIYPLC
P35462	VSPELYSATTWLGYVNSALNPVIYTTF
P41143	LVVAALHLCIALGYANSSLNPVLYAFL
P41145	AALSSYYFCIALGYTNSSLNPILYAFL
P41146	TAVAILRFCTALGYVNSCLNPILYAFL
P41597	QLDQATQVTETLGMTHCCINPIIYAFV
P47900	RVYATYQVTRGLASLNSCVDPILYFLA
P50052	VIDLALPFAILLGFTNSCVNPFLYCFV
P51681	RLDQAMQVTETLGMTHCCINPIIYAFV
P51686	NIDICFQVTQTIAFFHSCLNPVLYVFV
P55085	HVYALYIVALCLSTLNSCIDPFVYYFV
P61073	TVHKWISITEALAFFHCCLNPILYAFL
Q92633	DVLAYEKFFLLLAEFNSAMNPIIYSYR
Q9H244	TLFYVKESTLWLTSLNACLDPFIYFFL
P20789	FYHYFYMLTNALFYVSSAINPILYNLV
P34998	SRVVFIYFNSFLESFQGFFVSVFYCFL
P43220	LRFIKLFTELSFTSFQGLMVAILYCFV
P47871	LRSAKLFFDLFLSSFQGLLVAVLYCFL
P41594	----KIITMCFSVSLSATVALGCMFVP
Q13255	----KIITTCFAVSLSVTVALGCMFTP
Q99835	PSLLVEKINLFAMFGTGIAMSTWVWTK

MFS TM1

```
                          TM1

        IC                 h                 EC
P08194  YRRLRWQIFLGIFFGYAAYYLVRKNFALAMPYLVEQG
P02920  MYYLKNTNFWMFGLFFFFYFFIMGAYFPFFPIWLHDI
P76350  SLSRARRAALGSFAGAVVDWYDFLLYGITAALVFNRE
P0AEX3  DTRRRIWAIVGASSGNLVEWFDFYVYSFCSLYFAHIF
Q5HRH0  DGNNAKKTVIATGIGNAMEWFDFGLYSYLAVIISKNF
O51798  GSPQQKKTFWACYSGWALDSFDMQMFSFLLPALTLTW
P0C0L7  DDGKLRKAITAASLGNAMEWFDFGVYGFVAYALGKVF
P37643  PINSRNKVLVASLIGTAIEFFDFYIYATAAVIVFPHI
P71369  VNSYGWKALIGSAVGYGMDGFDLLILGFMLSAISADL
P94131  GSHTWKIAFLFAFLALLVDGADLMLLSYSLNSIKAEF
P11166  VGGAVLGSLQFGYNTGVINAPQKVIEEFYNQTWVHRY
P0AGF4  NSSYIFSITLVATLGGLLFGYDTAVISGTVESLNTVF
P11551  GQSRSYIIPFALLCSLFFLWAVANNLNDILLPQFQQA
```

MFS TM2

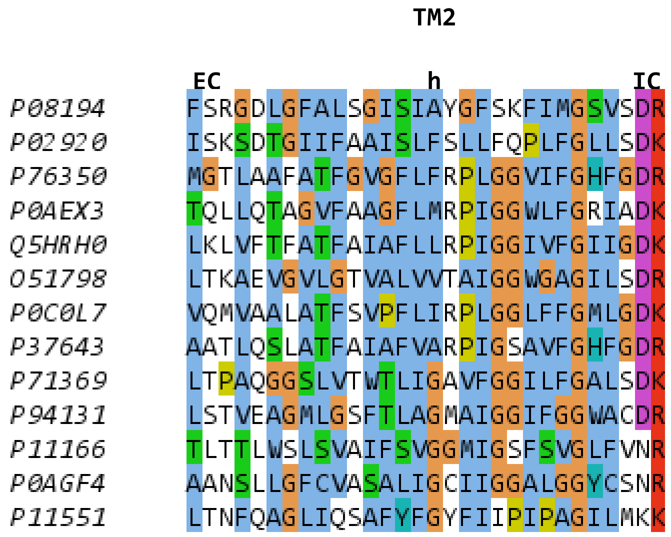

TM2
EC
h
IC
P08194 FSRGDLGFALSGISIAYGFSKFIMGSVSDR
P02920 ISKSDTGIIFAAISLFSLLFQPLFGLLSDK
P76350 MGTLAAFATFGVGFLFRPLGGVIFGHFGDR
P0AEX3 TQLLQTAGVFAAGFLMRPIGGWLFGRIADK
Q5HRH0 LKLVFTFATFAIAFLLRPIGGIVFGIIGDK
O51798 LTKAEVGVLGTVALVVTAIGGWGAGILSDR
P0C0L7 VQMVAALATFSVPFLIRPLGGLFFGMLGDK
P37643 AATLQSLATFAIAFVARPIGSAVFGHFGDR
P71369 LTPAQGGSLVTWTLIGAVFGGILFGALSDK
P94131 LSTVEAGMLGSFTLAGMAIGGIFGGWACDR
P11166 TLTTLWSLSVAIFSVGGMIGSFSVGLFVNR
P0AGF4 AANSLLGFCVASALIGCIIGGALGGYCSNR
P11551 LTNFQAGLIQSAFYFGYFIIPIPAGILMKK

MFS TM3

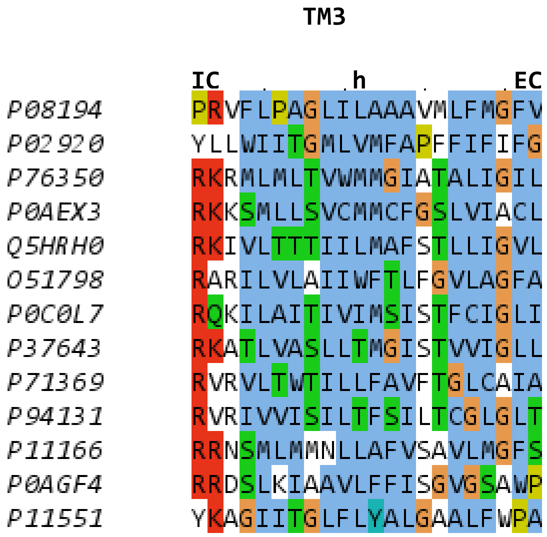
TM3
IC
h
EC
P08194 PRVFLPAGLILAAAVMLFMGFV
P02920 YLLWIITGMLVMFAPFFIFIFG
P76350 RKRMLMLTVWMMGIATALIGIL
P0AEX3 RKKSMLLSVCMMCFGSLVIACL
Q5HRH0 RKIVLTTTIILMAFSTLLIGVL
O51798 RARILVLAIIWFTLFGVLAGFA
P0C0L7 RQKILAITIVIMSISTFCIGLI
P37643 RKATLVASLLTMGISTVVIGLL
P71369 RVRVLTWTILLFAVFTGLCAIA
P94131 RVRIVVISILTFSILTCGLGLT
P11166 RRNSMLMMNLLAFVSAVLMGFS
P0AGF4 RRDSLKIAAVLFFISGVGSAWP
P11551 YKAGIITGLFLYALGAALFWPA

MFS TM4

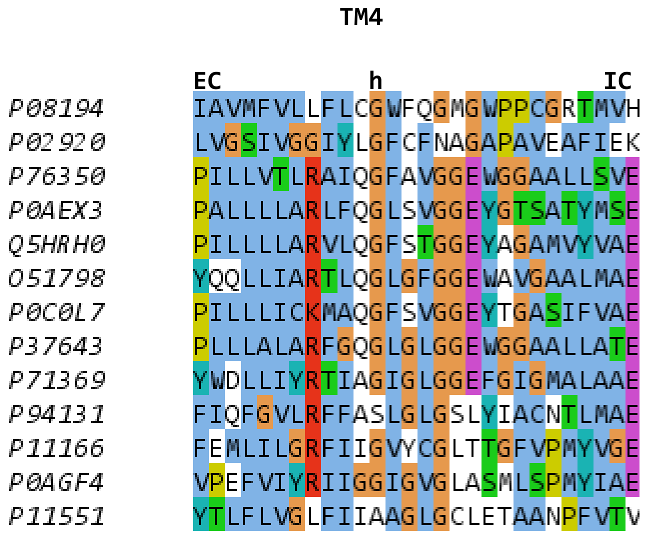

TM4
EC
h
IC
P08194 IAVMFVLLFLCGWFQGMGWPPCGRTMVH
P02920 LVGSIVGGIYLGFCFNAGAPAVEAFIEK
P76350 PILLVTLRAIQGFAVGGEWGGAALLSVE
P0AEX3 PALLLLARLFQGLSVGGEYGTSATYMSE
Q5HRH0 PILLLLARVLQGFSTGGEYAGAMVYVAE
O51798 YQQLLIARTLQGLGFGGEWAVGAALMAE
P0C0L7 PILLLICKMAQGFSVGGEYTGASIFVAE
P37643 PLLLALARFGQGLGLGGEWGGAALLATE
P71369 YWDLLIYRTIAGIGLGGEFGIGMALAAE
P94131 FIQFGVLRFFASLGLGSLYIACNTLMAE
P11166 FEMLILGRFIIGVYCGLTTGFVPMYVGE
P0AGF4 VPEFVIYRIIGGIGVGLASMLSPMYIAE
P11551 YTLFLVGLFIIAAGLGCLETAANPFVTV

MFS TM5

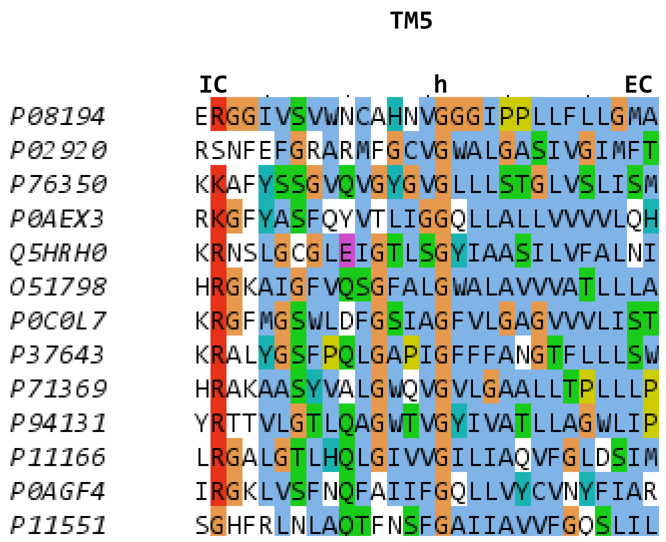
TM5
IC
h
EC
P08194 ERGGIVSVWNCAHNVGGGIPPLLFLLGMA
P02920 RSNFEFGRARMFGCVGWALGASIVGIMFT
P76350 KKAFYSSGVQVGYGVGLLLSTGLVSLISM
P0AEX3 RKGFYASFQYVTLIGGQLLALLVVVVLQH
Q5HRH0 KRNSLGCGLEIGTLSGYIAASILVFALNI
O51798 HRGKAIGFVQSGFALGWALAVVVATLLLA
P0C0L7 KRGFMGSWLDFGSIAGFVLGAGVVVLIST
P37643 KRALYGSFPQLGAPIGFFFANGTFLLLSW
P71369 HRAKAASYVALGWQVGVLGAALLTPLLLP
P94131 YRTTVLGTLQAGWTVGYIVATLLAGWLIP
P11166 LRGALGTLHQLGIVVGILIAQVFGLDSIM
P0AGF4 IRGKLVSFNQFAIIFGQLLVYCVNYFIAR
P11551 SGHFRLNLAQTFNSFGAIIAVVFGQSLIL

MFS TM6

```
                TM6

          EC          h         IC
P08194    HAALYMPAFCAILVALFAFAMMRD
P02920    INNQFVFWLGSGCALILAVLLFFA
P76350    WGWRIPFLFSIVLVLGALWVRNGM
P0AEX3    ALREWGWRIPFALGAVLAVVALWL
Q5HRH0    WGWRIPFLLGMFLGLFGLYLRRKL
O51798    MAWRVAFWSGIIPALIVLFIRRHV
P0C0L7    WGWRIPFFIALPLGIIGLYLRHAL
P37643    WGWRVPFIFSAVLVIIGLYVRVSL
P71369    IGWRGMFLVGIFPAFVAWFLRSHL
P94131    HGWRVLFYVAIIPVLMAVLMHFFV
P11166    DLWPLLLSIIFIPALLQCIVLPFC
P0AGF4    DGWRYMFASECIPALLFLMLLYTV
P11551    LSVQTPYMIIVAIVLLVALLIMLT
```

MFS TM7

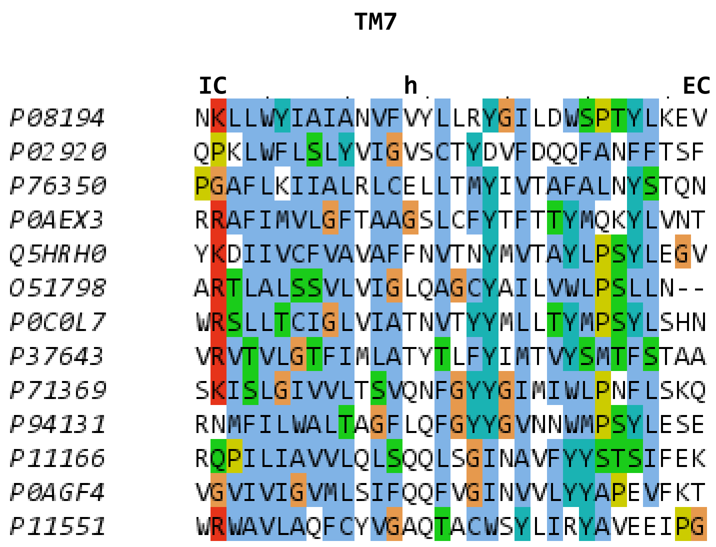

TM7
IC
h
EC
P08194 NKLLWYIAIANVFVYLLRYGILDWSPTYLKEV
P02920 QPKLWFLSLYVIGVSCTYDVFDQQFANFFTSF
P76350 PGAFLKIIALRLCELLTMYIVTAFALNYSTQN
P0AEX3 RRAFIMVLGFTAAGSLCFYTFTTYMQKYLVNT
Q5HRH0 YKDIIVCFVAVAFFNVTNYMVTAYLPSYLEGV
O51798 ARTLALSSVLVIGLQAGCYAILVWLPSLLN--
P0C0L7 WRSLLTCIGLVIATNVTYYMLLTYMPSYLSHN
P37643 VRVTVLGTFIMLATYTLFYIMTVYSMTFSTAA
P71369 SKISLGIVVLTSVQNFGYYGIMIWLPNFLSKQ
P94131 RNMFILWALTAGFLQFGYYGVNNWMPSYLESE
P11166 RQPILIAVVLQLSQQLSGINAVFYYSTSIFEK
P0AGF4 VGVIVIGVMLSIFQQFVGINVVLYYAPEVFKT
P11551 WRWAVLAQFCYVGAQTACWSYLIRYAVEEIPG

MFS TM8

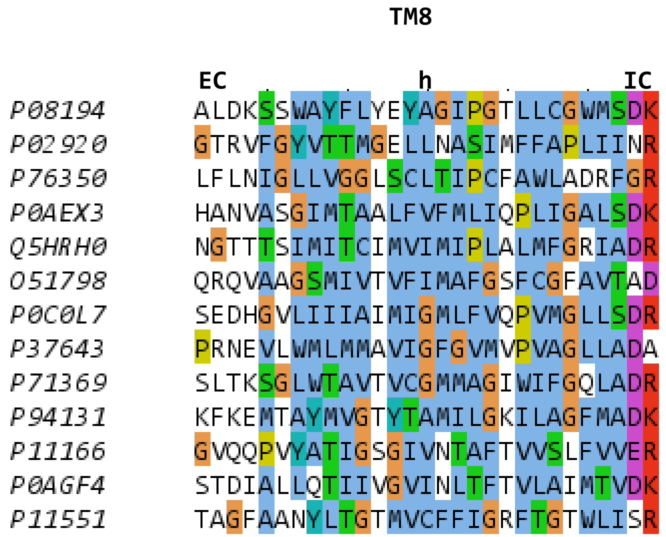
TM8
EC
h
IC
P08194 ALDKSSWAYFLYEYAGIPGTLLCGWMSDK
P02920 GTRVFGYVTTMGELLNASIMFFAPLIINR
P76350 LFLNIGLLVGGLSCLTIPCFAWLADRFGR
P0AEX3 HANVASGIMTAALFVFMLIQPLIGALSDK
Q5HRH0 NGTTTSIMITCIMVIMIPLALMFGRIADR
O51798 QRQVAAGSMIVTVFIMAFGSFCGFAVTAD
P0C0L7 SEDHGVLIIIAIMIGMLFVQPVMGLLSDR
P37643 PRNEVLWMLMMAVIGFGVMVPVAGLLADA
P71369 SLTKSGLWTAVTVCGMMAGIWIFGQLADR
P94131 KFKEMTAYMVGTYTAMILGKILAGFMADK
P11166 GVQQPVYATIGSGIVNTAFTVVSLFVVER
P0AGF4 STDIALLQTIIVGVINLTFTVLAIMTVDK
P11551 TAGFAANYLTGTMVCFFIGRFTGTWLISR

MFS TM9

```
                TM9

        IC        h        EC
P08194  RGATGVFFMTLVTIATIVYWM
P02920  GKNALLLAGTIMSVRIIGSSF
P76350  -RRVYITGTLIGTLSAFPFFM
P0AEX3  RRTSMLCFGSLAAIFTVPILS
Q5HRH0  EKKVFLIGLGGLILLSVVAFS
O51798  TLILLSVCAWIVTVSYMLLPL
P0C0L7  VLLGSVALFVLAIPAFILINS
P37643  TKSMVIITTLIILFALFAFNP
P71369  RKPSFLLFQLGAVISIVVYSQ
P94131  RRFTYAFGAIGTAIFLPLIVF
P11166  GLAGMAGCAILMTIALALLEQ
P0AGF4  RKPLQIIGALGMAIGMFSLGT
P11551  PHKVLAAYALIAMALCLISAF
```

MFS TM10

```
                 TM10

         EC            h             IC
P08194   GNPTVDMICMIVIGFLIYGPVMLIGLHALE
P02920   ATSALEVVILKTLHMFEVPFLLVGCFKYIT
P76350   LEAQSIFWIVFFSIMLANIAHDMVVCVQQP
P0AEX3   YAAFGLVMCALLIVSFYTSISGILKAEMFP
Q5HRH0   FFVSIGVLILGFFLSTYEATMPGSLPTMFY
O51798   TLTAILGFLVGFSAIGMFAALGPFLSELFP
P0C0L7   --NVIGLIFAGLLMLAVILNCFTGVMASTL
P37643   ILVFAFLLLGLSLMGLTFGPMGALLPELFP
P71369   DIMLLAGAFLGMFVNGMLGGYGALMAEAYP
P94131   DNILYLLVIFGFLYGIPYGVNATYMTESFP
P11166   MSYLSIVAIFGFVAFFEVGPGPIPWFIVAE
P0AGF4   GIVALLSMLFYVAAFAMSWGPVCWVLLSEI
P11551   HVGLIALTLCSAFMSIQYPTIFSLGIKNLG
```

MFS TM11

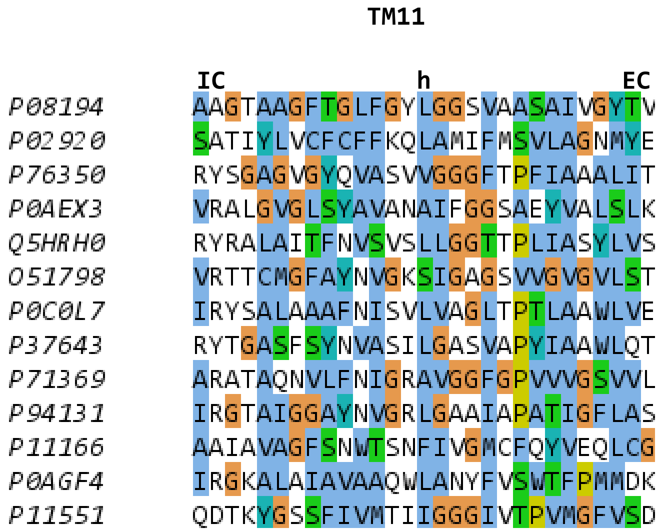
TM11
IC h EC
P08194 AAGTAAGFTGLFGYLGGSVAASAIVGYTV
P02920 SATIYLVCFCFFKQLAMIFMSVLAGNMYE
P76350 RYSGAGVGYQVASVVGGGFTPFIAAALIT
P0AEX3 VRALGVGLSYAVANAIFGGSAEYVALSLK
Q5HRH0 RYRALAITFNVSVSLLGGTTPLIASYLVS
O51798 VRTTCMGFAYNVGKSIGAGSVVGVGVLST
P0C0L7 IRYSALAAAFNISVLVAGLTPTLAAWLVE
P37643 RYTGASFSYNVASILGASVAPYIAAWLQT
P71369 ARATAQNVLFNIGRAVGGFGPVVVGSVVL
P94131 IRGTAIGGAYNVGRLGAAIAPATIGFLAS
P11166 AAIAVAGFSNWTSNFIVGMCFQYVEQLCG
P0AGF4 IRGKALAIAVAAQWLANYFVSWTFPMMDK
P11551 QDTKYGSSFIVMTIIGGGIVTPVMGFVSD

MFS TM12

TM12

```
          EC          h           IC
P08194    WDGGFMVMIGGSILAVILLIVVMIGEK
P02920    FQGAYLVLGLVALGFTLISVFTLSGPG
P76350    HSVAIYLLAGCLISAMTALLMKDSQRA
P0AEX3    ETAFFWYVTLMAVVAFLVSLMLHRKGK
Q5HRH0    PLTPAYYLTVISIIGFIVIALLHKSTA
O51798    ANAMGTFCLVAYAFAVFGIMLLPETRG
P0C0L7    LMMPAYYLMVVAVVGLITGVTMKETAN
P37643    LGAVGLYLAAMAGLTLIALLLTHETRH
P71369    FQTAIALLAIIYVIDMLATIFLIPELK
P94131    IGLGFVVMGAAYFICGVIPALFIKEKQ
P11166    --YVFIIFTVLLVLFFIFTYFKVPETK
P0AGF4    HFHNGFSYWIYGCMGVLAALFMWKFVP
P11551    IPTAELIPALCFAVIFIFARFRSQTAT
```

PNuC TM0

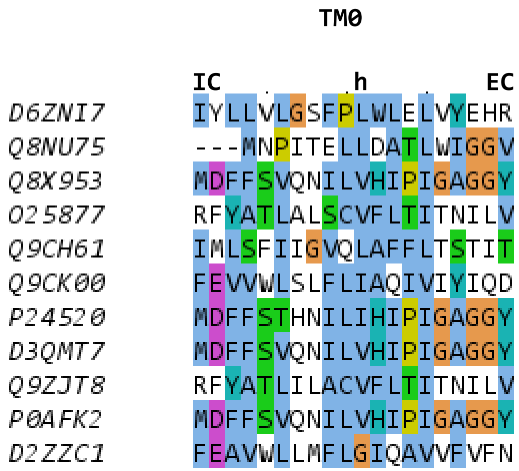
TM0
IC
h
EC
D6ZNI7 IYLLVLGSFPLWLELVYEHR
Q8NU75 ---MNPITELLDATLWIGGV
Q8X953 MDFFSVQNILVHIPIGAGGY
O25877 RFYATLALSCVFLTITNILV
Q9CH61 IMLSFIIGVQLAFFLTSTIT
Q9CK00 FEVVWLSLFLIAQIVIYIQD
P24520 MDFFSTHNILIHIPIGAGGY
D3QMT7 MDFFSVQNILVHIPIGAGGY
Q9ZJT8 RFYATLILACVFLTITNILV
P0AFK2 MDFFSVQNILVHIPIGAGGY
D2ZZC1 FEAVWLLMFLGIQAVVFVFN

PNuC TM1

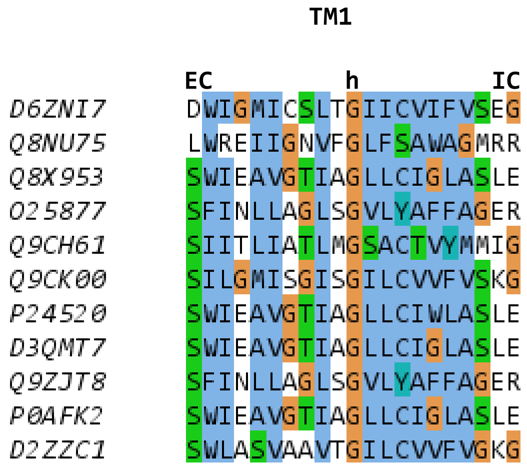
TM1
EC
h
IC
D6ZNI7 DWIGMICSLTGIICVIFVSEG
Q8NU75 LWREIIGNVFGLFSAWAGMRR
Q8X953 SWIEAVGTIAGLLCIGLASLE
O25877 SFINLLAGLSGVLYAFFAGER
Q9CH61 SIITLIATLMGSACTVYMMIG
Q9CK00 SILGMISGISGILCVVFVSKG
P24520 SWIEAVGTIAGLLCIWLASLE
D3QMT7 SWIEAVGTIAGLLCIGLASLE
Q9ZJT8 SFINLLAGLSGVLYAFFAGER
P0AFK2 SWIEAVGTIAGLLCIGLASLE
D2ZZC1 SWLASVAAVTGILCVVFVGKG

PNuC TM2

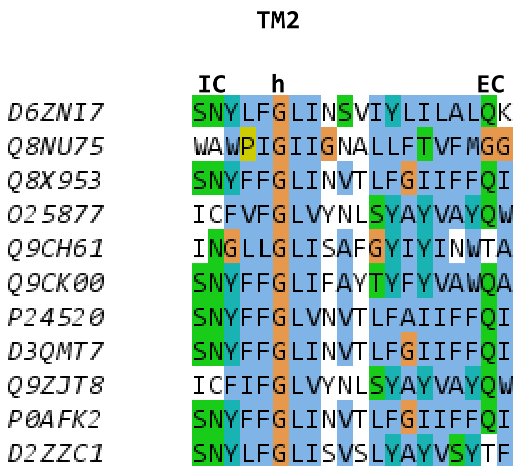

TM2
IC
h
EC
D6ZNI7 SNYLFGLINSVIYLILALQK
Q8NU75 WAWPIGIIGNALLFTVFMGG
Q8X953 SNYFFGLINVTLFGIIFFQI
O25877 ICFVFGLVYNLSYAYVAYQW
Q9CH61 INGLLGLISAFGYIYINWTA
Q9CK00 SNYFFGLIFAYTYFYVAWQA
P24520 SNYFFGLVNVTLFAIIFFQI
D3QMT7 SNYFFGLINVTLFGIIFFQI
Q9ZJT8 ICFIFGLVYNLSYAYVAYQW
P0AFK2 SNYFFGLINVTLFGIIFFQI
D2ZZC1 SNYLFGLISVSLYAYVSYTF

PNuC TM3

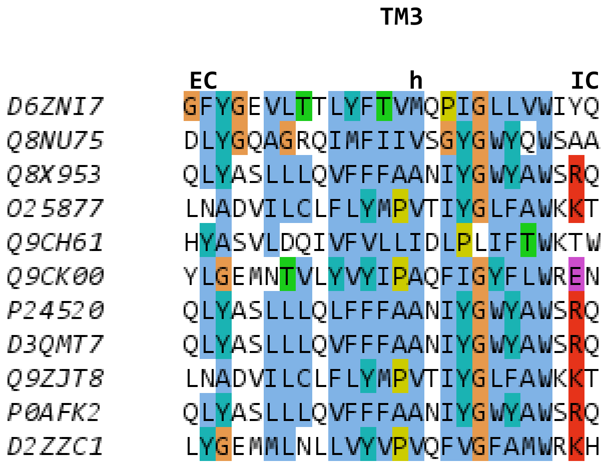

TM3
EC
h
IC
D6ZNI7 GFYGEVLTTLYFTVMQPIGLLVWIYQ
Q8NU75 DLYGQAGRQIMFIIVSGYGWYQWSAA
Q8X953 QLYASLLLQVFFFAANIYGWYAWSRQ
O25877 LNADVILCLFLYMPVTIYGLFAWKKT
Q9CH61 HYASVLDQIVFVLLIDLPLIFTWKTW
Q9CK00 YLGEMNTVLYVYIPAQFIGYFLWREN
P24520 QLYASLLLQLFFFAANIYGWYAWSRQ
D3QMT7 QLYASLLLQVFFFAANIYGWYAWSRQ
Q9ZJT8 LNADVILCLFLYMPVTIYGLFAWKKT
P0AFK2 QLYASLLLQVFFFAANIYGWYAWSRQ
D2ZZC1 LYGEMMLNLLVYVPVQFVGFAMWRKH

PNuC TM4

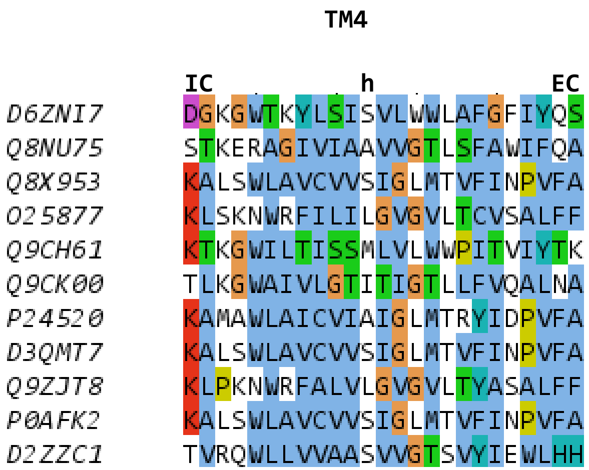
TM4
IC
h
EC
D6ZNI7 DGKGWTKYLSISVLWWLAFGFIYQS
Q8NU75 STKERAGIVIAAVVGTLSFAWIFQA
Q8X953 KALSWLAVCVVSIGLMTVFINPVFA
O25877 KLSKNWRFILILGVGVLTCVSALFF
Q9CH61 KTKGWILTISSMLVLWWPITVIYTK
Q9CK00 TLKGWAIVLGTITIGTLLFVQALNA
P24520 KAMAWLAICVIAIGLMTRYIDPVFA
D3QMT7 KALSWLAVCVVSIGLMTVFINPVFA
Q9ZJT8 KLPKNWRFALVLGVGVLTYASALFF
P0AFK2 KALSWLAVCVVSIGLMTVFINPVFA
D2ZZC1 TVRQWLLVVAASVVGTSVYIEWLHH

PNuC TM5

```
                    TM5

          EC             h      IC
D6ZNI7    PYRDSITDATNGVGQILMTA
Q8NU75    PWADAWIFVGSILATYGMAR
Q8X953    PFWDSCMMVLSIVAMILMTR
O25877    LWAESFNFVIFIIAFILQVL
Q9CH61    PLWDAITLIIGAAASILVVR
Q9CK00    TGLDGLTTVIVVVAQLLMIL
P24520    PFWDSCMMVLSIVAMILMTR
D3QMT7    PFWDSCMMVLSIVAMILMTR
Q9ZJT8    LWAESFNFVIFIIAFILQVL
P0AFK2    PFWDSCMMVLSIVAMILMTR
D2ZZC1    PTLDGVTVVVSIVAQVLMIL
```

PNuC TM6

```
               TM6

           IC     h              EC
D6ZNI7     EQWIFWAATNVFSIYLWWGES--
Q8NU75     EFWLIWIAVDIVGVPLLLTAGYY
Q8X953     ENWLLWVIINVISVVIFALQGVY
O25877     ENYALVTLGNIVSIIVWFCIFQI
Q9CH61     DSYSLWLLSDVVMIILWATALMD
Q9CK00     EQWLLWIALNVISIVLWTQAKEG
P24520     ENWLLWVIINVISVVIFALQGVY
D3QMT7     ENWLLWVIINVISVVIFALQGVY
Q9ZJT8     ENYALVTLGNIVSIIVWFCIFQI
P0AFK2     ENWLLWVIINVISVVIFALQGVY
D2ZZC1     EQWALWIVVNILTISLWAVAWFK
```

PNuC TM7

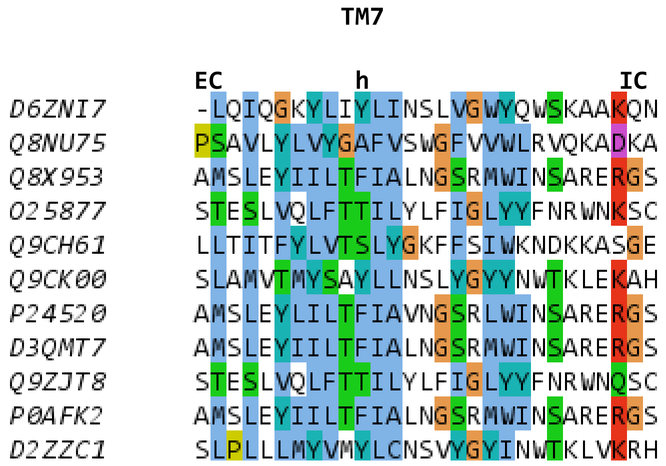
TM7
EC
h
IC
D6ZNI7 -LQIQGKYLIYLINSLVGWYQWSKAAKQN
Q8NU75 PSAVLYLVYGAFVSWGFVVWLRVQKADKA
Q8X953 AMSLEYIILTFIALNGSRMWINSARERGS
O25877 STESLVQLFTTILYLFIGLYYFNRWNKSC
Q9CH61 LLTITFYLVTSLYGKFFSIWKNDKKASGE
Q9CK00 SLAMVTMYSAYLLNSLYGYYNWTKLEKAH
P24520 AMSLEYLILTFIAVNGSRLWINSARERGS
D3QMT7 AMSLEYIILTFIALNGSRMWINSARERGS
Q9ZJT8 STESLVQLFTTILYLFIGLYYFNRWNQSC
P0AFK2 AMSLEYIILTFIALNGSRMWINSARERGS
D2ZZC1 SLPLLLMYVMYLCNSVYGYINWTKLVKRH

SemiSweet TM1

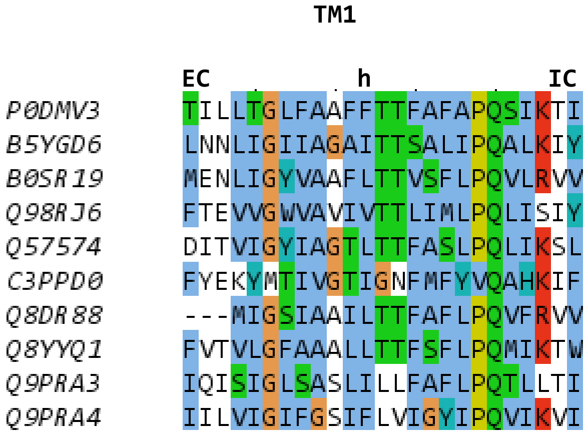

SemiSweet TM2

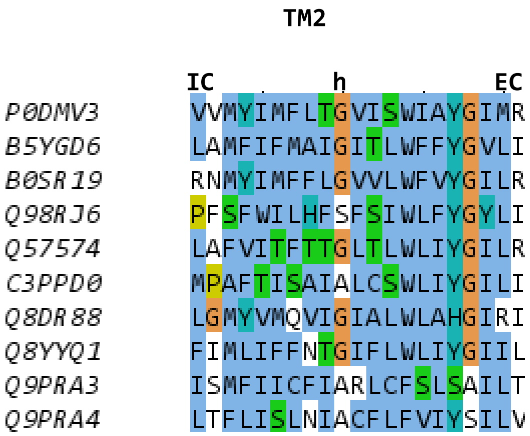

SemiSweet TM3

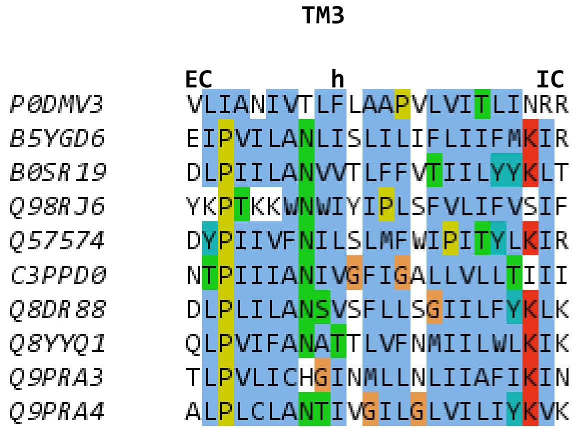

Sweet TM1

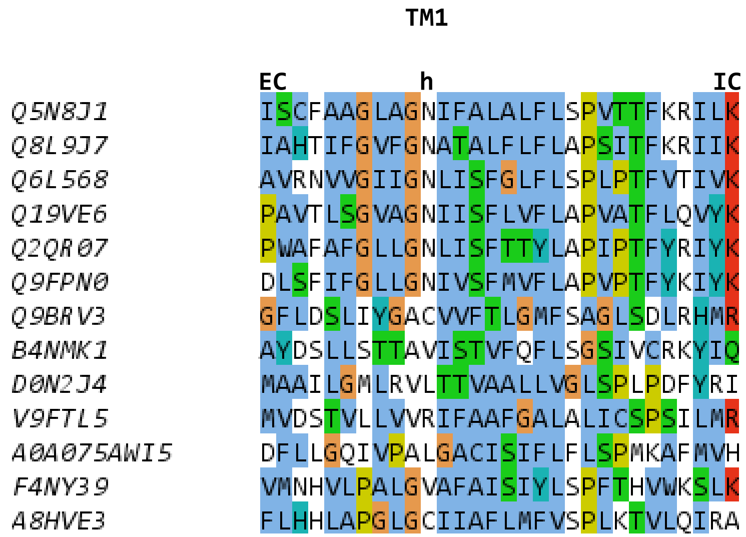
TM1
EC
h
IC
Q5N8J1 ISCFAAGLAGNIFALALFLSPVTTFKRILK
Q8L9J7 IAHTIFGVFGNATALFLFLAPSITFKRIIK
Q6L568 AVRNVVGIIGNLISFGLFLSPLPTFVTIVK
Q19VE6 PAVTLSGVAGNIISFLVFLAPVATFLQVYK
Q2QR07 PWAFAFGLLGNLISFTTYLAPIPTFYRIYK
Q9FPN0 DLSFIFGLLGNIVSFMVFLAPVPTFYKIYK
Q9BRV3 GFLDSLIYGACVVFTLGMFSAGLSDLRHMR
B4NMK1 AYDSLLSTTAVISTVFQFLSGSIVCRKYIQ
D0N2J4 MAAILGMLRVLTTVAALLVGLSPLPDFYRI
V9FTL5 MVDSTVLLVVRIFAAFGALALICSPSILMR
A0A075AWI5 DFLLGQIVPALGACISIFLFLSPMKAFMVH
F4NY39 VMNHVLPALGVAFAISIYLSPFTHVWKSLK
A8HVE3 FLHHLAPGLGCIIAFLMFVSPLKTVLQIRA

Sweet TM2

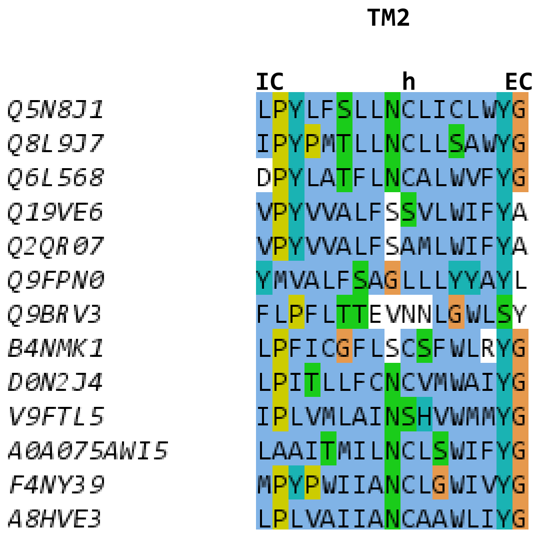
TM2
IC
h
EC
Q5N8J1 LPYLFSLLNCLICLWYG
Q8L9J7 IPYPMTLLNCLLSAWYG
Q6L568 DPYLATFLNCALWVFYG
Q19VE6 VPYVVALFSSVLWIFYA
Q2QR07 VPYVVALFSAMLWIFYA
Q9FPN0 YMVALFSAGLLLYYAYL
Q9BRV3 FLPFLTTEVNNLGWLSY
B4NMK1 LPFICGFLSCSFWLRYG
D0N2J4 LPITLLFCNCVMWAIYG
V9FTL5 IPLVMLAINSHVWMMYG
A0A075AWI5 LAAITMILNCLSWIFYG
F4NY39 MPYPWIIANCLGWIVYG
A8HVE3 LPLVAIIANCAAWLIYG

Sweet TM3

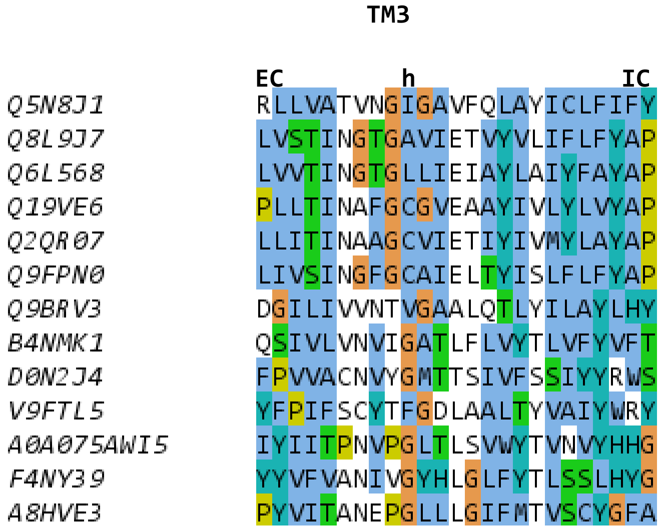
TM3
EC
h
IC
Q5N8J1 RLLVATVNGIGAVFQLAYICLFIFY
Q8L9J7 LVSTINGTGAVIETVYVLIFLFYAP
Q6L568 LVVTINGTGLLIEIAYLAIYFAYAP
Q19VE6 PLLTINAFGCGVEAAYIVLYLVYAP
Q2QR07 LLITINAAGCVIETIYIVMYLAYAP
Q9FPN0 LIVSINGFGCAIELTYISLFLFYAP
Q9BRV3 DGILIVVNTVGAALQTLYILAYLHY
B4NMK1 QSIVLVNVIGATLFLVYTLVFYVFT
D0N2J4 FPVVACNVYGMTTSIVFSSIYYRWS
V9FTL5 YFPIFSCYTFGDLAALTYVAIYWRY
A0A075AWI5 IYIITPNVPGLTLSVWYTVNVYHHG
F4NY39 YYVFVANIVGYHLGLFYTLSSLHYG
A8HVE3 PYVITANEPGLLLGIFMTVSCYGFA

Sweet TM4

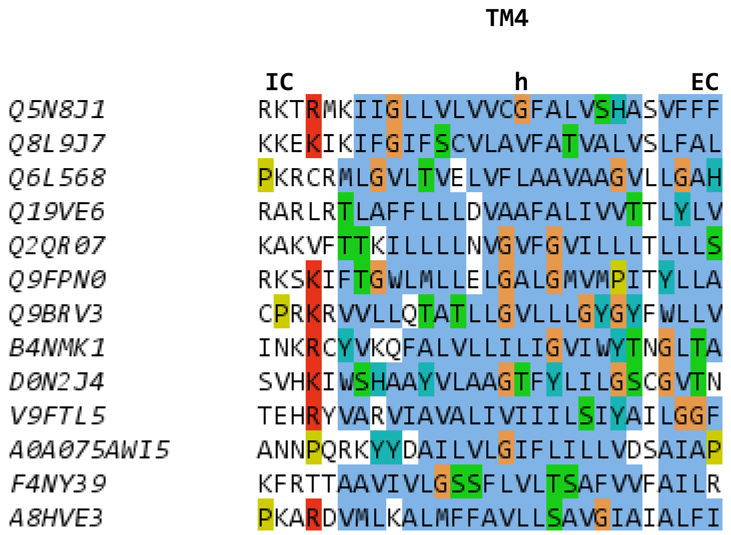
TM4
IC
h
EC
Q5N8J1 RKTRMKIIGLLVLVVCGFALVSHASVFFF
Q8L9J7 KKEKIKIFGIFSCVLAVFATVALVSLFAL
Q6L568 PKRCRMLGVLTVELVFLAAVAAGVLLGAH
Q19VE6 RARLRTLAFFLLLDVAAFALIVVTTLYLV
Q2QR07 KAKVFTTKILLLLNVGVFGVILLLTLLLS
Q9FPN0 RKSKIFTGWLMLLELGALGMVMPITYLLA
Q9BRV3 CPRKRVVLLQTATLLGVLLLGYGYFWLLV
B4NMK1 INKRCYVKQFALVLLILIGVIWYTNGLTA
D0N2J4 SVHKIWSHAAYVLAAGTFYLILGSCGVTN
V9FTL5 TEHRYVARVIAVALIVIIILSIYAILGGF
A0A075AWI5 ANNPQRKYYDAILVLGIFLILLVDSAIAP
F4NY39 KFRTTAAVIVLGSSFLVLTSAFVVFAILR
A8HVE3 PKARDVMLKALMFFAVLLSAVGIAIALFI

Sweet TM5

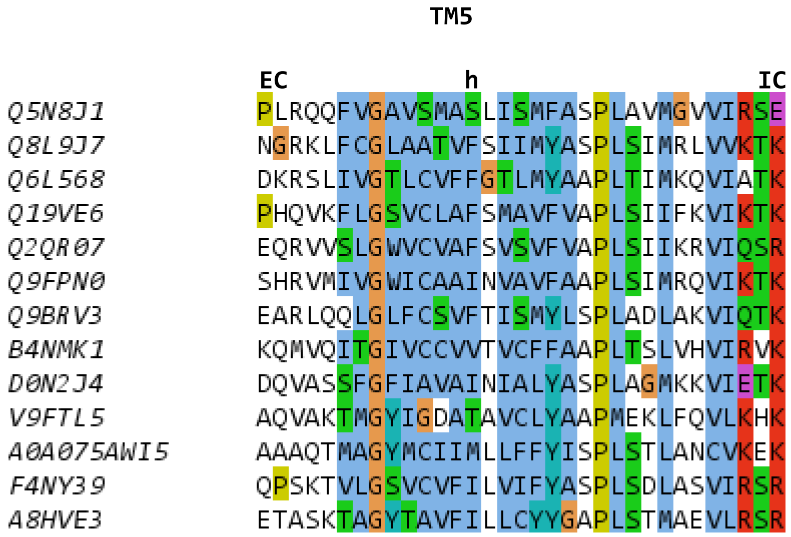
TM5
EC
h
IC
Q5N8J1 PLRQQFVGAVSMASLISMFASPLAVMGVVIRSE
Q8L9J7 NGRKLFCGLAATVFSIIMYASPLSIMRLVVKTK
Q6L568 DKRSLIVGTLCVFFGTLMYAAPLTIMKQVIATK
Q19VE6 PHQVKFLGSVCLAFSMAVFVAPLSIIFKVIKTK
Q2QR07 EQRVVSLGWVCVAFSVSVFVAPLSIIKRVIQSR
Q9FPN0 SHRVMIVGWICAAINVAVFAAPLSIMRQVIKTK
Q9BRV3 EARLQQLGLFCSVFTISMYLSPLADLAKVIQTK
B4NMK1 KQMVQITGIVCCVVTVCFFAAPLTSLVHVIRVK
D0N2J4 DQVASSFGFIAVAINIALYASPLAGMKKVIETK
V9FTL5 AQVAKTMGYIGDATAVCLYAAPMEKLFQVLKHK
A0A075AWI5 AAAQTMAGYMCIIMLLFFYISPLSTLANCVKEK
F4NY39 QPSKTVLGSVCVFILVIFYASPLSDLASVIRSR
A8HVE3 ETASKTAGYTAVFILLCYYGAPLSTMAEVLRSR

Sweet TM6

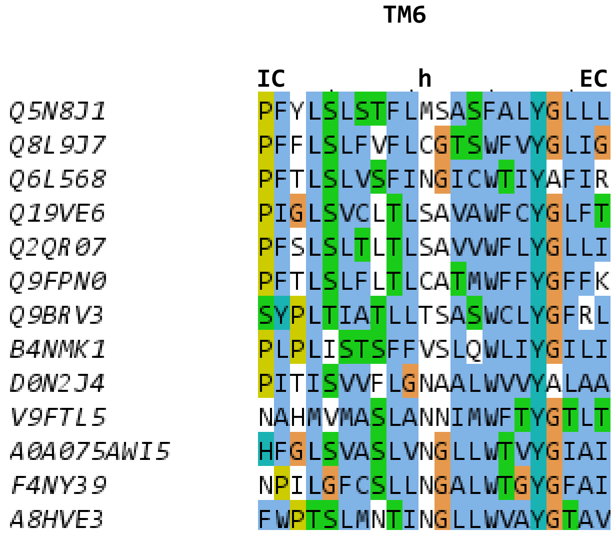

TM6
IC
h
EC
Q5N8J1 PFYLSLSTFLMSASFALYGLLL
Q8L9J7 PFFLSLFVFLCGTSWFVYGLIG
Q6L568 PFTLSLVSFINGICWTIYAFIR
Q19VE6 PIGLSVCLTLSAVAWFCYGLFT
Q2QR07 PFSLSLTLTLSAVVWFLYGLLI
Q9FPN0 PFTLSLFLTLCATMWFFYGFFK
Q9BRV3 SYPLTIATLLTSASWCLYGFRL
B4NMK1 PLPLISTSFFVSLQWLIYGILI
D0N2J4 PITISVVFLGNAALWVVYALAA
V9FTL5 NAHMVMASLANNIMWFTYGTLT
A0A075AWI5 HFGLSVASLVNGLLWTVYGIAI
F4NY39 NPILGFCSLLNGALWTGYGFAI
A8HVE3 FWPTSLMNTINGLLWVAYGTAV

Sweet TM7

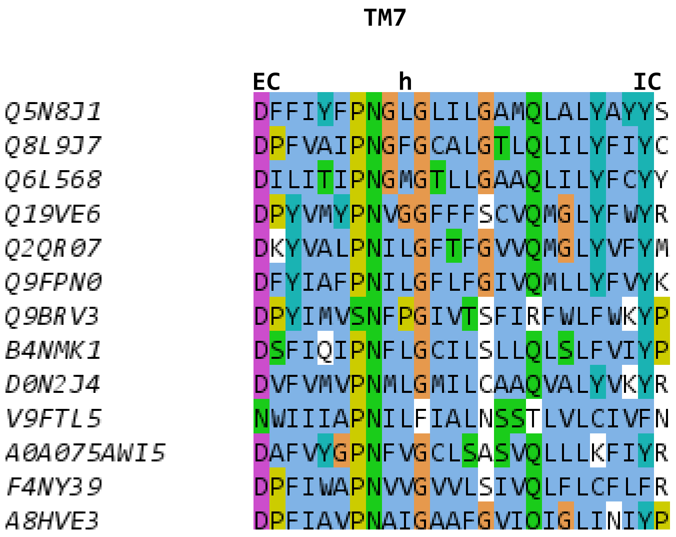
TM7
EC
h
IC
Q5N8J1 DFFIYFPNGLGLILGAMQLALYAYYS
Q8L9J7 DPFVAIPNGFGCALGTLQLILYFIYC
Q6L568 DILITIPNGMGTLLGAAQLILYFCYY
Q19VE6 DPYVMYPNVGGFFFSCVQMGLYFWYR
Q2QR07 DKYVALPNILGFTFGVVQMGLYVFYM
Q9FPN0 DFYIAFPNILGFLFGIVQMLLYFVYK
Q9BRV3 DPYIMVSNFPGIVTSFIRFWLFWKYP
B4NMK1 DSFIQIPNFLGCILSLLQLSLFVIYP
D0N2J4 DVFVMVPNMLGMILCAAQVALYVKYR
V9FTL5 NWIIIAPNILFIALNSSTLVLCIVFN
A0A075AWI5 DAFVYGPNFVGCLSASVQLLLKFIYR
F4NY39 DPFIWAPNVVGVVLSIVQLFLCFLFR
A8HVE3 DPFIAVPNAIGAAFGVIQIGLINIYP

Tric TM1

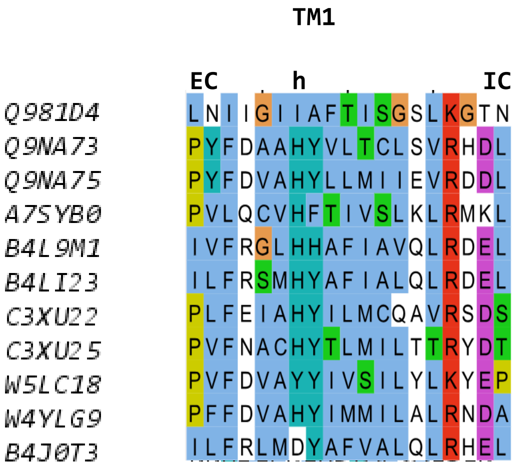
TM1
EC
h
IC
Q981D4 LNIIGIIAFTISGSLKGTN
Q9NA73 PYFDAAHYVLTCLSVRHDL
Q9NA75 PYFDVAHYLLMIIEVRDDL
A7SYB0 PVLQCVHFTIVSLKLRMKL
B4L9M1 IVFRGLHHAFIAVQLRDEL
B4LI23 ILFRSMHYAFIALQLRDEL
C3XU22 PLFEIAHYILMCQAVRSDS
C3XU25 PVFNACHYTLMILTTRYDT
W5LC18 PVFDVAYYIVSILYLKYEP
W4YLG9 PFFDVAHYIMMILALRNDA
B4J0T3 ILFRLMDYAFVALQLRHEL

Tric TM2

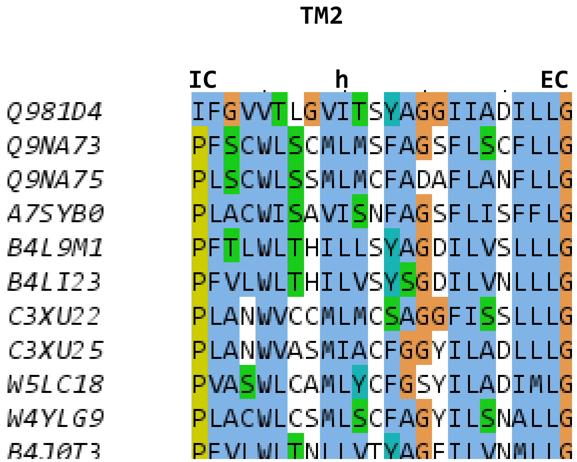
TM2
IC
h
EC
Q981D4 IFGVVTLGVITSYAGGIIADILLG
Q9NA73 PFSCWLSCMLMSFAGSFLSCFLLG
Q9NA75 PLSCWLSSMLMCFADAFLANFLLG
A7SYB0 PLACWISAVISNFAGSFLISFFLG
B4L9M1 PFTLWLTHILLSYAGDILVSLLLG
B4LI23 PFVLWLTHILVSYSGDILVNLLLG
C3XU22 PLANWVCCMLMCSAGGFISSLLLG
C3XU25 PLANWVASMIACFGGYILADLLLG
W5LC18 PVASWLCAMLYCFGSYILADIMLG
W4YLG9 PLACWLCSMLSCFAGYILSNALLG

Tric TM3

```
                 TM3
        EC        h        IC
Q981D4  LNYLLLSVGISIFVFYF
Q9NA73  HADILLGSIVWYLVFYS
Q9NA75  HDDIILATIIWYLVFYA
A7SYB0  VRAVAAMTTIWYLVFYC
B4L9M1  AQDVALCTAAWYIVFYS
B4LI23  YHDVMLSTFAWYIIFYS
C3XU22  TPNVLLSSAIWYLEFYA
C3XU25  PEDVVLASAIWYLLFYC
W5LC18  NSDILLASAVWYLIFFC
W4YLG9  HSKIGTATIVWYMIFYS
B4J0T3  VQDILLCSAAWYLIFYS
```

Tric TM4

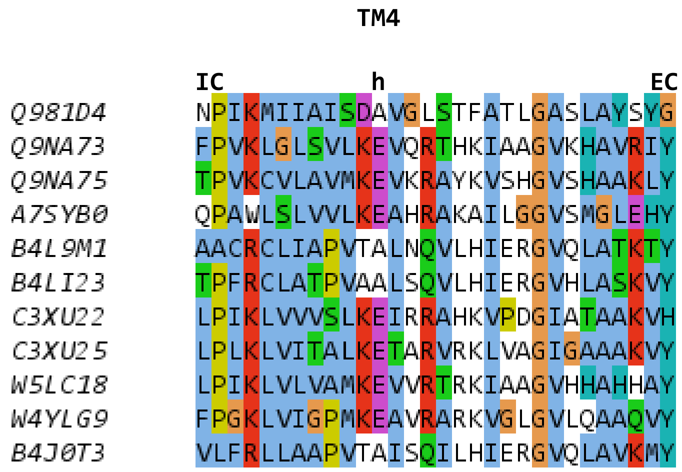

TM4
IC
h
EC
Q981D4 NPIKMIIAISDAVGLSTFATLGASLAYSYG
Q9NA73 FPVKLGLSVLKEVQRTHKIAAGVKHAVRIY
Q9NA75 TPVKCVLAVMKEVKRAYKVSHGVSHAAKLY
A7SYB0 QPAWLSLVVLKEAHRAKAILGGVSMGLEHY
B4L9M1 AACRCLIAPVTALNQVLHIERGVQLATKTY
B4LI23 TPFRCLATPVAALSQVLHIERGVHLASKVY
C3XU22 LPIKLVVVSLKEIRRAHKVPDGIATAAKVH
C3XU25 LPLKLVITALKETARVRKLVAGIGAAAKVY
W5LC18 LPIKLVLVAMKEVVRTRKIAAGVHHAHHAY
W4YLG9 FPGKLVIGPMKEAVRARKVGLGVLQAAQVY
B4J0T3 VLFRLLAAPVTAISQILHIERGVQLAVKMY

Tric TM5

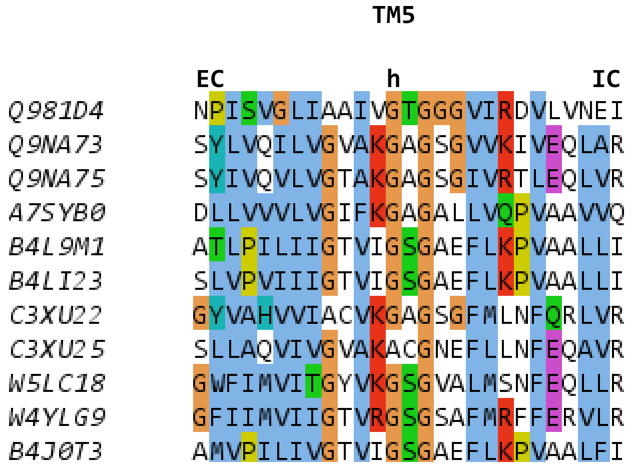
TM5
EC
h
IC
Q981D4 NPISVGLIAAIVGTGGGVIRDVLVNEI
Q9NA73 SYLVQILVGVAKGAGSGVVKIVEQLAR
Q9NA75 SYIVQVLVGTAKGAGSGIVRTLEQLVR
A7SYB0 DLLVVVLVGIFKGAGALLVQPVAAVVQ
B4L9M1 ATLPILIIGTVIGSGAEFLKPVAALLI
B4LI23 SLVPVIIIGTVIGSGAEFLKPVAALLI
C3XU22 GYVAHVVIACVKGAGSGFMLNFQRLVR
C3XU25 SLLAQVIVGVAKACGNEFLLNFEQAVR
W5LC18 GWFIMVITGYVKGSGVALMSNFEQLLR
W4YLG9 GFIIMVIIGTVRGSGSAFMRFFERVLR
B4J0T3 AMVPILIVGTVIGSGAEFLKPVAALFI

Tric TM6

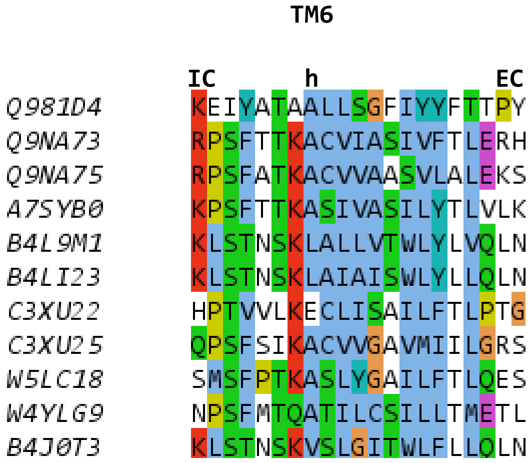

TM6
IC
h
EC
Q981D4 KEIYATAALLSGFIYYFTTPY
Q9NA73 RPSFTTKACVIASIVFTLERH
Q9NA75 RPSFATKACVVAASVLALEKS
A7SYB0 KPSFTTKASIVASILYTLVLK
B4L9M1 KLSTNSKLALLVTWLYLVQLN
B4LI23 KLSTNSKLAIAISWLYLLQLN
C3XU22 HPTVVLKECLISAILFTLPTG
C3XU25 QPSFSIKACVVGAVMIILGRS
W5LC18 SMSFPTKASLYGAILFTLQES
W4YLG9 NPSFMTQATILCSILLTMETL
B4J0T3 KLSTNSKVSLGITWLFLLQLN

Tric TM7

```
                 TM7

       EC          h           IC
Q981D4 SLFVAFLGSFLLRILSIKYNFNLP
Q9NA73 PHDLVYLCVVGFFIYFKLASLCLS
Q9NA75 PHDLVYLVIVGFFVYFKLSAVILH
A7SYB0 STECLCFGVFLVFALIKVSLHVMD
B4L9M1 RRCQLQLYVLLALTIFRILAVFYR
B4LI23 SRCQLQLYALLALMCFKFLALFYR
C3XU22 NQDQLLLLTGIAMVIVKVTLTLTN
C3XU25 SQEMVVLAVSLWLISVAVSMKAFH
W5LC18 SKSTLICIFTLFMATSKVIMTARH
W4YLG9 DAPELMLMLTTLLVGYKMASVVFK
B4J0T3 GKNQLQLYVLFMLMCFKYMVLFYR
```
