# Supplementary material for: Pseudo-Symmetric Assembly of Protodomains as a Common Denominator in the Evolution of Polytopic Helical Membrane Proteins
Source: J Mol Evol. 2020 Mar 18;88(4):319–44. doi: 10.1007/s00239-020-09934-4 (PMC7162841; doi:10.1007/s00239-020-09934-4)
Supplement: Supplementary file 3 — Supplementary file SF2 (PDF 99 kb) [file 239_2020_9934_MOESM3_ESM.pdf]

GPCRs

PDB	Uniprot	use
4PHU	O14842	+
4ZJC	O43613	+
2RH1	P07550	+
4ZWJ	P08100	+
3UON	P08172	+
5DSG	P08173	+
5XCV	P11229	+
4XES	P20789	+
3V2Y	P21453	+
5U09	P21554	+
5GLH	P24530	+
2LNL	P25024	+
3VW7	P25116	+
4IAR	P28222	+
5EML	P29274	+
5UEN	P30542	+
4K5Y	P34998	+
3RZE	P35367	+
3PBL	P35462	+
4N6H	P41143	+
4DJH	P41145	+
4EA3	P41146	+
4OO9	P41594	+
5T1A	P41597	+
5VEW	P43220	+
5EE7	P47871	+
4XNV	P47900	+
5UNG	P50052	+
4MBS	P51681	+
5LWE	P51686	+
5NDD	P55085	+
3ODU	P61073	+
4OR2	Q13255	+
4Z34	Q92633	+
4JKV	Q99835	+
4PXZ	Q9H244	+

CDD Link

NONE, S2Table.csv was used from (Cvicek et al. 2016):
http://www.csun.edu/gpcrs/datas/gross_paper/S2Table.csv

Aquaporins

PDB	Uniprot	Use	gi #
1FQY	P29972	-	14278358
1FX8	P0AER0	-	21466058
1H6I	P29972	+	14278358
1IH5	P29972	-	14278358
1J4N	P47865	-	NONE
1LDA	P0AER0	-	21466058
1LDF	P0AER0	+	21466058
1LDI	P0AER0	-	21466058
1RC2	P60844	-	NONE
1SOR	Q6J8I9	-	NONE
1YMG	P06624	-	NONE
1Z98	Q41372	-	NONE
2ABM	P60844	-	NONE
2B5F	Q41372	-	NONE
2B6O	Q6J8I9	+	NONE
2B6P	Q6J8I9	-	NONE
2C32	P06624	-	NONE
2D57	P47863	-	NONE
2EVU	Q9C4Z5	-	NONE
2F2B	Q9C4Z5	+	NONE
2O9D	P60844	-	NONE
2O9E	P60844	-	NONE
2O9F	P60844	-	NONE
2O9G	P60844	+	NONE
2W1P	F2QVG4	-	NONE
2W2E	F2QVG4	-	NONE
2ZZ9	P47863	-	NONE
3C02	Q8WPZ6	+	NONE
3CLL	Q41372	-	NONE
3CN5	Q41372	+	NONE
3CN6	Q41372	-	NONE
3D9S	P55064	+	NONE
3GD8	P55087	+	NONE
3IYZ	P47863	-	NONE
3J41	Q6J8I9	-	NONE
3LLQ	Q8UJW4	+	NONE
3M9I	Q6J8I9	-	NONE
3NE2	O28846	+	NONE
3NK5	P60844	-	NONE
3NKA	P60844	-	NONE
3NKC	P60844	-	NONE
3ZOJ	F2QVG4	+	NONE
4CSK	P29972	-	14278358
4IA4	Q41372	-	NONE
4JC6	Q41372	-	NONE

4NEF	P41181	+	618855042
4OJ2	P41181	-	618855042
5BN2	F2QVG4	-	NONE
5C5X	P55064	-	NONE
5DYE	P55064	-	NONE
5I32	Q41951	+	NONE
NONE	P23645	+	71153495
NONE	P23900	+	1706896
NONE	P06624	+	85544350
NONE	Q08451	+	586102
NONE	P43286	+	1175013
NONE	P25818	+	138560
NONE	P26587	+	135858
NONE	P08995	+	1352509
NONE	O24389	+	1518057

CDD Link:
https://www.ncbi.nlm.nih.gov/Structure/cdd/cddsrv.cgi?uid=321252

FOCA

PDB	Uniprot	Use	gi#
4FC4	E8XEH9	+	NONE
3TDP	Q186B7	+	NONE
3Q7K	Q7CQU0	+	NONE
3KLY	Q9KRE7	+	NONE
3KCU	P0AC25	+	NONE
NONE	P38750	+	731592
NONE	Q8XCN1	+	156354448
NONE	[illegible]0A1Q4GXT	+	15927981
NONE	Q8DPM4	+	15901077
NONE	Q8ZNA4	+	16765720
NONE	P37327	+	586613
NONE	Q92E59	+	16799677
NONE	W8U1Z5	+	15926006

CDD Link

https://www.ncbi.nlm.nih.gov/Structure/cdd/cddsrv.cgi?uid=COG2116&islf=1

PNuC PDB	Uniprot	Use	gi #
4QTN	D2ZZC1	+	NONE
NONE	D6ZNI7	+	15901687
NONE	Q8NU75	+	19551314
NONE	Q8X953	+	15830033
NONE	O25877	+	15645903
NONE	Q9CH61	+	15672860
NONE	Q9I2E6	+	15597154
NONE	Q9CK00	+	15603703
NONE	P24520	+	20141705
NONE	D3QMT7	+	15800460
NONE	Q9ZJT8	+	15612275
NONE	P0AFK2	+	2507102

CDD Link
https://www.ncbi.nlm.nih.gov/Structure/cdd/cddsrv.cgi?uid=COG3201&islf=1

TRiC				
**PDB**	**Uniprot**	**Use**	**gi #**	
5WTR	Q981D4	+	NONE	
5H35	Q981D4	-	NONE	Not used as better resolution structure avaliable.
5EIK	Q9NA73	+	75023742	
5EGI	Q9NA75	+	NONE	
NONE	A7SYB0	+	156354448	
NONE	A1X7VA	-	761906102	
NONE	B4L9M1	+	195135689	
NONE	B4LI23	+	968115624	
NONE	C3XU22	+	260834779	
NONE	C3XU25	+	260834785	
NONE	W5LC18	+	597742168	
NONE	W4YLG9	+	390366097	
NONE	B4J0T3	+	195011963	

CDD Link

https://www.ncbi.nlm.nih.gov/Structure/cdd/cddsrv.cgi?uid=pfam05197&islf=1

Semi-SWEET

PDB	Uniprot	Use	gi #
5UHS	B0SR19	-	NONE
5UHQ	B0SR19	-	NONE
4X5N	P0DMV3	-	NONE
4X5M	P0DMV3	+	NONE
4RNG	B5YGD6	+	NONE
4QNC	B0SR19	+	NONE
NONE	Q98RJ6	+	15828483
NONE	Q57574	+	2495803
NONE	C3PPD0	+	15892952
NONE	Q8DR88	+	15900237
NONE	Q8YYQ1	+	14195404
NONE	Q9PRA3	+	14195403
NONE	Q9PRA4	+	17228290

CDD Link

https://www.ncbi.nlm.nih.gov/Structure/cdd/cddsrv.cgi?uid=COG4095&islf=1

SWEET		
**PDB**	**Uniprot**	**Use**
5CTG	Q5N8J1	+
5CTH	Q5N8J1	-
NONE	Q8L9J7	+
NONE	Q6L568	+
NONE	Q19VE6	+
NONE	Q2QR07	+
NONE	Q9FPN0	+
NONE	Q9BRV3	+
NONE	Q9XX26	+
NONE	O45102	+
NONE	B4NMK1	+
NONE	D0N2J4	+
NONE	V9FTL5	+
NONE	[illegible]0A075AWI	+
NONE	F4NY39	+
NONE	A8HVE3	+

CDD Link

https://www.ncbi.nlm.nih.gov/Structure/cdd/cddsrv.cgi?uid=pfam03083&islf=1

MFS

PDB	Uniprot	Use	gi #
4PYP	P11166	-	NONE
5EGQ	P11166	-	NONE
5EQH	P11166	+	NONE
5EQI	P11166	-	NONE
4GBY	P0AGF4	+	NONE
4GBZ	P0AGF4	-	NONE
4GC0	P0AGF4	-	NONE
4JA3	P0AGF4	-	NONE
4JA4	P0AGF4	-	NONE
4QIQ	P0AGF4	-	NONE
3Q7P	P11551	-	NONE
3O7Q	P11551	+	NONE
1PW4	P08194	+	34810882
1PV6	P02920	-	34810676
2FCP	P02920	-	34810676
2CFQ	P02920	+	34810676
2V8N	P02920	-	34810676
2Y5Y	P02920	-	34810676
4ZYR	P02920	-	34810676
5GXB	P02920	-	34810676
NONE	P76350	+	2500934
NONE	P0AEX3	+	84029499
NONE	Q5HRH0	+	81174989
NONE	O51798	+	7387890
NONE	P0C0L7	+	81171069
NONE	P37643	+	586685
NONE	P71369	+	7388456
NONE	P94131	+	7387918

CDD Link

https://www.ncbi.nlm.nih.gov/Structure/cdd/cddsrv.cgi?uid=cd06174#seqalign

ACHA7

PDB	Uniprot	Use	gi #
2MAW	P36544	-	NONE
5AFH	P36544	-	NONE
5AFJ	P36544	-	NONE
5AFK	P36544	-	NONE
5AFL	P36544	-	NONE
5AFM	P36544	-	NONE
5AFN	P36544	+	NONE
4PIR	P23979	+	672885916
NONE	P28476	+	410516956
NONE	P24046	+	223590210
NONE	P26714	+	120781
NONE	P19019	+	120772
NONE	P24045	+	120775
NONE	P08220	+	120766
NONE	P25123	+	635377460
NONE	P22933	+	120792
NONE	L8IEJ2	+	440904075

CDD Link

https://www.ncbi.nlm.nih.gov/Structure/cdd/cddsrv.cgi?uid=308533
